# PEARL-Catalyzed Peptide Bond Formation after Chain Reversal by Ureido-Forming Condensation Domains

**DOI:** 10.1021/acscentsci.4c00044

**Published:** 2024-06-03

**Authors:** Yue Yu, Wilfred A. van der Donk

**Affiliations:** † Department of Chemistry and Howard Hughes Medical Institute, University of Illinois at Urbana−Champaign, Urbana, Illinois 61801, United States

## Abstract

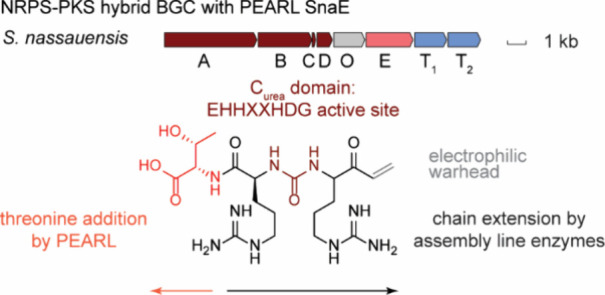

A subset of nonribosomal peptide synthetases (NRPSs) and polyketide synthases (PKSs) are encoded in their biosynthetic gene clusters (BGCs) with enzymes annotated as lantibiotic dehydratases. The functions of these putative lantibiotic dehydratases remain unknown. Here, we characterize an NRPS-PKS BGC with a putative lantibiotic dehydratase from the bacterium *Stackebrandtia nassauensis* (*sna*). Heterologous expression revealed several metabolites produced by the BGC, and the omission of selected biosynthetic enzymes revealed the biosynthetic pathway toward these compounds. The final product is a bisarginyl ureidopeptide with an enone electrophile. The putative lantibiotic dehydratase catalyzes peptide bond formation to a Thr that extends the peptide scaffold opposite to the NRPS and PKS biosynthetic direction. The condensation domain of the NRPS SnaA catalyzes the formation of a ureido group, and bioinformatics analysis revealed a distinct active site signature EHHXXHDG of ureido-generating condensation (C_urea_) domains. This work demonstrates that the annotated lantibiotic dehydratase serves as a separate amide bond-forming machinery in addition to the NRPS, and that the lantibiotic dehydratase enzyme family possesses diverse catalytic activities in the biosynthesis of both ribosomal and nonribosomal natural products.

## Abstract 2

The discovery of threopeptin reveals the function of the putative lantibiotic dehydratases in nonribosomal peptide (NRP) BGCs and enables the bioinformatic prediction of ureido-containing NRPs.

## Introduction

**Figure 1:**
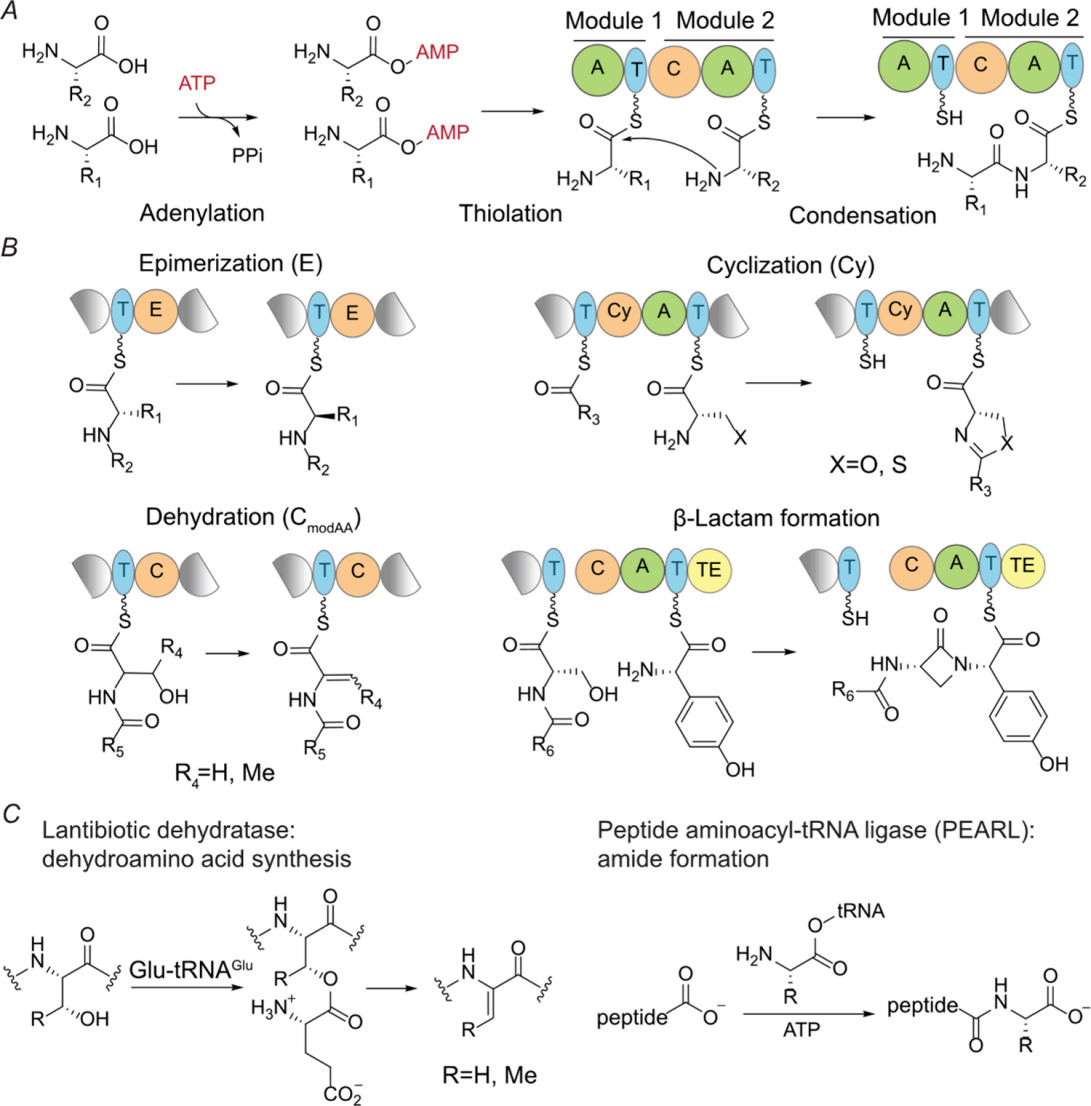
(*A*) Schematic diagram for the function of a typical NRPS peptide elongation module forming a dipeptide bond between two amino acids, here generically drawn with R_1_ and R_2_ side chains. ATP: adenosine-5′-triphosphate, PPi: pyrophosphate. AMP: adenosine-5′-monophosphate. (*B*) Schematic diagram of diverse reactions catalyzed by C domain variants. TE: thioesterase. (*C*) Known reactions catalyzed by class I lantibiotic dehydratases and PEARLs.

Nonribosomal peptides (NRPs) are natural products that possess a range of biological activities, including antibiotic,^1^ anticancer,^2^ biosurfactant,^3^ and immunosuppressant activities.^4^ Their peptide scaffold is biosynthesized by nonribosomal peptide synthetases (NRPSs), multimodular enzymes that work like assembly lines.^5−7^ A typical peptide elongation module consists of three domains for condensation (C), adenylation (A), and thiolation (T). A conserved serine of the T domain is posttranslationally modified by the addition of phosphopantetheine.^8,9^ The A domain activates an amino acid by adenylation and loads it onto the phosphopantetheine arm of the T domain as an acyl-thioester intermediate. The C domain then promotes the formation of peptide bonds by catalyzing the nucleophilic attack of the amine of the acceptor amino acid onto the thioester of the donor amino acid.^10^ (Figure 1*A*). The emergent peptide chain is transferred to the T domain of the following modules after each round of peptide elongation and finally arrives at the terminal module, where a thioesterase domain off-loads the T domain-bound peptide chain via hydrolysis or cyclization.^11^ Other domains or stand-alone enzymes can further tailor the biosynthesized peptide chain before or after off-loading to yield the mature NRP.^12^ Besides peptide bond formation, C domains catalyze other reactions (Figure 1*B*) such as epimerization,^13,14^ peptide bond formation and subsequent epimerization,^15^ heterocyclization,^16^ dehydroamino acid generation,^17,18^ and β-lactam formation.^19^ A C domain variant called the X-domain does not have a catalytic function but recruits P_450_ enzymes during glycopeptide antibiotic biosynthesis.^20^

A small subset of NRPSs are encoded in biosynthetic gene clusters (BGCs) with enzymes annotated as class I lantibiotic dehydratases.^21,22^ These enzymes are composed of two domains: an N-terminal glutamylation domain (protein family PF04738) and a C-terminal elimination domain (protein family PF14028). They generate the dehydroamino acids^23^ of ribosomally synthesized and post-translationally modified peptides (RiPPs),^24^ including lanthipeptides^22^ (called lantibiotics if they display antibiotic activity) and thiopeptides.^25^ The dehydration reaction involves the glutamylation of serine and threonine hydroxyl groups using glutamyl-tRNA^Glu^ catalyzed by the N-terminal domain of the enzyme, and subsequent elimination of glutamate by the C-terminal domain to generate peptidyl dehydroamino acids (Figure 1*C*).^21,26^ Enzymes frequently mis-annotated as lantibiotic dehydratases are peptide aminoacyl-tRNA ligases (PEARLs).^27−30^ PEARLs share sequence homology with the glutamylation domain of lantibiotic dehydratases, but catalyze peptide bond formation at the C-terminus of a carrier peptide using adenosine-5′-triphosphate (ATP) and aminoacyl-tRNA (Figure 1*C*).^28^ The amino acid added by the PEARL typically undergoes further enzymatic modifications and proteolysis to yield an amino acid-derived natural product.^31−33^ However, genes for a RiPP precursor peptide or a cognate PEARL carrier peptide cannot be identified in the NRPS BGCs of interest in this study, indicating the putative lantibiotic dehydratases serve a different function in the biosynthesis of NRPs.

**Figure 2:**
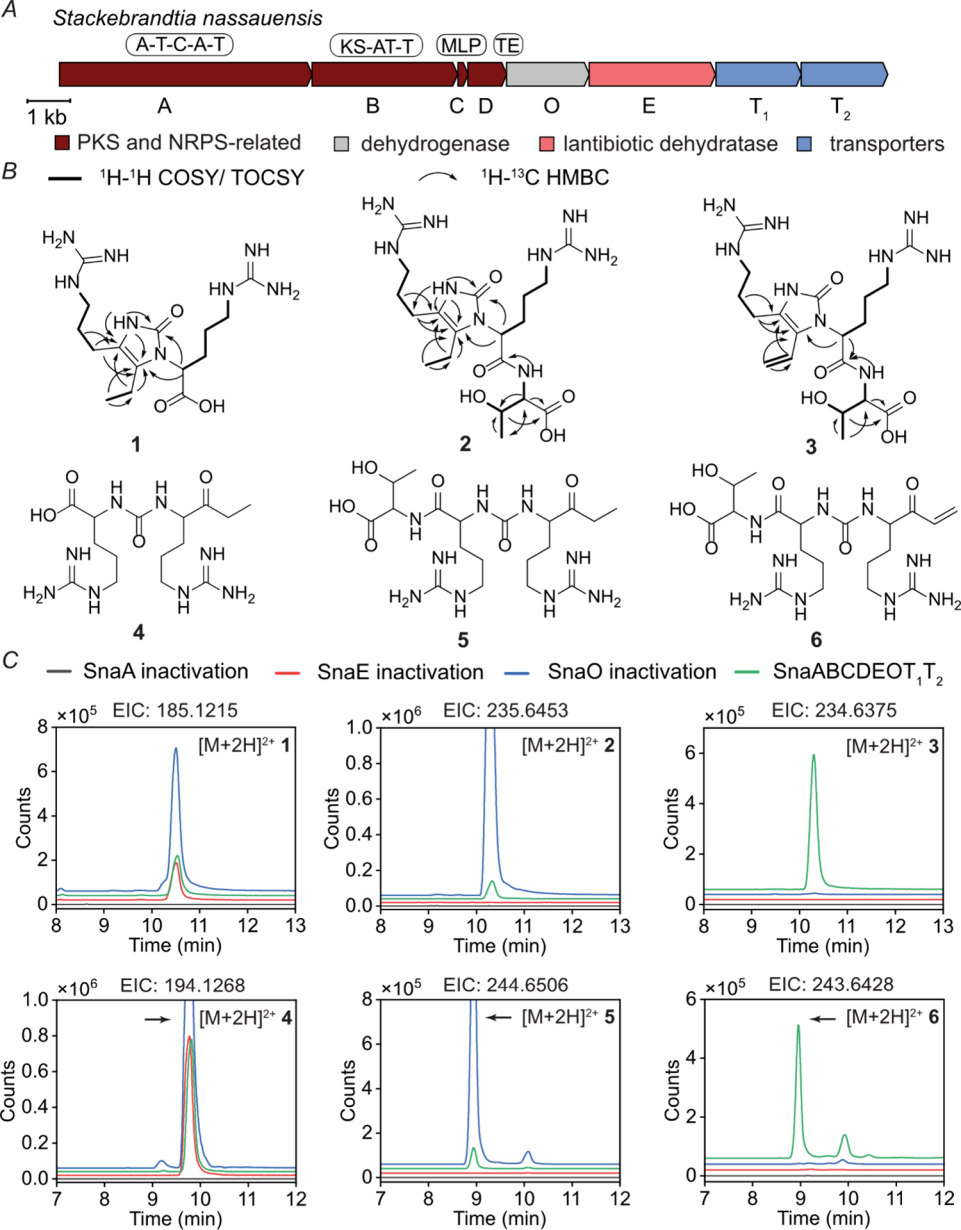
(*A*) Schematic diagram of the *sna* BGC from *S. nassauensis*. For the accession IDs of all proteins in the *sna* BGC, see the Supporting Information Table S2. (*B*) Structures of metabolites produced from the *sna* BGC. Only key NMR connectivities used to solve the structures of compounds **1**-**3** are shown. For complete NMR data, see the Supporting Information. Masses of compounds **4**-**6** were detected in LC-MS experiments, but they eluded purification and NMR characterization. The structural assignments of compounds **4**-**6** are supported by high-resolution MS/MS spectra (Figure S1–S3). (*C*) Extracted ion chromatograms (EICs) of the [M+2H]^2+^ of compounds **1**-**6** and comparison among different heterologous expression constructs. Each EIC trace is shown offset for clarity. The minor peaks in the EICs of compounds **4**–**6** may correspond to the epimer of the arginine ethyl ketone given the relatively acidic α-proton.

In this study, we investigated a hybrid NRPS-PKS BGC^34−36^ from *Stackebrandtia nassauensis* that encodes a putative lantibiotic dehydratase (SnaE, Figure 2*A*). Heterologous expression of the BGC, comparative metabolomics, and structure elucidation revealed a series of novel metabolite products. The biosynthetic pathway was revealed by omitting select biosynthetic enzymes during heterologous expression. The NRPS SnaA links two arginine amine groups through a ureido group, leaving an inert carboxylate at the initiation position that cannot be further extended by the NRPS machinery. The putative lantibiotic dehydratase SnaE catalyzes peptide bond formation at this unactivated carboxylate of the terminal ureido group, achieving chain extension in the opposite direction to NRPS-PKS biosynthesis. The results suggest that the genes annotated as encoding lantibiotic dehydratases that colocalize with NRPS/PKS machinery are involved in amide bond formations that are not amenable to thioester assembly line biochemistry.

Ureido group formation is one of the many reactions catalyzed by C domains during NRP biosynthesis.^37,38^ In vitro studies of SylC in syringolin biosynthesis suggested that the ureido moiety likely originates from bicarbonate.^39^ This unusual head-to-head condensation reaction between two amino acids led us to hypothesize that the condensation domain of the NRPS SnaA is specialized for ureido group formation. Analysis of the C domain sequences associated with ureido-containing NRPs predicts that the active site signature of a ureido-generating condensation (C_urea_) domain is EHHXXHDG (X represents any amino acid) compared to the canonical XHHXXXDG motif for peptide bond formation.^8,10,40^ Condensation domains that do not generate ureido groups in the Minimum Information about a Biosynthetic Gene cluster (MiBiG) database^41^ never possess the EHHXXHDG motif, suggesting the extra conservation of glutamate and histidine residues in the active site of C domains marks the signature for ureido group formation.

## Results

### Products Generated by the *sna* BGC

Around two thousand NRPS/PKS BGCs encode enzymes annotated as lantibiotic dehydratase (NCBI, June 2023). Most of the annotated lantibiotic dehydratases are stand-alone enzymes, but some of them are fused to thioesterase or thiolation domains. No genes encoding potential lanthipeptide precursor peptides can be bioinformatically identified in these BGCs. This observation suggests that these putative lantibiotic dehydratases are involved in NRP or polyketide (PK) biosynthesis rather than lanthipeptide biosynthesis.

The BGCs in question are mostly from Actinobacteria. We chose one representative candidate gene cluster from *S. nassauensis* (Figure 2*A*) that was first described bioinformatically by Singh et al.^35^ for heterologous expression in *Streptomyces albidoflavus* J1074 (formerly *Streptomyces albus* J1074).^42,43^ The *snaA* gene encodes a bimodular NRPS (A-T-C-A-T), and the *snaB* gene encodes a PKS that contains a single ketosynthase (KS)-acyltransferase (AT)-thiolation (T) module. The *snaC* gene encodes a MbtH-like protein (MLP) that has been shown to assist the activity of NRPS A domains.^44,45^ The *snaD* gene encodes a stand-alone thioesterase, s*naO* is annotated as encoding an acyl-CoA dehydrogenase, and *snaE* is annotated as encoding a lantibiotic dehydratase. The *snaT*_*1*_ and *snaT*_*2*_ genes encode two transporters. Expression of a construct containing *snaABCDOET*_*1*_*T*_*2*_ under control of the SP44 constitutive promoter^46^ produced several new metabolites detected by liquid chromatography–mass spectrometry (LC-MS). Expression in two liters of media, purification, and characterization by nuclear magnetic resonance (NMR) spectroscopy revealed the structures of three major metabolites (compounds **1**, **2**, and **3**, Figure 2*B*).

In addition to compounds **1**-**3**, we observed three other products **4**-**6** (Figure 2*B*). Compounds **1** and **4**, **2** and **5**, and **3** and **6** were always produced together, respectively (Figure 2*C*). High-resolution mass spectrometry suggests the difference in molecular formulas of each pair is H_2_O. A spontaneous intramolecular dehydrative cyclization between the ureido NH and the ketone explains the formation of compounds **1**, **2**, and **3** from **4**, **5**, and **6**, respectively. Similar reactions of guanidino nitrogens spontaneously cyclizing onto an arginine ethyl ketone have been observed in the study of saxitoxin biosynthesis.^47−49^ Compounds **4**-**6** eluded spectroscopic characterization because of their high cyclization reactivity during purification efforts, but high-resolution MS/MS spectra (Figure S1-3) as well as observed nonenzymatic conversion of **4** to **1**, **5** to **2**, and **6** to **3** during purification strongly support the structural assignment.

To investigate the enzymes required to produce these compounds, *snaA* (NRPS), *snaO* (dehydrogenase), and *snaE* (putative lantibiotic dehydratase) were individually inactivated during heterologous expression. For *snaA*, both serine codons of the T domains were mutated to alanine to yield inactive mutants that cannot be converted to the holo form. In-frame deletions were used to inactivate *snaO* and *snaE* (Supporting Information). Production of compounds **1**/**4** with two arginines was only abolished by the inactivation of SnaA (Figure 2*C*). Compounds **2**/**5** contain one more Thr than compounds **1**/**4**, and they were not produced when either SnaA or SnaE was inactivated. Compounds **3**/**6** have an additional alkene group compared to compounds **2**/**5**. They required SnaA, SnaE, and SnaO for biosynthesis. These results suggest that compounds **1**/**4** are early stage biosynthetic intermediates or their derivatives produced by the NRPS and PKS (vide infra), and compounds **3**/**6** are likely a later intermediate or the final product. The arginine and threonine residues in compound **3** were determined to have the L configuration based on Marfey’s analysis^50,51^ (Figure S4-5). Structural comparison between **1** and **2** strongly suggests that the putative lantibiotic dehydratase SnaE catalyzes the formation of a peptide bond between a threonine donor and a motif made by the NRPS/PKS. Therefore, SnaE is a peptide bond-forming enzyme rather than a dehydratase as previously annotated.

### Proposed Biosynthetic Pathway

**Figure 3:**
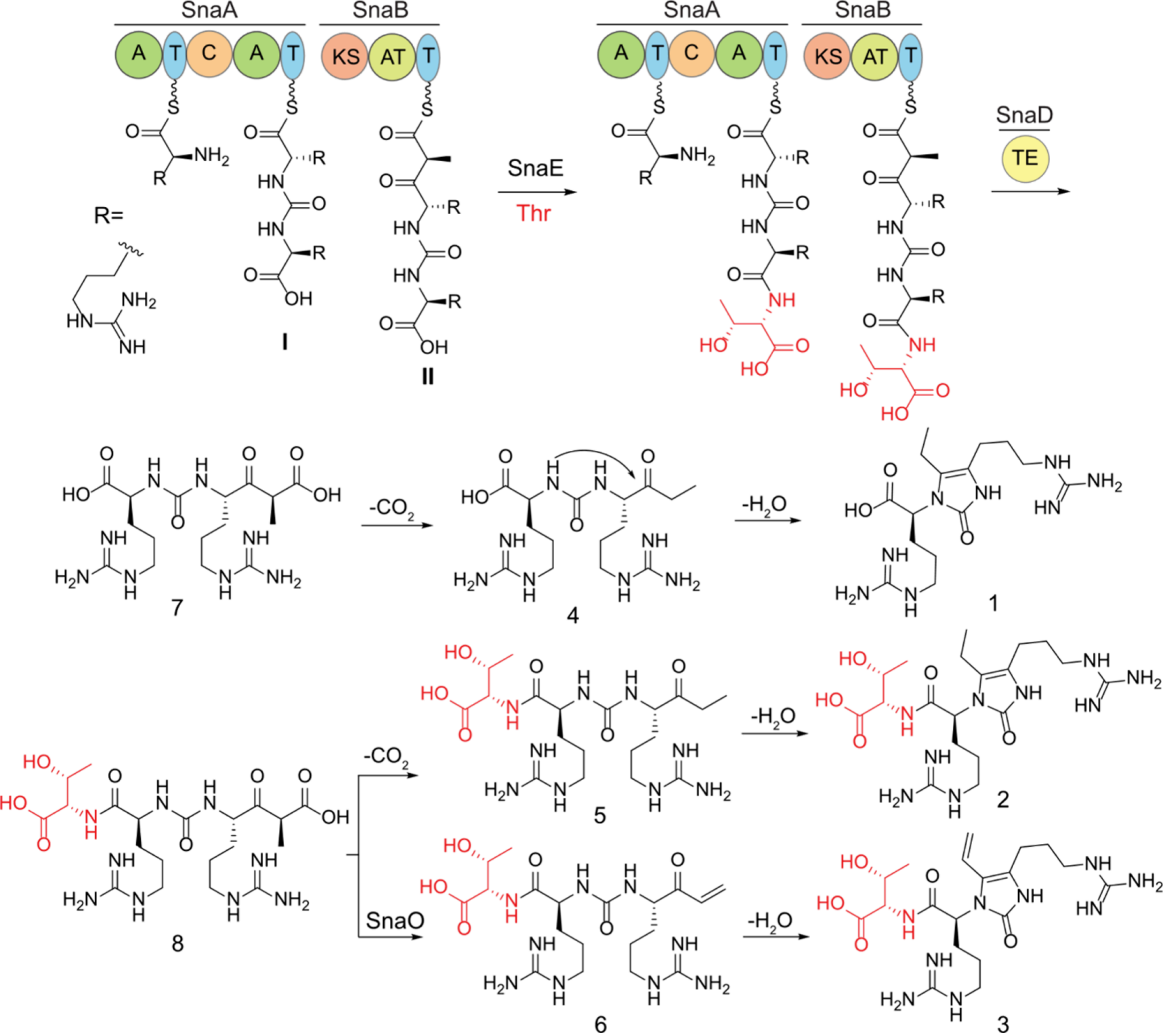
Proposed biosynthetic pathway to generate compound **6** (threopeptin). SnaA links two arginines through a ureido group and SnaB installs a propionate extension unit. The drawing assumes that threonine addition occurs on the assembly line. However, compounds **4**-**7** could also be the substrate of SnaE. SnaO installs the enone by dehydrogenation. Compounds **1**, **2**, and **3** are formed from spontaneous nonenzymatic cyclization from compounds **4**, **5**, and **6**, respectively.

Knowing the required enzymes for the biosynthesis of each metabolite, we propose the following biosynthetic pathway (Figure 3). The two adenylation domains of SnaA (NCBI ADD43706.1) both activate and load arginine onto the T domains as activated thioesters. The condensation domain catalyzes the condensation between two amine groups of arginine to form a ureido group (intermediate **I**) that is likely derived from bicarbonate (HCO_3_^–^).^39^ Current bioinformatics tools, such as antiSMASH,^52^ PRISM,^53^ and AdenylPred,^54^ were used in attempts to predict the reaction catalyzed by the C domain of SnaA and the specificity of the A domains. The C domain of SnaA was classified by antiSMASH as a ^L^C_L_ domain, which promotes peptide bond formation between T domain-bound aminoacyl intermediates with L configuration. Hydrophobic amino acids were predicted by AntiSMASH and PRISM to be the substrate of the two A domains, whereas AdenylPred predicts amino acids with polar and charged side chains to be the substrates, but none predicted Arg as the specific amino acid.

The PKS SnaB (NCBI ADD43707.1) incorporates a propionate extension unit into the growing chain, as shown by isotope enrichment upon feeding 2-^13^C sodium propionate to the heterologous expression system (Figure S6). Therefore, the substrate for the acyltransferase domain of SnaB is likely methylmalonyl-CoA, although antiSMASH predicts malonyl-CoA as the SnaB substrate. The SnaB-bound intermediate **II** may be hydrolyzed by the thioesterase SnaD (NCBI ADD43709.1) to form compound **7**, which upon decarboxylation gives compound **4**. Threonine addition by SnaE (NCBI ADD43711.1) can occur on T domain-bound intermediates **I** or **II** (Figure 3) or on the free molecules **4** or **7**. Based on precedence with the dehydrogenase EpnF,^55,56^ compound **6** is likely produced from compound **8** by SnaO (NCBI ADD43710.1) via a decarboxylation-dehydrogenation reaction sequence, but conversion of compound **5** to **6** by SnaO cannot be ruled out. As outlined in the Discussion section, we consider compound **6** the final product of the pathway and term this compound threopeptin, whereas the formation of compounds **1**-**5** are proposed to be off pathway via nonenzymatic cyclization and/or premature thioesterase activity.

### Bioinformatic and Biochemical Studies on Ureido Group Formation

**Figure 4:**
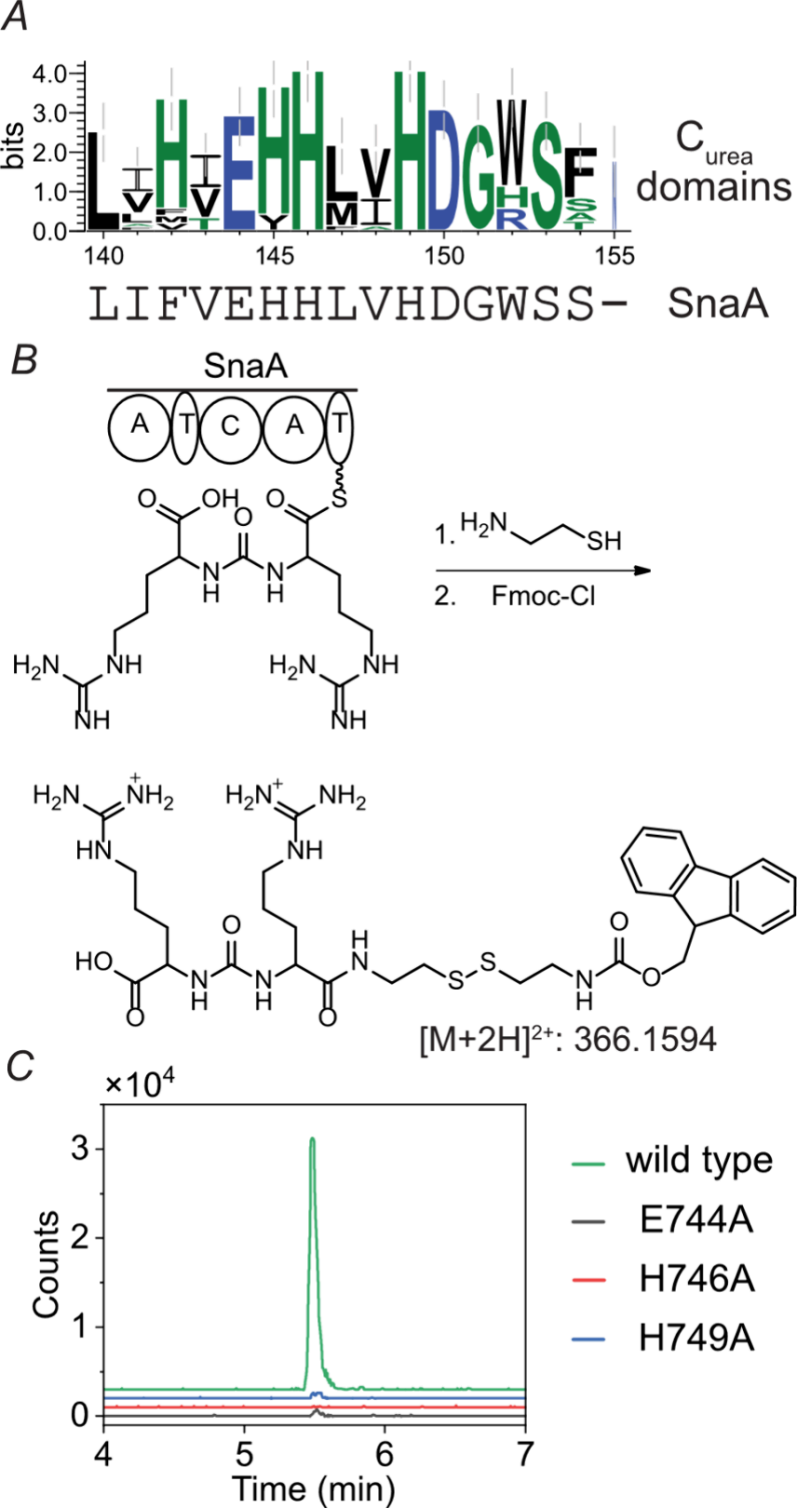
(*A*) Multiple sequence alignment of C_urea_ domains. The logo was generated by WebLogo 3.^71^ The Supporting Information Table S3 contains the MiBiG BGC repository identification numbers for the listed enzymes. The fully conserved Ser153 in the alignment of C_urea_ sequences (Ser753 in SnaA) is usually not included in prior signature motifs of C domains and is also found in amide forming domains. (*B*) Scheme of the derivatization of the bisarginine ureido structure generated by SnaA in vitro. (*C*) EICs of Fmoc- and cysteamine-derivatized bisarginine ureido structure generated in vitro by wild type and mutant SnaA.

Ureido group formation is one of the many reactions catalyzed by C domains during NRP biosynthesis.^37,38^ Based on current understanding, we could not have predicted that the *sna* BGC would produce a ureido structure. Therefore, we investigated whether the C_urea_ domains have unique signatures that may assist future annotation. We generated a phylogenetic tree containing C domains associated with ureido-containing NRPs in the MiBiG database^41^ (Figure S7). The C domains group according to their catalytic functions and not amino acid specificity, similar to what has been observed in previous studies.^18,57,58^ The ureido formation activity for some enzymes in the C_urea_ group has been confirmed in previous studies (e.g., syringolin A,^39^ pacidamycin,^59^ antipain,^60^ and muraymycin^60^). Other sequences in the C_urea_ group belong to modular NRPSs and their domain arrangements are consistent with the collinearity to product structures (e.g., anabaenopeptins,^61^ bulbferamide,^62^ chitinimide,^63^ and pseudovibriamide^64^). In these BGCs, the specificity of the A domain of the loading A-T didomain usually corresponds to the amino acid at the terminal position of the ureido group.^60−62,64^ Similarly, the A domain specificity of the first extension module (C_urea_-A-T) usually corresponds to the internal amino acid of the ureido group.^60−62,64^ Multiple sequence alignment showed that C_urea_ domains have a conserved EHHXXHDG active site (Figure 4*A*) compared to the canonical XHHXXXDG active site of amide bond-forming C domains.^8,40^ Examining all C domain sequences in MiBiG showed that none of the C domains with other functions have an EHHXXHDG active site. Therefore, based on current examples, the EHHXXHDG signature of the C domain active site appears to be sufficient and necessary to indicate ureido formation activity. Based on the current phylogenetic analysis with a limited number of examples, ureido forming C domains are closely related to ^L^C_L_ domains.

The ureido-forming activity of SnaA was confirmed in vitro using holo-SnaA heterologously expressed in *Escherichia coli* BAP1.^65^ The T domain-bound products of SnaA were intercepted using cysteamine^66^ followed by chemical derivatization with fluorenylmethyloxycarbonyl chloride (Fmoc-Cl) and LC-MS analysis^67^ (Figure 4*B*). Since SnaA showed little to no activity in the absence of the MLP SnaC, SnaC was included in all in vitro enzyme assays. The observation of a bisarginyl ureido product (Figure 4*C*) confirmed that SnaA is responsible for formation of intermediate **I** (Figure 3). When the active site glutamate (E744) and histidine (H749) residues of the C_urea_ domain (E^744^HH^746^LVH^749^DG) of SnaA were individually mutated to alanine, the resulting mutants led to reduced in vitro production of the arginine ureido dipeptide that was at the detection limit (Figure 4*C*), indicating that the conserved glutamate and histidine residues in the C_urea_ active site are important for the ureido bond-forming activity of SnaA. Based on an AlphaFold^68^ model (Figure S8), it is possible that E744 and H749 may form a salt bridge to help conserve the active site structure for ureido formation and may not participate directly in catalysis. Mutation of the second histidine (H746) that typically serves as the catalytic base for amide bond formation^10,40,69,70^ to alanine abolished product formation (Figure 4*C*), suggesting also an essential role for H746 in the ureido group-forming activity of SnaA. When the active site serine residues of the two T domains were individually substituted with alanine, product formation was abolished but cysteamine-intercepted arginine-thioester intermediates were still observed after Fmoc-derivatization (Figure S9). This observation strongly suggests that arginine is loaded onto both T domains, and that both T domains are required for the ureido-generating activity of SnaA.

## Discussion

The formation of a ureido group during NRP biosynthesis is termed a chain-reversal event because it generates a carboxylate rather than the usual amine group at the initiation position. Proposed mechanisms of ureido group formation from a module containing two T domains like SnaA are presented in Figure S10A-C. The in vitro experiments with SnaA mutants show that both T domains are loaded with Arg. In the first mechanism (Figure S10A), a Leuch’s anhydride-like species is invoked to activate an initially formed *N*-carboxy arginine for ureido group formation, a mechanism reminiscent of what has been proposed for SylC.^39^ In the second mechanism (Figure S10B), an initial condensation reaction involving a T domain-bound *N*-carboxy arginine is followed by rearrangement to form the ureido group. Regardless of the mechanism, after ureido bond formation, the amino acid at the internal position is still attached to the T domain as a thioester (e.g., Figure 3, Intermediate I) and can be further extended by NRPS/PKS biochemistry.^60^ However, the amino acid originally loaded by the first A domain is left with an unactivated carboxylate and can no longer be extended by the assembly line chemistry.^60^ This model explains why all previously isolated ureido-containing NRPs only have one side of the ureido moiety further extended by the NRPS/PKS (Figure S11). If chain extension of the terminal carboxylate is desired for biological activity, two possible solutions can be envisioned. The ureido forming process could proceed through an intermediate where the amino acid loaded by the first A domain remains T domain-bound, thus allowing NRPS-type chain extension in both directions (Figure S10C). This mechanism likely requires ATP activation of bicarbonate, which has been ruled out in the case of SylC,^39^ and hence this possibility seems unlikely. Alternatively, the system would need a separate amidation machinery in addition to the NRPS/PKS assembly line. The PEARL-like enzyme SnaE appears to have been recruited for this latter purpose. Based on its sequence homology to PEARLs, SnaE is likely to add threonine using a similar ATP- and aminoacyl-tRNA-dependent mechanism,^28^ in which ATP is used to phosphorylate the terminal carboxylate to form an activated acyl-phosphate intermediate, which is then attacked by Thr-tRNA^Thr^ as the Thr donor in a condensation reaction. Hydrolysis of the tRNA as demonstrated for PEARLs would then provide the observed products.

PEARLs and the glutamylation domains in class I lantibiotic dehydratases use aminoacyl-tRNA as substrates and share conserved residues that are proposed to bind the 5′-phosphate of the aminoacyl-tRNA^30,72^ (Figure S12). PEARLs possess additional conserved residues (Figure S12) for the ATP-dependent phosphorylation activity.^30^ Since PEARLs are not involved in dehydroamino acid synthesis, they do not co-occur with the elimination domains (PF14028) of lantibiotic dehydratases in the genome neighborhood. Therefore, sequence analysis and genome neighborhood analysis can distinguish PEARLs from lantibiotic dehydratases. The previously characterized lantibiotic dehydratases and PEARLs modify ribosomally produced peptides that are typically 5–10 kDa (Figure 1*C*), whereas SnaE acts on a short nonribosomally produced substrate (Intermediate **I**/**II** or compounds **4**/**7** in Figure 3), which is only present after the chain reversal by SnaA. SnaE is likely the founding member of a broader class of PEARL-like amidation enzymes in NRP biosynthetic pathways. In addition to the mis-annotation of SnaE as lantibiotic dehydratase in databases, the C_urea_ domain of SnaA and other C_urea_ domains in MiBiG (Table S3) are annotated as ^L^C_L_ domains, which catalyze amide bond formation between L-aminoacyl thioesters. We anticipate that the unique active site signatures of C_urea_ domains presented in this study will help more accurately predict ureido-group containing NRPs.

The formation of the ethyl ketone in compounds **4** and **5** follows a unique mechanism where the ethyl group originates from the decarboxylation of methylmalonate. Ethyl ketones are commonly observed motifs during PK/NRP biosynthesis, but the biosynthetic precursors of the ethyl group are usually *S*-adenosyl methionine (SAM) and malonate. For instance, the ethyl ketone derivative of arginine is a biosynthetic intermediate of saxitoxin^47,48^ and is biosynthesized by a polyketide-like synthase SxtA.^73^ Malonyl-CoA is loaded onto the ACP and is then methylated by the methyltransferase domain of SxtA. The methylmalonyl-ACP is thought to be decarboxylated to propionyl-ACP, which is followed by a pyridoxal phosphate-dependent condensation between arginine and propionyl-ACP to yield the arginine ethyl ketone.

In the case of epoxyketone proteasome inhibitors such as epoxomicin and eponemycin,^74−76^ the ethyl groups of the epoxyketone warhead originate similarly from malonyl-CoA and on-ACP methylation(s) by a methyltransferase domain of the PKS EpxE/EpnH.^55,77^ The epoxide is thought to be generated by a conserved acyl-CoA dehydrogenase-like enzyme EpxF/EpnF via a decarboxylation-dehydrogenation-epoxidation sequence after thioesterase-mediated release from the assembly line.^55^ Given that the vinylketone in compounds **3**/**6** appears to originate from methylmalonate, the acyl-CoA dehydrogenase-like enzyme SnaO may also use a decarboxylative dehydrogenation mechanism to install the α,β-unsaturated ketone. Interestingly, the reaction of SnaO seems to stop at dehydrogenation, because no epoxidation was observed during heterologous expression in *S. albidoflavus*.

Different from the biosynthetic pathways of saxitoxin and epoxyketones, the methyltransferase domain for the methylation of malonate is absent in the *sna* BGC. This absence is consistent with the PKS SnaB using methylmalonyl-CoA to produce the ethyl group of threopeptin. The ethyl group of the epoxyketone macyranone could also originate from methylmalonate since its biosynthetic PKS module also lacks a methyltransferase domain.^75^

We hypothesize that compound **6** (threopeptin) is the final product of the *sna* BGC because its biosynthesis depends on SnaA, SnaO, and SnaE, and it carries an α,β-unsaturated ketone that could function as an electrophilic warhead. The antipain group of protease inhibitors^78^ (Figure S11) structurally resembles threopeptin. The aldehyde of antipain covalently targets protease active site serine or cysteine residues and the vinyl ketone of threopeptin may similarly target a protease active site serine or cysteine residue via 1,4-conjugate addition. Consistent with the linear threopeptin being the final desired product, cyclized threopeptin (compound **3**) did not exhibit growth inhibition of *Lactococcus lactis subsp. cremoris*, *Bacillus subtilis*, *Micrococcus luteus*, and *E. coli* MG1655 at concentrations up to 1 mM. Although the instability of threopeptin prevented isolation and bioactivity testing, *S. nassauensis* may produce the compound to inhibit proteases of competitor or predator organisms after secretion by SnaT_1_ and T_2_.

## Data Availability

The authors declare that the data supporting the findings of this study are available within the paper and its Supporting Information files, and at Mendeley Data, V1, doi: 10.17632/rjytc5c3cr.1 as well as from the corresponding author upon reasonable request.

## Supporting Information Available

Procedures for all experiments; MS, stereochemistry, structural and phylogenetic data in Figures S1–S12; nucleotide sequences, accession numbers, and sequence motifs in Tables S1–S4; NMR spectra of new compounds in Figures S14–32 (PDF)
DatasetS1 seq alignment input (TXT)
DatasetS2 tree (TXT)
Transparent Peer Review report available (PDF)
