# Supplementary material for: PEARL-Catalyzed Peptide Bond Formation after Chain Reversal by Ureido-Forming Condensation Domains
Source: ACS Cent Sci. 2024 Jun 3;10(6):1242–50. doi: 10.1021/acscentsci.4c00044 (PMC11212132; doi:10.1021/acscentsci.4c00044)
Supplement: Supplementary file 1 — oc4c00044_si_001.pdf [file oc4c00044_si_001.pdf]

Supporting Information

PEARL-catalyzed peptide bond formation after chain reversal by ureido-forming condensation domains

Yue Yu and Wilfred A. van der Donk*

Department of Chemistry and Howard Hughes Medical Institute, University of Illinois at Urbana-Champaign, Urbana, IL 61801

Email: vddonk@illinois.edu

General Methods:

All oligonucleotides used in this study were purchased from Integrated DNA Technologies. Adenosine-5'-triphosphate, apramycin, kanamycin, and isopropyl β-d-1-thiogalactopyranoside were purchased from GoldBio. Sodium acetate-$^{13}C_2$, cysteamine, magnesium chloride, and fluorenylmethyloxycarbonyl chloride (Fmoc-Cl) were purchased from Sigma-Aldrich. Sodium propionate (2-^{13}C) was purchased from Cambridge Isotope Laboratories. Liquid chromatography-high-resolution mass spectrometry data was recorded on an Agilent 1260 Infinity II coupled to a diode array detector WR and a G6545B mass spectrometer. Purine and HP-0921 (Agilent Technologies, Santa Clara, CA, USA) were used as reference masses during acquisition. Spectra were processed using the Agilent MassHunter Workstation Qualitative Analysis software (version 10.0). Nuclear magnetic resonance data was either acquired on a Bruker Avance III HD equipped with a 500-MHz, 5-mm, BBFO CryoProbe or a spectrometer equipped with a Bruker Avance Neo console and 600-MHz, 5-mm, BBO-BB Prodigy CryoProbe. NMR spectra were referenced to residual NMR solvent signals of dimethylsulfoxide (2.50 ppm, ^{1}H; 39.5 ppm, ^{13}C).

Cloning of *sna* BGC and the selective inactivation of biosynthetic enzymes:

The 17-kilobase (kb) BGC was divided into three fragments for amplification from the genomic DNA of *Stackebrandtia nassauensis* by NEB Q5 high-fidelity DNA polymerase using the primer pairs Sna1F/R, Sna2F/R, and Sna3F/R (Table S1). The expression vector pOSV801 was linearized using the primer pairs Sna_OSVF/R. The plasmid was assembled from three BGC DNA fragments and a linear fragment of pOSV801 with a strong promoter SP44[1] using Gibson Assembly.[2] The sequence of the plasmid termed pOSV801-SP44-Sna was verified using Sanger sequencing by primer walking.

The DNA sequence of SP44:
tgttcacattcgaaccgtctctgctttgacaacatgctgtgcggtgttgtaaagtctggtgtgaccctaacgaggagatcggttcacccat

For inactivation of SnaA during heterologous expression, both active site serine codons (S556 and S1616) in the two peptidyl carrier proteins were mutated to alanine (GCC). pOSV801-SP44-Sna was digested with NheI (ThermoFisher FD0974) and FspAI (ThermoFisher FD1664), and the 15-kb digested fragment without SnaA was purified by DNA gel electrophoresis. The sequence of the other fragment containing SnaA was divided into three pieces based on the location of the conserved serine codon of the PCP. The three fragments were amplified by Q5 DNA polymerase, and alanine mutations were introduced by the primer pairs $SnaA_{in}$_1F/R, $SnaA_{in}$_2F/R, and

$SnaA_{in}$_3F/R (Table S1). The 15-kb fragment without SnaA from restriction digestion and the three PCR fragments with alanine mutations were assembled by Gibson assembly.

For inactivation of SnaB during heterologous expression, the active site serine (S927) in the acyl carrier protein (ACP) was mutated to alanine (GCC). pOSV801-SP44-Sna was digested with EcoRV (NEB R0195S) and FspAI (ThermoFisher FD1664), and the 14-kb digested fragment without the ACP of SnaB was purified by DNA gel electrophoresis. The rest of the plasmid was amplified using primer pairs $SnaB_{in}$_1F/R and $SnaB_{in}$_2F/R (Table S1). The primers similarly introduced the alanine mutation during PCR. The 14-kb fragment without the ACP of SnaA and the two fragments with alanine mutations were assembled by Gibson assembly.

For the inactivation of SnaE and SnaO, in-frame deletion was used. The open reading frame of SnaE was shortened from 2538 bp to 270 bp by retaining only the first 135 base pair (bp) and the last 135 bp of the DNA sequence of SnaE. This will result in a nonfunctional SnaE transcript in the expression plasmid. The expression plasmid with SnaE inactivation was constructed using a 3-fragment Gibson assembly after amplification of pOSV801-SP44-Sna using primer pairs $SnaE_{in}$_1F/R, $SnaE_{in}$_2F/R, and $SnaE_{in}$_3F/R (Table S1).

The inactivation of SnaO was similarly achieved by retaining the first 150 bp and the last 129 bp of its DNA sequence. This truncates the open reading frame of SnaO from 1662 bp to 279 bp and will result in a nonfunctional SnaO. The parent plasmid pOSV801-SP44-Sna was digested by EcoRV (NEB R0195S) and FspAI (ThermoFisher FD1664) and the 14-kb fragment without SnaO was purified by DNA gel electrophoresis. The expression plasmid with SnaO inactivation was constructed using a 3-fragment Gibson assembly from the 14-kb EcoRV-FspAI fragment and two fragments from the amplification of pOSV801-SP44-Sna using primer pairs $SnaO_{in}$_1F/R, $SnaO_{in}$_2F/R (Table S1).

Metabolomic identification of the products of *sna* BGC:

The expression plasmid for the *sna* BGC and derivatives thereof were used to transform *E. coli* ET12567/pUZ8002. The plasmids were then transferred into *S. albidoflavus* J1074 by intergeneric conjugation according to previously described procedures.[3] In an 18×150 mm culture tube, 3 μL of spore stocks of *S. albidoflavus* exconjugants were inoculated into 4 mL of R5 medium.[4] The culture was grown on a rotating tube roller at 30 °C for 4 days. After 4 days, the supernatant and the cell pellet were separated by centrifugation under 4500 rpm for 5 min. The spent medium supernatant was purified using a 100 mg C18 solid phase extraction column (ThermoFisher 60108-302). The column was conditioned with methanol and equilibrated with 2 column volumes (CV) of H_2O. The medium was passed through the C18 SPE column using a vacuum manifold. The SPE column was again washed with 2 CV of H_2O. Bound metabolites were eluted using 350 μL of 50% acetonitrile in H_2O and 150 μL of acetonitrile. The cell pellet was lyophilized to dryness and was extracted with 1 mL of acetone. The acetone extract was evaporated using a SpeedVac vacuum concentrator (SPD140DDA), and the residue was redissolved in 200 μL of MeOH.

The spent medium extract was analyzed on an Agilent 1260 Infinity II coupled with a G6545B liquid chromatography-mass spectrometry (LC-MS) instrument. Each sample was analyzed on a polar C18 column (Phenomenex 00F-4759-AN) maintained at 45 °C using gradient one and on a hydrophilic interaction chromatography (HILIC) column (Phenomenex 00B-4461-AN) maintained at 40 °C using gradient two.

Gradient one:

Solvent A: H_2O + 0.1% formic acid. Solvent B: acetonitrile + 0.1% formic acid

0% B at 0-2 min; 0% B to 60% over 18 min; 60% B to 90% B over 4 min; 90% B to 95% B over 0.5 min; 95% B to 0% B over 1 min. The column was re-equilibrated at 0% B between injections for 4 min.

Gradient two:

Solvent A: H_2O + 10 mM ammonium formate, pH 3.2. Solvent B: 95% acetonitrile in H_2O + 10 mM ammonium formate, pH 3.2.

100% B at 0-1 min; 100% B to 60% over 12 min; 60% B to 100% B over 0.5 min. The column was re-equilibrated at 100% B between injections for 2.8 min.

MS settings:

ion polarity: positive; mass range: 50-1700 m/z; slicer mode: high resolution, 2 GHz; gas temperature: 325 °C; drying gas: 10 L/min; nebulizer pressure: 35 psi; sheath gas temperature 375 °C; sheath gas flow 11 L/ min; capillary voltage: 3500 V; nozzle voltage: 0 V; fragmentor voltage: 120 V; skimmer: 65 V; Oct 1 RF Vpp: 750 V. Acquisition mode: MS. Acquisition rate: 5 Hz.

Three biological replicates of the empty plasmid pOSV801 exconjugants and the *sna* BGC exconjugants were analyzed in parallel. The Agilent data files were converted to mzML data formats using MSConvert.[5] Comparative metabolomics calculations were performed using XCMS[6] pairwise comparison with UPLC/UHD Q-TOF default parameters. New metabolites were predominantly in the medium supernatant. The metabolites were identified using either C18 or HILIC columns. However, the HILIC column offered better MS response and separation of the target metabolites from medium components and, therefore, was chosen for analysis.

Isolation of compounds 1, 2, and 3 for structural elucidation:

Eight microliters of spores of *S. albidoflavus sna* BGC exconjugants were inoculated into 50 mL of TSB medium in 250 mL flasks with stainless steel springs as baffles. This starter culture was incubated at 30 °C 220 rpm for 2 days. The starter culture was inoculated into 1 L of R5 medium in a 4 L baffled flask and was incubated at 30 °C and 220 rpm for another 4 days. The spent medium was harvested by centrifugation at 5000 g for 15 min and was further clarified by filtration against filter paper. A 10 g C18 SPE column (ThermoFisher 60108-703) hydrated with MeOH and equilibrated with H_2O was used to extract 150 mL of the spent medium. After washing with 2 CV of H_2O, bound metabolites were eluted using 2×10 mL 25% acetonitrile in H_2O, 2×10 mL 50% acetonitrile in H_2O, and 2×10 mL acetonitrile. The elution was concentrated by lyophilization and was further purified using a 10 mm × 250 mm Atlantis HILIC column with the following gradient:

Solvent A: H_2O + 10 mM ammonium formate, pH 3.2. Solvent B: 95% acetonitrile in H_2O + 10 mM ammonium formate, pH 3.2. Flow rate: 4 mL/ min.

100% B to 80% over 8 min; 80% B to 64% B over 14 min, 64% B to 60% B over 2 min, 60% B to 100% B over 1 min. The column was re-equilibrated at 100% B between injections for 5 min.

Metabolites from the *sna* BGC eluted from 18-24 min. The elutions were combined and lyophilized to dryness. The residual solid after lyophilization was resuspended in H_2O and was further purified on a 4.6 × 250 mm Vydac C18 column using the following gradient:

Solvent A: H_2O + 0.1% trifluoroacetic acid. Solvent B: acetonitrile + 0.1% trifluoroacetic acid

2% B to 20% over 18 min; 20% B to 60% B over 2 min; 60% B to 2% B over 1 min. The column was re-equilibrated at 2% B between injections for 4 min.

Compound **1** eluted at 12.4 min. Compound **2** eluted at 12.8 min. Compound **3** eluted at 13.2 min. The elution fraction for each compound was lyophilized to dryness for further characterization.

^{13}C labeling of the product of polyketide synthase SnaB

Stock solutions of 100 mg/mL $^{13}C_2$-sodium acetate and 2-^{13}C-sodium propionate were prepared in H_2O and were sterile-filtered. To an 18×150 mm culture tube were added 4 mL of R5 medium and 40 μL of 100 mg/mL $^{13}C_2$-sodium acetate or 2-^{13}C-sodium propionate. Three microliters of *S. albidoflavus* exconjugants spore suspension were inoculated into the expression medium. The culture supernatant was harvested after four days and purified as described above using a 100 mg C18 SPE column.

Determination of stereochemistry by Marfey's analysis[7, 8]

Compound **3** (0.1 μmol) was dissolved in 400 μL of 6 M DCl in D_2O. The solution was transferred to a 12×35 mm glass vial. The hydrolysis reaction was performed at 110 °C while stirring at 80 rpm for 3 h. The hydrolysate was concentrated using a SpeedVac vacuum concentrator (SPD140DDA) and lyophilized to dryness. The residue was redissolved in 200 μL of H_2O.

Derivatization with 1-fluoro-2-4-dinitrophenyl-5-L-alanine amide (L-FDAA) was performed by mixing 12.5 μL of 1 M $NaHCO_3$, 25 μL of redissolved concentrated hydrolysate, and 12.5 μL of 3 mg/mL L-FDAA dissolved in acetonitrile. The derivatization reaction was allowed to proceed at 42 °C for 20 min. The reaction solution was acidified with 1 μL of formic acid and centrifuged at 20,000 x*g* for 3 min before LC-MS analysis. A 0.5 mM solution of each amino acid standard was derivatized under the same conditions. As reported in previous studies, separation of the DL arginine pair[7] and the L-Thr/L-*allo*-Thr pair[9] is difficult. Indeed, we needed to screen different columns and mobile-phases to find conditions that allowed separation of the two arginine isomers and the four threonine isomers.

The arginine and threonine isomers were separated on a 2.1× 100 mm F5 column (Phenomenex 00D-4722-AN) maintained at 45 °C using the following gradient:

Solvent A: H_2O + 0.1% formic acid. Solvent B: MeOH. Flow rate: 0.4 mL/ min

5% B from 0-1 min, 5% B to 55% B over 7 min; 55% B to 95% B over 3 min; isocratic 95% B for 0.2 min. The column was re-equilibrated at 5% B between injections for 3 min.

For L-FDAA derivatized threonine, negative mode was used for detection and the [M-H] ion at m/z = 370.1004 was used in the extracted ion chromatogram (EIC). For L-FDAA derivatized arginine, positive mode was used for detection and the [M+H] ion at m/z = 427.1684 was used in the EIC.

Sequence analysis of ureido-generating condensation domains

The BGCs of ureido-containing NRPs were compiled from MiBiG or NCBI, including antipain (BGC0001570,[10] BGC0002051[11, 12]), anabaenopeptins (BGC0000301,[13] BGC0000302,[14]

BGC0001479,[15, 16] BGC0002512[17]), syringolin (BGC0000436,[18] BGC0001047[19]), pacidamycin (BGC0000951[20]), napsamycin (BGC0000950[21, 22]), muraymycin (BGC0001020[12, 23]), chitinimide (BGC0002503[24]), pseudovibriamide (BGC0002123[25]), and bulbiferamide (NCBI Bioproject PRJNA941849).[26, 27] The C domain sequences were extracted based on the domain annotation in MiBiG.[28]

By matching the adenylation domain specificity and the amino acids on each side of the ureido moiety of the final product, the C_{urea} domain were identified based on the canonical C-A-T module architecture. In modular NRPSs, the C_{urea} domain was always found in the first peptide extension module following the loading A-T didomain, as in the case of anabaenopeptins, chitinimide, pseudovibriamide, and bulbiferamide.

The C_{urea} domains in syringolin A,[29] pacidamycin,[20] antipain,[12] and muraymycin[12] have been confirmed by in vitro enzyme activity, which allowed the prediction of C_{urea} domains from similar BGCs such as napsamycin[21] and deimino-antipain.[10]

For phylogenetic analysis, the C domain sequences were aligned by MUSCLE. . A phylogenetic tree was calculated using the PhyML 3.0 server[30, 31] with smart model selection[32] using the Akaike Information Criterion and 100 bootstrap analysis for branch support values. The resulting tree was visualized using Interactive Tree of Life (iTOL).[33]

The amino acid translations of all genes in MiBiG were downloaded in a FASTA file (Version 3.1). The condensation domain sequences were filtered out by searching the database using the hidden Markov model of PF00668 with an E value threshold of 0.01. Examining all the extracted condensation domain sequences showed that the EHHXXHDG active site is confined to C_{urea} domains.

Reconstitution of SnaA enzymatic activity in vitro.

The DNA sequence of SnaC was amplified from pOSV801-SP44-Sna using the primer pair His-TEV-SnaC_F and His-TEV-SnaC_R (Table S1). The expression vector pRSFDuet was linearized by the primer pair SnaC_RSF_F/R. SnaC was cloned into the multiple cloning site II of pRSFDuet by Gibson assembly to yield the plasmid construct: pRSFDuet: empty: His_6-TEV-SnaC.

The DNA sequence encoding SnaA was codon-optimized and synthesized by Twist Biosciences in three fragments. The three synthetic gene fragments of *snaA* were amplified using the primer pairs coSnaA1_F/R, coSnaA2_F/R, and coSnaA3_F/R (Table S1). The expression vector pRSFDuet: empty: His_6-TEV-SnaC was linearized by the primer pair coSnaA_RSF_F/R. The DNA fragments of SnaA were inserted into the multiple cloning site I of pRSFDuet: empty: His_6-TEV-SnaC with a C-terminal Histag using Gibson assembly to yield the plasmid pRSFDuet: coSnaA-TEV-His_6: His_6-TEV-SnaC. The C_{urea} domain active site mutants E744A, H746A and H749A were generated using the NEB KLD method with the primer pairs coSnaAE744A_F/R, coSnaAH746A and coSnaAH749A_F/R (Table S1). The PCP active site mutant S556A and S1616A were generated with the primer pairs coSnaAS556A_F/R and coSnaAS1616A_F/R (Table S1).

The above plasmid containing SnaA and SnaC was used to transform *E. coli* BAP1 cells for protein expression. The transformants were selected on LB agar with 50 μg/mL kanamycin at 37 °C. A single colony was used to inoculate 7 mL of LB with 50 μg/mL kanamycin and was grown at 37 °C and 220 rpm for 4 h. The seed culture was used to inoculate 500 mL of LB with 50 μg/mL kanamycin, 1 mM $MgSO_4$, and trace metal mix (1X, Teknova T1001). The culture was grown at

37 °C and 220 rpm until OD_{600}= 0.6 when the shaker temperature was reduced to 16 °C. After 30 min of incubation at 16 °C, protein expression was induced by the addition of 0.2 mM final concentration of IPTG.

The cells were harvested after 16 h of incubation by centrifugation under 5000 g at 10 °C for 15 min and were resuspended in protein purification buffer containing 25 mM 3-(*N*-morpholino)propanesulfonic acid (MOPS), 150 mM KCl, 10% glycerol, pH 7.5. The cells were lysed by sonication under 40% maximal amplitude with 1 s on and 2 s off for 3 min. The lysate was clarified by centrifugation at 49990 g at 10 °C for 15 min. The imidazole concentration of the supernatant was adjusted to 20 mM, and the supernatant was incubated with 1 mL of His60 Ni Superflow Resin (Takara 635660) at 4 °C for 20 min. The resin was harvested by centrifugation under 2000 g for 3 min and was resuspended in the protein purification buffer supplied with 30 mM imidazole. The resin suspension was transferred to a gravity-flow column, and the resin was further washed with 10 mL each of protein purification buffer supplied with 30 mM, 50 mM, and 100 mM imidazole. After washing the resin with 2 mL of protein purification buffer supplied with 150 mM imidazole, bound protein was eluted with 1.5 mL each of protein purification buffer supplied with 200 mM, 250 mM, and 300 mM imidazole. The pooled elution fractions were concentrated and the buffer was exchanged to the protein purification buffer using an Amicon Ultra-4 Centrifugal Filter with 30 kDa molecular weight cutoff (MWCO). The protein solution containing SnaA and SnaC was flash-frozen with liquid nitrogen and was stored at -80 °C.

Ureido-peptide formation assay was performed in 50 μL of 50 mM sodium phosphate, 100 mM NaCl (pH 7.5) containing 5 mM $NaHCO_3$, 4 mM $MgCl_2$, 0.5 mM tris(2-carboxyethyl)phosphine (TCEP), 1 mM L-arginine, 4 mM ATP, 5 μM of SnaA-TEV-His_6 coexpressed with His_6-TEV-SnaC, and 40 mM cysteamine (final concentration). Two hours later, 25 μL of the reaction solution was taken out and quenched by 50 μL cold acetonitrile. The quenched reaction was centrifuged at 20000 g for 3 min to remove the protein precipitates.

A 25 μL sample of the quenched reaction supernatant was mixed with 12.5 μL of 200 mM sodium borate buffer (pH 10.4) and 12.5 μL of 15 mg/mL fluorenylmethyloxycarbonyl chloride (Fmoc-Cl) in acetonitrile. The reaction was allowed to proceed at room temperature for 30 min. The derivatization reaction was centrifuged at 20000 g for 3 min to remove the precipitates, and 1 μL of the reaction supernatant was used for LC-MS analysis with the following condition.

Column: Agilent 3×100 mm Poroshell C18 maintained at 45 °C

Solvent A: H_2O + 0.1% formic acid. Solvent B: acetonitrile + 0.1% formic acid

The gradient was 15% to 85% solvent B over 8 min, 85% B to 100% B over 1.71 min, isocratic 100% B over 1.29 min, 100% B to 5% B over 1 min. The column was re-equilibrated at 15% B between injections for 3 min.

NMR data of compound **1** (for actual spectra see Figures S14-S19):

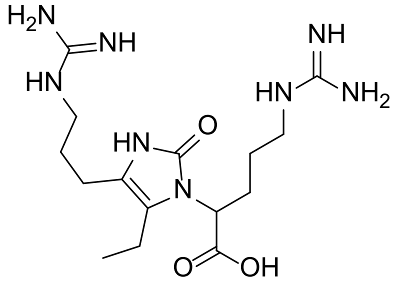

1H NMR (600 MHz, DMSO) δ 9.80 (s, 1H), 7.72 (br, 1H), 7.58 (br, 1H), 4.41 (t, *J* = 7.6 Hz, 1H), 3.14 – 3.04 (m, 4H), 2.36 – 2.22 (m, 4H), 2.04 (q, *J* = 8.3 Hz, 2H), 1.65 (p, *J* = 7.4 Hz, 2H), 1.47 – 1.41 (m, 1H), 1.32 – 1.26 (m, 1H), 0.97 (t, *J* = 7.4 Hz, 3H).

^{13}C NMR (151 MHz, DMSO) δ 171.9, 156.7, 153.1, 119.8, 114.6, 53.6, 40.4, 40.2, 27.9, 26.3, 25.7, 20.7, 15.6, 14.8.

NMR data of compound **2** (for actual spectra see Figures S20-S26):

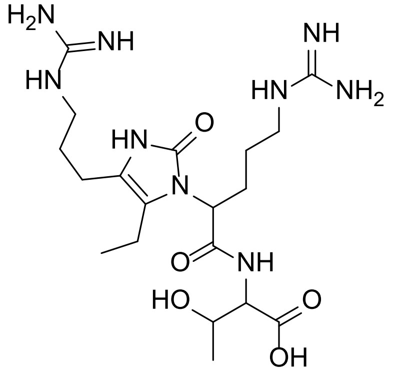

^{1}H NMR (500 MHz, DMSO) δ 10.01 (s, 1H), 7.73 – 7.70 (m, 2H), 7.59 (t, *J* = 5.8 Hz, 1H), 5.01 (s, 1H), 4.38 (t, *J* = 8.0 Hz, 1H), 4.17 – 4.13 (m, 2H), 3.13 – 3.05 (m, 4H), 2.38 – 2.27 (m, 4H), 2.14 – 2.06 (m, 2H), 1.65 (p, *J* = 7.7 Hz, 2H), 1.47 – 1.38 (m, 1H), 1.33 – 1.27 (m, 1H), 1.03 (d, *J* = 6.2 Hz, 3H), 0.98 (t, *J* = 7.4 Hz, 3H).

^{13}C NMR (126 MHz, DMSO) δ 171.8, 170.5, 156.8, 156.7, 153.6, 119.9, 115.5, 66.0, 57.6, 56.5, 40.3, 40.1, 27.8, 26.2, 25.7, 20.7, 20.4, 15.7, 14.7.

NMR data of compound **3** (for actual spectra see Figures S27-S32):

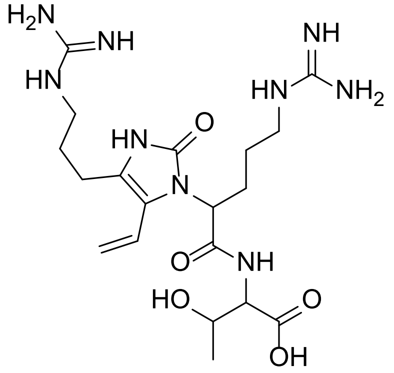

^{1}H NMR (600 MHz, DMSO) δ 10.45 (s, 1H), 7.64 (br, 1H), 7.60 (d, J = 8.5 Hz, 1H), 7.56 (br, 1H), 6.37 (dd, J = 17.7, 11.6 Hz, 1H), 5.15 (dd, 2H), 4.99 (d, J = 6.6 Hz, 1H), 4.78 (dd, J = 10.8, 5.1 Hz, 1H), 4.18 (dd, J = 8.5, 3.1 Hz, 1H), 4.14 (br, 1H), 3.13 – 3.04 (m, 4H), 2.47 – 2.41 (m, 2H), 2.10 – 2.06 (m, 1H), 1.89 (br, 1H), 1.70 (p, J = 7.2 Hz, 2H), 1.35 – 1.21 (m, 2H), 1.01 (d, 3H).

^{13}C NMR (151 MHz, DMSO) δ 171.8, 170.5, 156.6, 153.4, 124.1, 120.1, 117.5, 114.4, 66.1, 58.0, 55.0, 40.1, 40.1, 27.7, 26.6, 25.4, 21.5, 20.5.

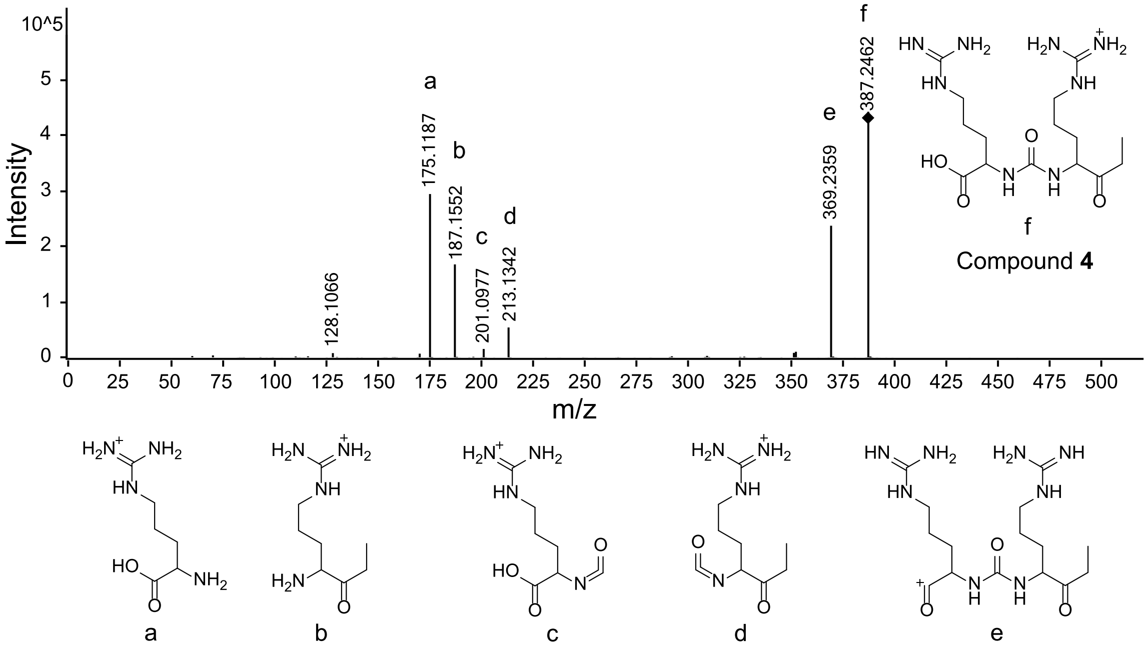

ion	experimental m/z	theoretical m/z	ppm-error
a	175.1187	175.1190	-1.71
b	187.1552	187.1553	-0.53
c	201.0977	201.0982	-2.49
d	213.1342	213.1346	-1.88
e	369.2359	369.2357	0.54
f	387.2462	387.2463	-0.26

Figure S1. High-resolution tandem mass spectrometry (MS/MS) of compound **4** using 10 eV collision energy. The assignment of selected fragment ions and their ppm errors compared to calculated m/z values are listed.

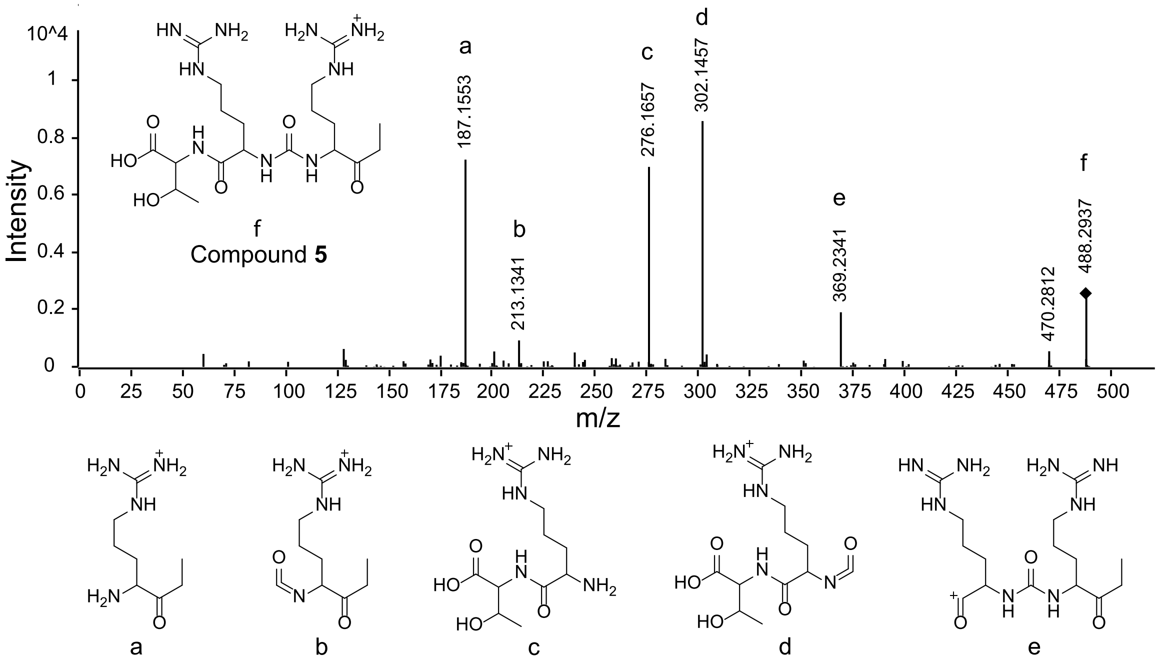

ion	experimental m/z	theoretical m/z	ppm-error
a	187.1553	187.1553	0.00
b	213.1341	213.1346	-2.35
c	276.1657	276.1666	-3.26
d	302.1457	302.1459	-0.66
e	369.2341	369.2357	-4.33
f	488.2937	488.2940	-0.61

Figure S2. High-resolution tandem mass spectrometry (MS/MS) spectrum of compound **5** using 20 eV collision energy. The assignment of selected fragment ions and their ppm errors compared to calculated m/z values are listed.

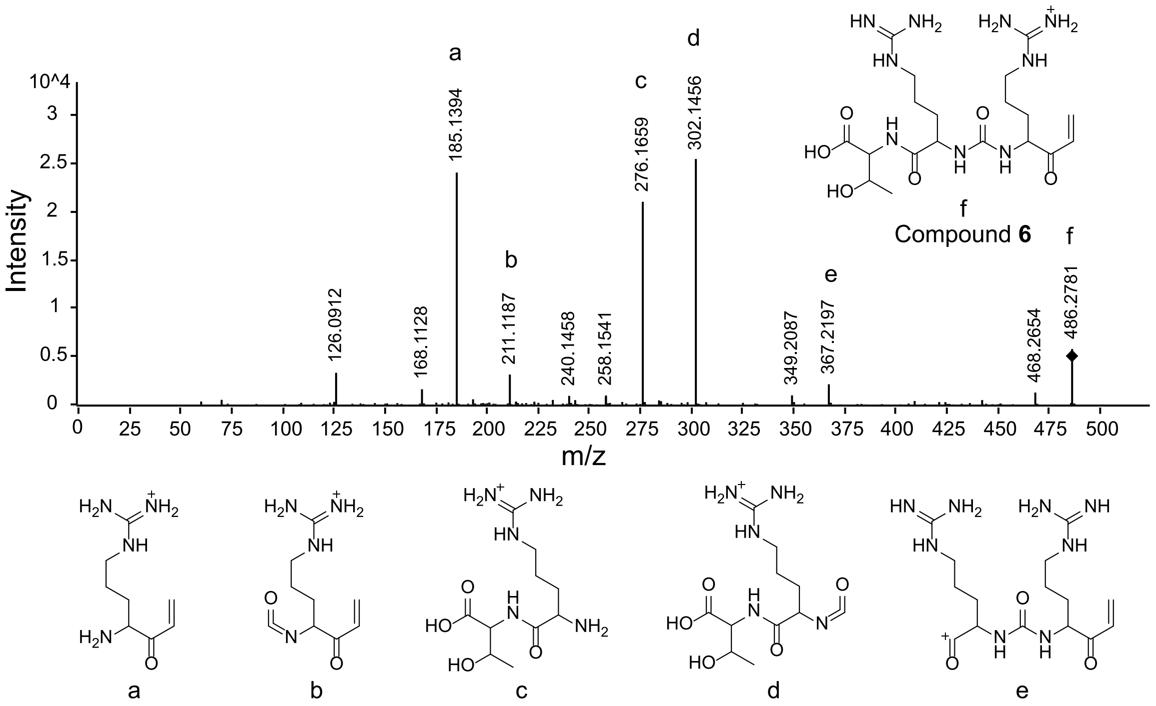

ion	experimental m/z	theoretical m/z	ppm-error
a	185.1394	185.1397	-1.62
b	211.1187	211.1190	-1.42
c	276.1659	276.1666	-2.53
d	302.1456	302.1459	-0.99
e	367.2197	367.2201	-1.09
f	486.2781	486.2783	-0.41

Figure S3. High-resolution tandem mass spectrometry (MS/MS) spectrum of compound **6** using 20 eV collision energy. The assignment of selected fragment ions and their ppm errors compared to calculated m/z values are listed.

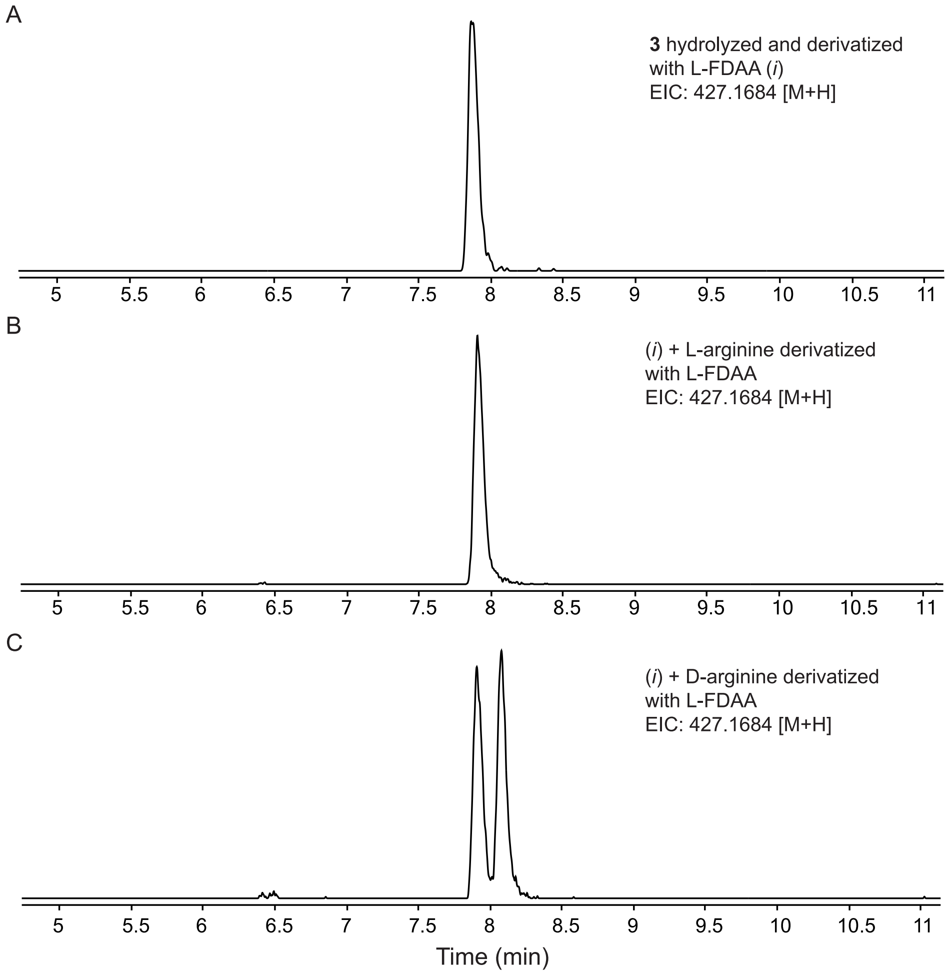

Figure S4. Stereochemical analysis of the arginine in **3** using Marfey's analysis. (A) The EIC of L-FDAA-derivatized arginine after hydrolysis and derivatization of **3**. (B). L-arginine was derivatized under the same conditions and co-injected with the derivatized material of **3**. The coelution of L-FDAA-derivatized arginine in **3** and L-arginine shows the configuration of the arginine in **3** is L. (C) D-arginine was derivatized under the same conditions and co-injected with the derivatized material of **3**. L-FDAA-derivatized arginine in **3** and D-arginine did not coelute, which confirms the configuration of arginine in **3** is not D. The y-axis was scaled for clarity.

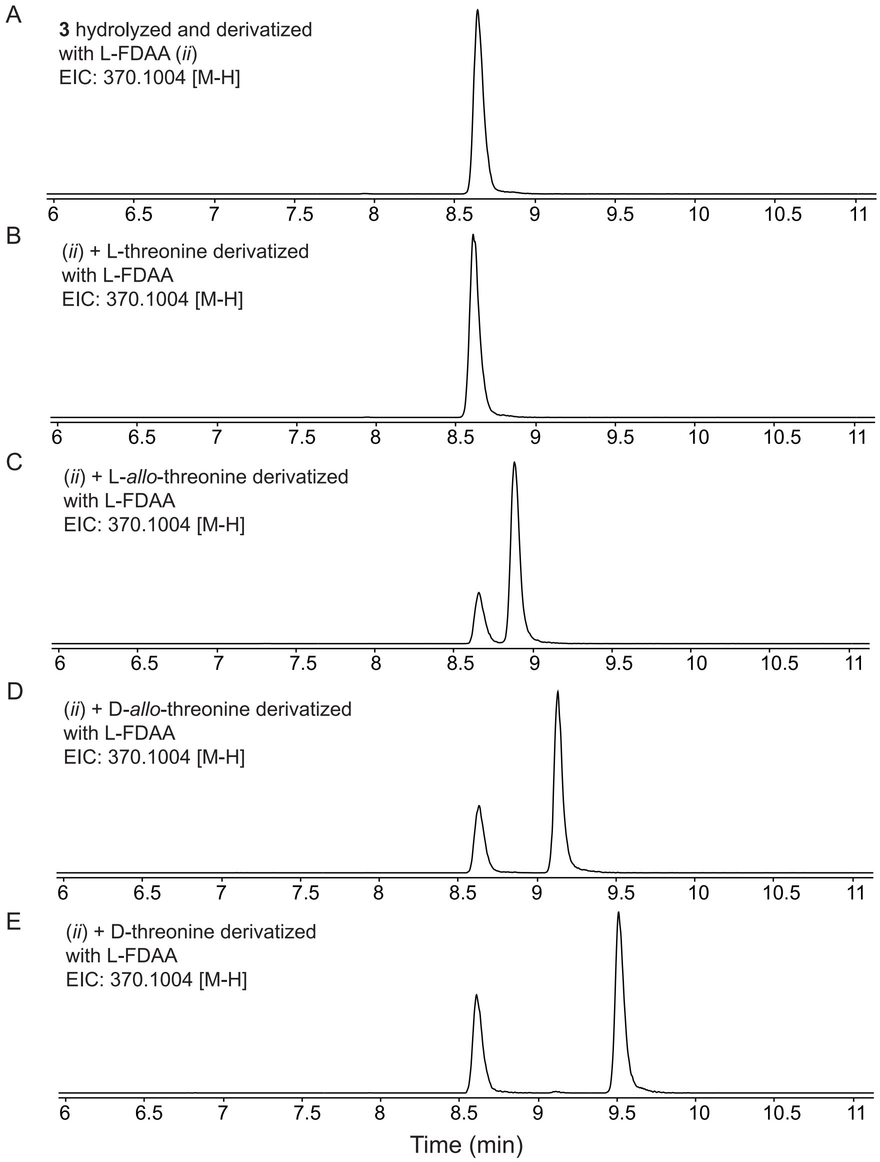

Figure S5. Stereochemical analysis of threonine in **3** using Marfey's analysis. (A) EIC of L-FDAA-derivatized threonine after hydrolysis and derivatization of **3**. L-threonine (B), L-*allo*-threonine (C), D-*allo*-threonine (D), and D-threonine (E) was derivatized under the same conditions and co-injected with the derivatized material of **3**. L-FDAA-derivatized threonine in **3** only coeluted with L-FDAA-derivatized L-threonine, which indicates the threonine configuration in **3** is L.

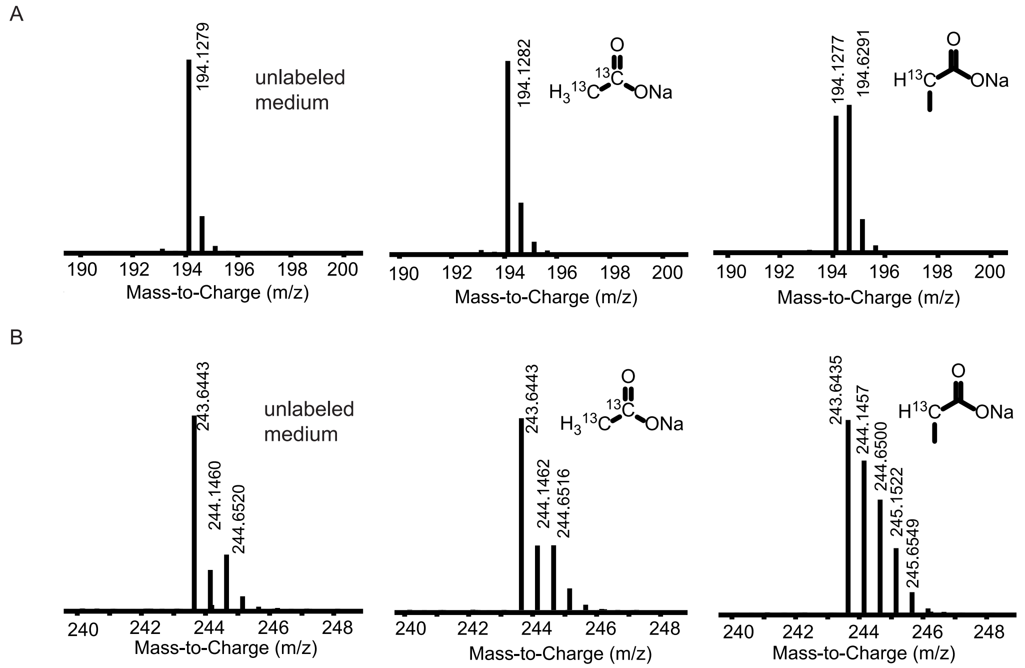

Figure S6. Comparison of MS spectra of compounds **4**, **5**, and **6** when different PKS precursors were fed. (*A*) MS spectra of compound **4** when the medium was supplied with $^{13}C_2$-sodium acetate or 2-^{13}C-sodium propionate. Only significant isotope incorporation was observed when the medium was supplied with 2-^{13}C-sodium propionate. These data are consistent with conversion of propionate to methylmalonyl-CoA, and subsequent utilization by SnaB. (*B*) Mass spectra of compounds **5** and **6** when the medium was supplied with $^{13}C_2$-sodium acetate or 2-^{13}C-sodium propionate. Only significant isotope incorporation was observed when the medium was supplied with 2-^{13}C-sodium propionate. Compound **5** (244.6506 m/z, [M+2H]) and **6** (243.6428 m/z, [M+2H]) coelute under the HILIC-MS condition, which explains the unusual isotope distribution of the spectra.

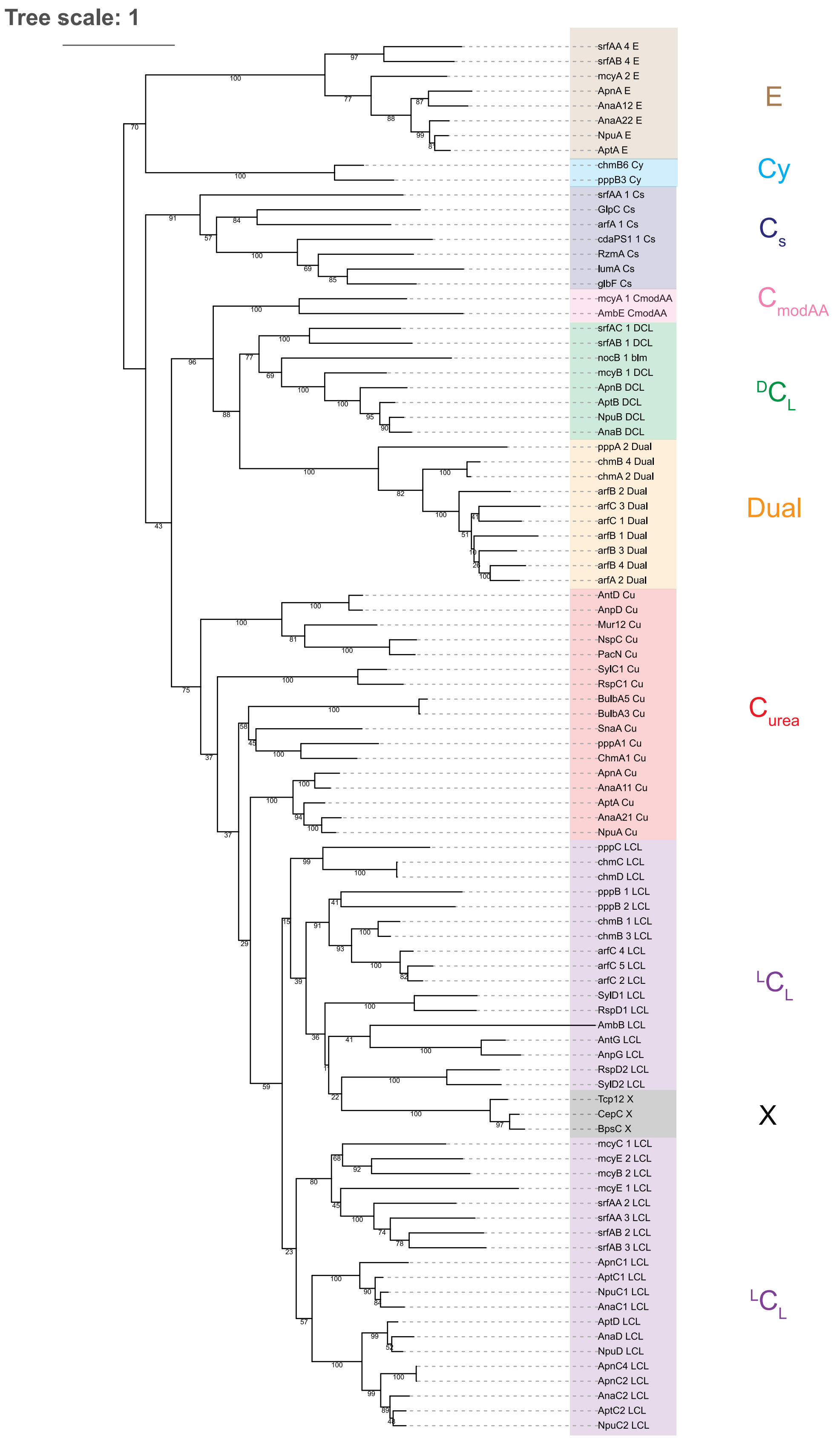

Tree scale: 1
srfAA 4 E
srfAB 4 E
mcyA 2 E
ApnA E
AnaA12 E
AnaA22 E
NpuA E
AptA E
chmB6 Cy
pppB3 Cy
srfAA 1 Cs
GlpC Cs
arfA 1 Cs
cdaPS1 1 Cs
RzmA Cs
lumA Cs
glbF Cs
mcyA 1 CmodAA
AmbE CmodAA
srfAC 1 DCL
srfAB 1 DCL
nocB 1 blm
mcyB 1 DCL
ApnB DCL
AptB DCL
NpuB DCL
AnaB DCL
pppA 2 Dual
chmB 4 Dual
chmA 2 Dual
arfB 2 Dual
arfC 3 Dual
arfC 1 Dual
arfB 1 Dual
arfB 3 Dual
arfB 4 Dual
arfA 2 Dual
AntD Cu
AnpD Cu
Mur12 Cu
NspC Cu
PacN Cu
SylC1 Cu
RspC1 Cu
BulbA5 Cu
BulbA3 Cu
SnaA Cu
pppA1 Cu
ChmA1 Cu
ApnA Cu
AnaA11 Cu
AptA Cu
AnaA21 Cu
NpuA Cu
pppC LCL
chmC LCL
chmD LCL
pppB 1 LCL
pppB 2 LCL
chmB 1 LCL
chmB 3 LCL
arfC 4 LCL
arfC 5 LCL
arfC 2 LCL
SylD1 LCL
RspD1 LCL
AmbB LCL
AntG LCL
AnpG LCL
RspD2 LCL
SylD2 LCL
Tcp12 X
CepC X
BpsC X
mcyC 1 LCL
mcyE 2 LCL
mcyB 2 LCL
mcyE 1 LCL
srfAA 2 LCL
srfAA 3 LCL
srfAB 2 LCL
srfAB 3 LCL
ApnC1 LCL
AptC1 LCL
NpuC1 LCL
AnaC1 LCL
AptD LCL
AnaD LCL
NpuD LCL
ApnC4 LCL
ApnC2 LCL
AnaC2 LCL
AptC2 LCL
NpuC2 LCL
E
Cy
Cs
CmodAA
DCL
Dual
Curea
LCL
X
LCL

Figure S7. Unrooted phylogenetic tree of C domains associated with ureido-containing nonribosomal peptides and select other characterized BGCs with support values from 100 bootstrap replicates. The rectangular view was chosen to better show the members of each type of C domain. The C domains group according to their catalytic functions rather than the origin of BGCs or their amino acid specificities. The ureido-generating C domains (C_{urea}) are highlighted in red. E: epimerization domains. Cy: cyclization domains. Cs: starter C domains. C_{modAA}: modifying amino acid C domains. $^{D}C_{L}$: condensation between D-aminoacyl donor and L-aminoacyl acceptor. Dual: Dual condensation and epimerization domains. $^{L}C_{L}$: condensation between L-aminoacyl donor and acceptor. X: X-domain that recruits P_{450} in glycopeptide antibiotics biosynthesis. The multiple sequence alignment used to compute the tree and the tree file are available in Dataset S1-S2.

A

B

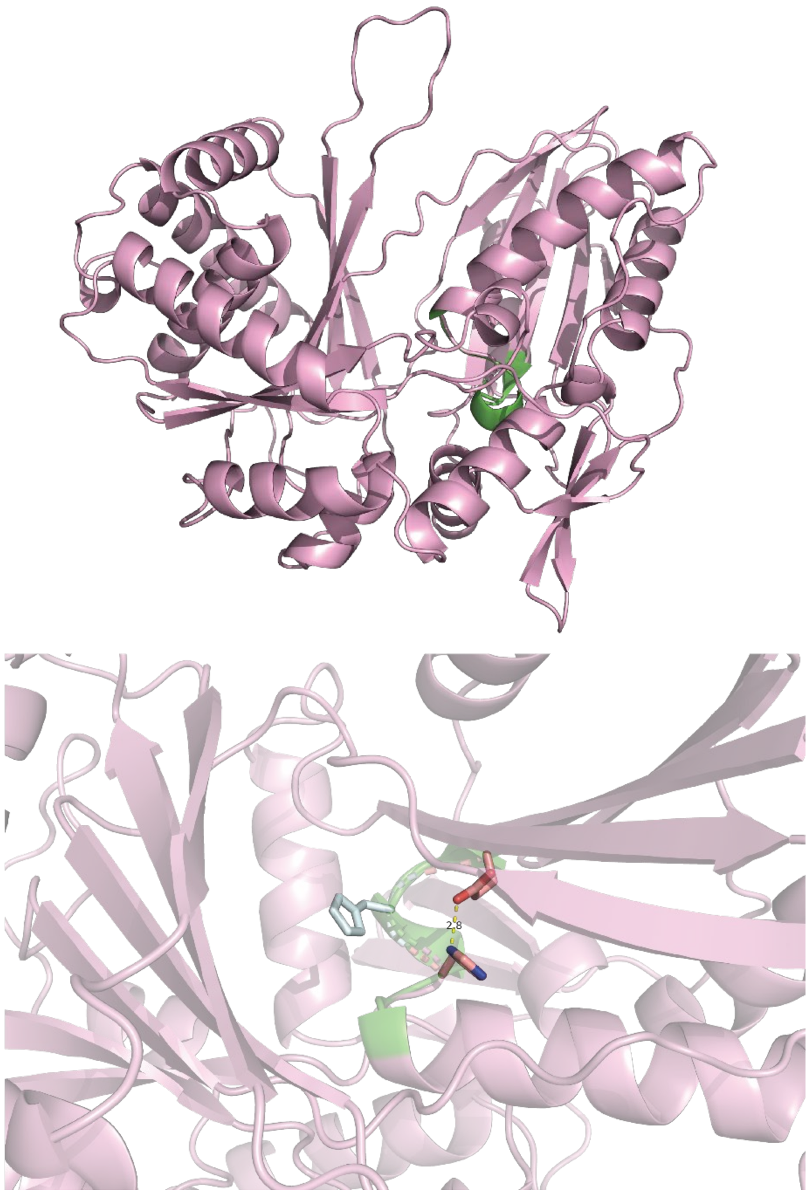

Figure S8. (*A*) AlphaFold model of C_{urea} domain of SnaA. The region of the active site motif (EHHXXHDG) is colored green. (*B*) Zoom-in of the active site residues. H746 is colored cyan. The conserved E744 and H749 in C_{urea} domains are shown in pink. A salt bridge interaction is predicted by AlphaFold.

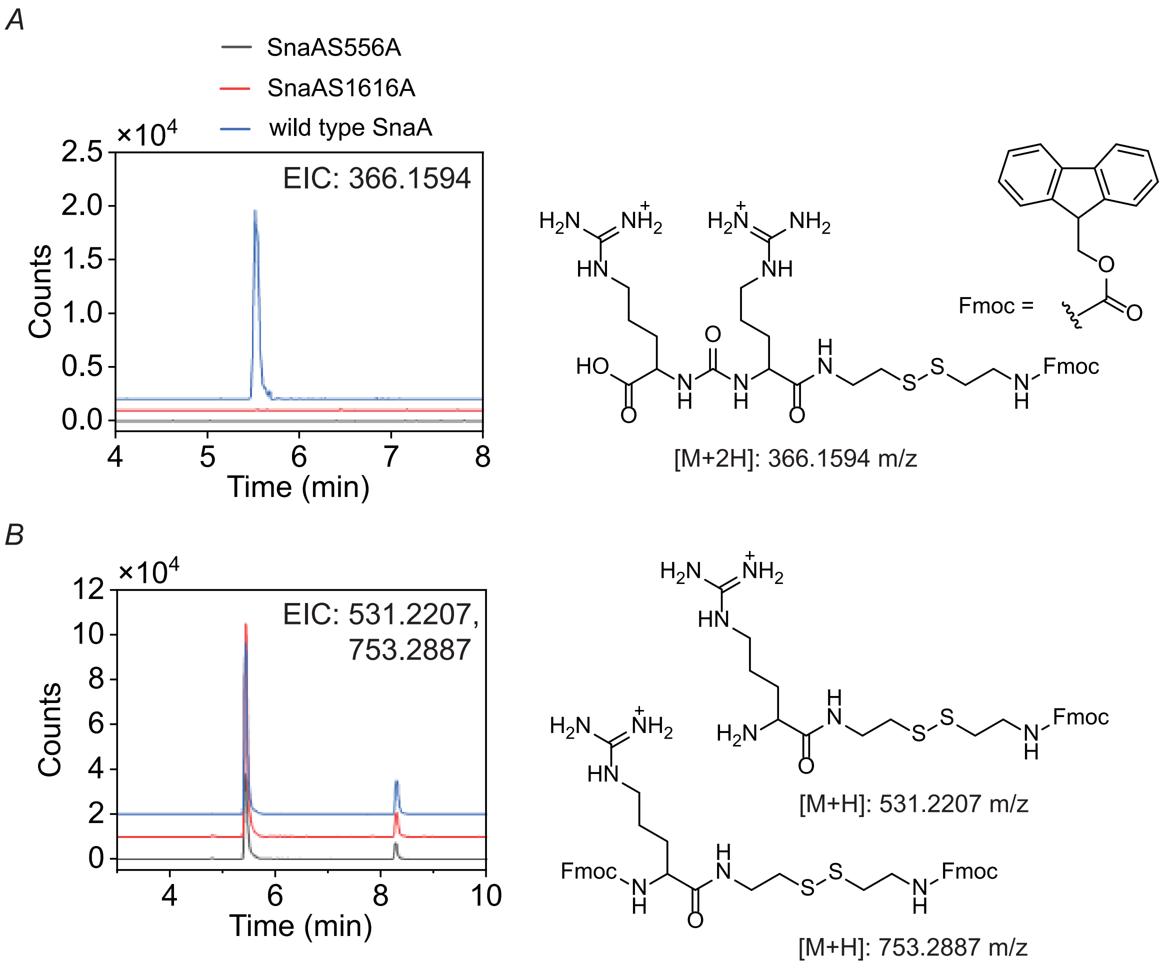

Figure S9. Enzymatic activity of individual PCP mutants of SnaA. (*A*) Detection of cysteamine-intercepted and Fmoc-derivatized and bisarginyl ureido dipeptide in vitro. Inactivation of each of the two PCP domains by substitution of the conserved active site serine to alanine abolished the generation of ureido dipeptide, indicating both PCP domains are needed for the ureido-generating process. A space of 1000 counts was used to offset each EIC trace for clarity. (*B*) Detection of cysteamine-intercepted and Fmoc-derivatized arginine. Mono- and di-derivatization was observed, and therefore a combined EIC of the two species is shown. The mono-derivatized species has a retention time of 5.4 min and the di-derivatized species has a retention time of 8.3 min. When one PCP was inactivated, the other PCP was still loaded with arginine. A space of 10^4 counts was used to offset each EIC trace for clarity. SnaC was present in all in vitro assays.

A.

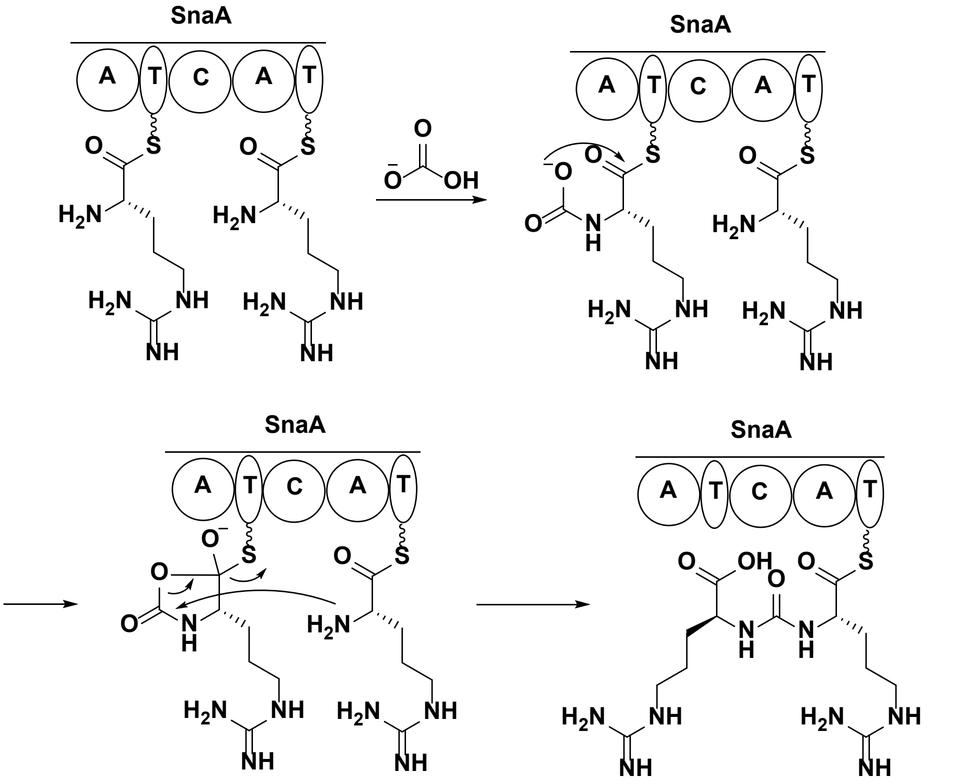

B.

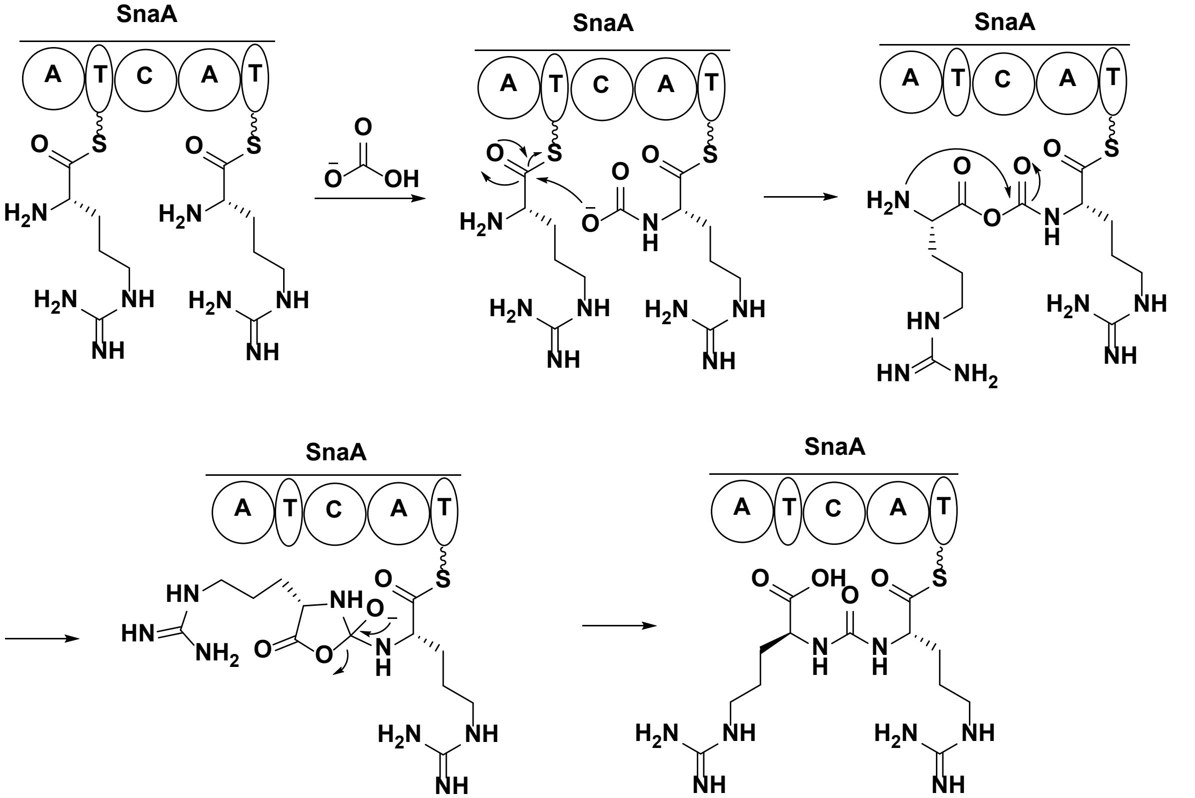

C.

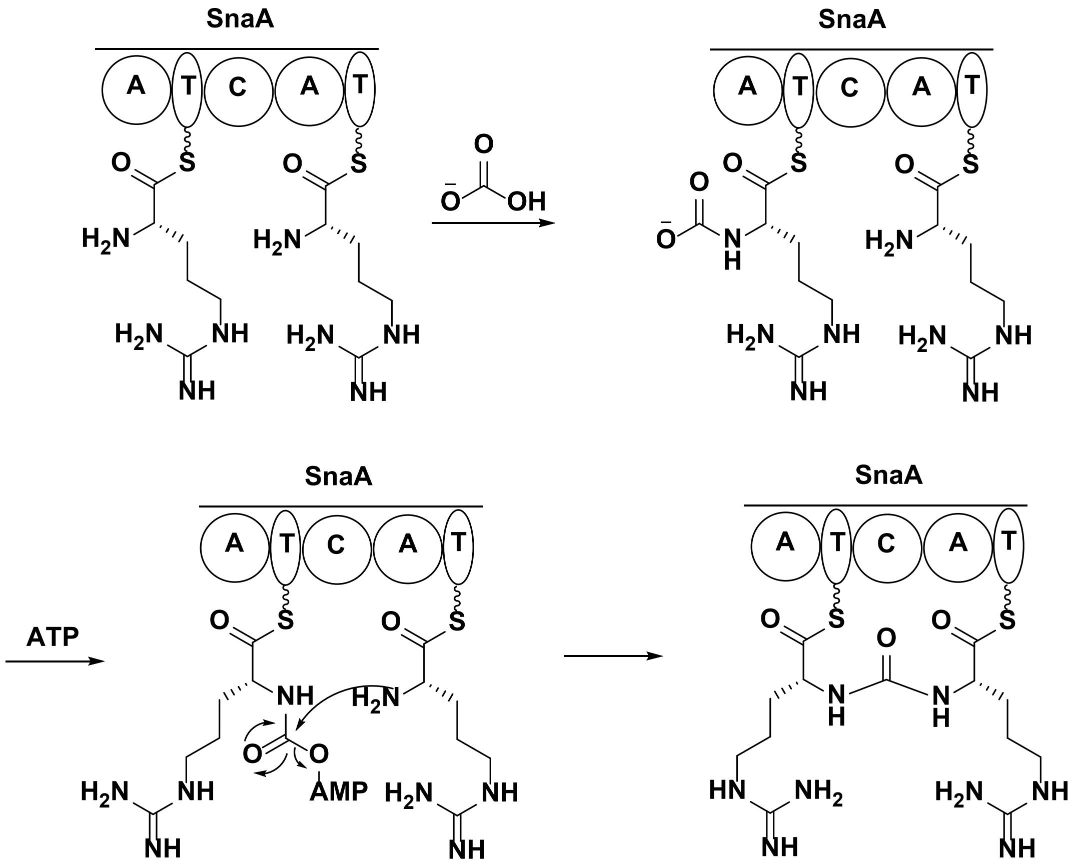

Figure S10. Three proposed mechanisms for ureido group formation catalyzed by SnaA. The mechanisms in panels A and B utilize the thioester linkage to activate the initially formed carbamate whereas the mechanism in panel C uses ATP for carbamate activation (shown as adenylation).

We note that the mechanism in Panel C in principle could have a peptide bound to the first T-domain (from chain extension with a previous module) and therefore could be an alternative means to use of a PEARL to form a ureido containing product with an extended chain on both sides of the ureido moiety.

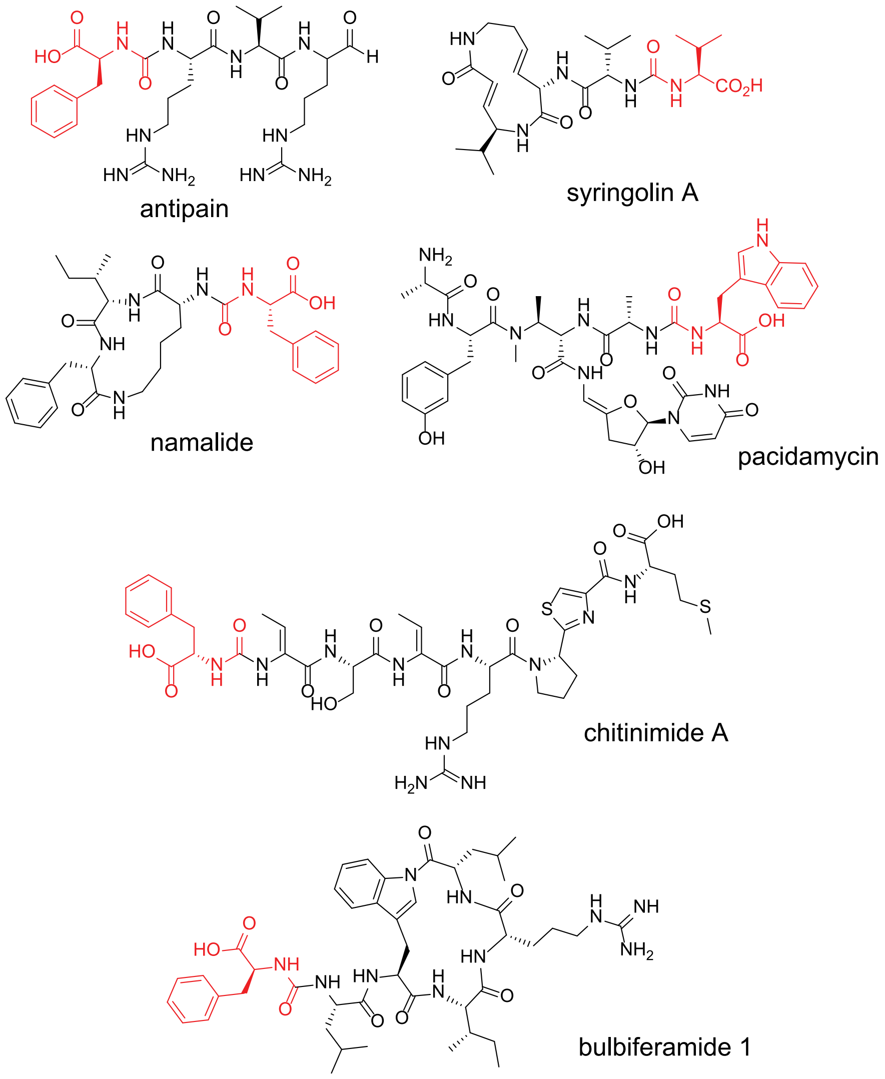

Figure S11. Structures of selected examples of ureido-containing nonribosomal peptides. The terminal amino acids of the ureido group have been highlighted in red. In all cases, there is only one amino acid at the terminal position of the ureido group.

```
NisB      NKVFLEQLLLANPKLYDV-MQKY---NAGLLKKKRVKKLFESIYKYYKRSYLRSTPFGLF  94
MibB      DGPLMEAVELASPSLAGL-LARVARGDTGGLKDKRLRRAALALLRYDIRMRTRPTPFGLF  146
SpaB      DSLFREQILVSSRTLYET-IHTFLQ-APDKLKGKKKRNFQQAILKYATRRATRTTPFGLF  97
EpiB      NDIFKESIMTTTYNLYQS-IGK----IDWEKDNKKTRNVKESLLKYLIRMSTRSTPYGML  94
TbtB      DPGFRRALSLASPELAAD-LDRW---LAEPARRPKTQKLLR-LAKYVARAAVKTSPYSTF  208
TglB      DEDVEQAVFISNPTALTR-L-RELRQERHARTDSRKKQKLRLAWSYAQRFCSKNDTSSFF  184
BhaB1     SSQYELALYTLNHKLWHF-WIKENR--LENLTDAQKKQTCRTLFAYLQRVSTKNDTIGEY  196
BhaB7     NPRLQEAIFQQSPSMYKNAVVPYVH-SSLQKRNTNIKRIERQLISYLQRLCTKNETTSFF  219
BhaB5     SEDFRQAVFISNPDMYQH-IDRYMKHFQSHSRPSKVKRIEKKLFTYLQRFCGKNESASFF  134
AmmB2     DPRFLEAVACSSPPAYRD--------LRKGARGARLRRQAAS---YAQRFAAKCETMSFF  170
SnaE      LPGFREAVAWQNPAILHN-WLTKMA-HSGDDRKLRKNRYMKAVASYAQRYFTKNDTIGFF  131
          .. :..::  ..: ...     .          ...:.:.  . . .*..* ..:....:.:

NisB      LDDLESHYQ----------------------------------KMDL----EKKSELQGL  432
MibB      IAELAYDEA-------------------------------APADLPN----SLELGVHVV  508
SpaB      LDDETFHRI-------------------------------CNSEIAD----EEIP-LSFE  439
EpiB      LEKNNTVSK-------------------------------INAPVST----EIYSEIYFG  434
TbtB      VDPEELIKQ----------------------------------MATW-------PEWIVV  557
TglB      LNTRLDVRT-------------------------------FSVFGSH----FHSPDFLIS  546
BhaB1     IN-----------------------------------------SFRW----LLSPDLMVK  453
BhaB7     LDYQDFDRD-----------------------------------MAW----LTSPDLMIA  562
BhaB5     LTSDQLNV--------------------------------FPSNRSF----YSLPDLFLQ  486
AmmB2     LDLTGRLVP-------------------------------PPPSGPP---VLCSVDVMIQ  647
SnaE      LADGETASGHRPDEVAIGEARAPRRQFASRDLVERAARLFPPAPAPWAGAMHLSPDIMIA  564
          :.                                           .        .....
```

NisB	ED--ENIINKGEK---GRVA**D**VVVPFIRTRALGNEGRAFIREKRVSVERREKLPFNE	736
MibB	AGDEEGWLDRGDAGFPGHLA**E**IVVPLERRDRHAARPPHIRATVSGREPTGAGGP---	818
SpaB	EHDADALMDRNQN---DYSG**E**IVVPLLRKQPEKPLYLPVLNAIEGSGSDRIKMPFED	755
EpiB	----ESFINESNN---ERML**E**IVTPLYKKTSLKEQSFIIPKNRNKHFNNLKD-----	723
TbtB	PDPADAPPREGSD--LPRVI**E**FLVELGE----------------------------	858
TglB	PAPDQLWLEETRG---HFCC**E**I**R**TTFRDNGVTRDE---------------------	815
$BhaB_1$	PGVEDLFLKDDRG---HYCC**E**L**R**TFAYRQEITSKDMLMG-----------------	731
$BhaB_7$	PNLSELWLSKEDQ--KTHTA**E**I**R**LSYFVERS-------------------------	823
$BhaB_5$	PDPHHLWLKSRKG---SHSC**E**L**R**MSVYKLGIKEVSEHA------------------	763
$AmmB_2$	PDRADSWLRHGGQ---PVAA**E**L**R**CVYLRRAA-------------------------	913
SnaE	PDLHQAWLPGPDG--EMYTS**E**L**R**FVAVNPHGTDNILDTPS----------------	845
	.. ...:..... . .:..... .	

Figure S12. Multiple sequence alignment of selected regions of SnaE and representative members of lantibiotic dehydratases (NisB,[34] MibB,[35] SpaB,[36] EpiB,[37] and TbtB[38]) and PEARLs (TglB,[39] BhaB$_1$,[40] BhaB$_7$,[40] BhaB$_5$,[40] and AmmB$_2$[41]). Conserved residues colored red are proposed to bind the 5'-phosphate of the aminoacyl-tRNA based on the crystal structure of TbtB (PDB 6EC8). These residues in red have been shown to be critical for the activity of NisB[34] (dehydratase), TbtB[38] (dehydratase), and TglB[39] (PEARL). PEARLs and SnaE possess an extra set of conserved residues colored blue that has been shown to be critical for the ATP-dependent phosphorylation activity of TglB to form an acylphosphate on the peptide substrate.[39] Among the three conserved residues for phosphorylation activity of PEARLs, only the glutamate colored purple (TbtB E851, NisB D700) is present in the dehydratases.[42]

Table S1. Primers used in this study.

Name	Sequence
Sna1F	ATGGATCAAAACAACACCGACAAC
Sna1R	GATGACGGCTATGTGGGTC
Sna2F	CAAAGACGATCCAGTGAGGAG
Sna2R	ACAACTTCCAGTCCGAGC
Sna3F	GAACTCCTGAAGGGAGTGTC
Sna3R	TCTCAGTTCTTCGAGTCGC
Sna_OSVF	GTGGCACAGCTGGCGCGGCGACTCGAAGAACTGAGActatgttgaccgc aaactggatg
Sna_OSVR	TCGTTCGCGGAGTTGTCGGTGTTGTTTTGATCCATatgggtgaaccgatctc ctc
SnaA$_{in}$_1F	CTCTAGAAAGTATAGGAACTTCATGCATGC
SnaA$_{in}$_1R	ATGGTGGTGGCGGTCAGGGCATGTCCACCCAGGAGGAAC
SnaA$_{in}$_2F	GTTCCTCCTGGGTGGACATGCCCTGACCGCCACCACCATCGC
SnaA$_{in}$_2R	CAGCAGCAGGGCATGCCCGCCGAGGTCGAAG
SnaA$_{in}$_3F	CGGGCATGCCCTGCTGCTGGGGCATC
SnaA$_{in}$_3R	GTTCGATCTCGGTGAGTTCCAG
SnaB$_{in}$_1F	GCCAAGATCGGTTTCACCG
SnaB$_{in}$_1R	CTGGGTCGCCAGCAGGGCGTCGCCGCCCAGCTTG
SnaB$_{in}$_2F	CAAGCTGGGCGGCGACGCCCTGCTGGCGACCCAGTTG
SnaB$_{in}$_2R	GAAGACCCGCAGTGGCTG
SnaE$_{in}$_1F	CGATCGTTCGCGAGGCCCTGGGCCTCAGCGAGATGCTGCCC
SnaE$_{in}$_1R	CTGATCCGATTGGCACGGCGGAC
SnaE$_{in}$_2F	GGACGAGCGTCTGCTCCGCCATTC
SnaE$_{in}$_2R	GCAGGGTGTCGATCCAGGCGAAG
SnaE$_{in}$_3F	CGATCGGCTGCCGAGCTACATGG
SnaE$_{in}$_3R	GGCAGCATCTCGCTGAGGCCCAGGGCCTCGCGAACGATCGC
SnaO$_{in}$_1F	GCCAAGATCGGTTTCACCG
SnaO$_{in}$_1R	GTAGGTGTTGGCCAGGTCGTTCAGTTCGCCGGTGAC
SnaO$_{in}$_2F	GTCACCGGCGAACTGAACGACCTGGCCAACACCTACTG
SnaO$_{in}$_2R	GAAGACCCGCAGTGGCTG
His-TEV-SnaC_F	GGCAGCAGCCATCACCATCATCACCACGAGAACCTGTACTTCCAAA GCTTCGAAGACAATGACGGCC
His-TEV-SnaC_R	CCGATATCCAATTGAGATCTGCTCAAACATGGGCATCGGTC
SnaC_RSF_F	CATGTTTGAGCAGATCTCAATTGGATATCGG
SnaC_RSF_R	GGTGATGATGGTGATGGCTGCTGCCCATATGTATATCTCCTTCTTATA CTTAACTAATATACTAAGATGG
coSnaA1_F	CTTTAATAAGGAGATATACCATGGACCAGAATAACACCGAC
coSnaA1_R	CTCGTCTGTACGAATTCTTGCC

coSnaA2_F	ACAATTGCGGCAAGAATTCG
coSnaA2_R	AACTACTCCGCTATCAGGTAAGC
coSnaA3_F	GCAGGACGCTTACCTGATAG
coSnaA3_R	CCGGACCCGCTTTGGAAGTACAGGTTCTCCTGAGATTCTTCGCTGGACC
coSnaA_RSF_F	GTACTTCCAAAGCGGGTCCG
coSnaA_RSF_R	GGTGTTATTCTGGTCCATGGTATATCTCCTTATTAAAGTTAAACAAAATTATTTCTAC
coSnaAE744A_F	GCGCATCATCTGGTACATGATGGTTGG
coSnaAE744A_R	AACAAAGATTAACTCGTGCTCG
coSnaAH746A_F	GCGCTGGTACATGATGGTTGGTCTTC
coSnaAH746A_R	ATGTTCAACAAAGATTAACTCGTGCTC
coSnaAH749A_F	GCGGATGGTTGGTCTTCTTCCCTC
coSnaAH749A_R	TACCAGATGATGTTCAACAAAGATTAAC
coSnaAS556A_F	GCGCTGACGGCCACTACAATTGC
coSnaAS556A_R	ATGACCACCCAGTAAAAACAGATC
coSnaAS1616A_F	CTGCTTTTGGGCCACCTGCACCACCGCATGAC
coSnaAS1616A_R	CGCGTGTCCACCCAGATCAAAAAAATTGTCACCCACAC

Table S2. Accession IDs for proteins in the *sna* BGC

SnaA	ADD43706.1
SnaB	ADD43707.1
SnaC	ADD43708.1
SnaD	ADD43709.1
SnaO	ADD43710.1
SnaE	ADD43711.1
$SnaT_1$	ADD43712.1
$SnaT_2$	ADD43713.1

Table S3. Accession IDs for proteins used to generate the C domain multiple sequence alignment and phylogenetic tree.

Protein	C domain subtype	MiBiG ID	NCBI ID
PacN	C_{urea}	BGC0000951	ADN26250.1
NpsC		BGC0000950	ADY76666.1
Mur12		BGC0001020	ADZ45324.1
AnpD		BGC0001570	OAL11435.1
AntD		BGC0002051	QOE83923.1
RspC		BGC0000436	EJK79842.1
SylC		BGC0001047	CAD70194.1
BulbA3		N.A.	WHI50395.1
BulbA5		N.A.	WHI48530.1

PppA1		BGC0002123	QUS58939.1
ChmA1		BGC0002503	QPB41096.1
SnaA		N.A.	ADD43706.1
ApnA		BGC0000301	ABV79985.1
AnaA11		BGC0000302	ACZ55945.1
AptA		BGC0002512	QNL14922.1
AnaA21		BGC0000302	ACZ55942.1
NpuA		BGC0001479	ACC81021.1
SrfAA4	E	BGC0000433	CAE02630.1
SrfAB4		BGC0000433	CAE02631.1
McyA2		BGC0001017	AAF00960.1
ApnAE		BGC0000301	ABV79985.1
AnaA12		BGC0000302	ACZ55945.1
AnaA22		BGC0000302	ACZ55942.1
NpuAE		BGC0001479	ACC81021.1
AptAE		BGC0002512	QNL14922.1
ChmB6	Cy	BGC0002503	QPB41097.1
PppB3		BGC0002123	QUS58938.1
SurfAA1	C_s	BGC0000433	CAE02630.1
GlpC		BGC0001608	AKJ29410.1
ArfA1		BGC0000305	BAC67534.2
cdaPS1		BGC0000315	CAB38518.1
RzmA		BGC0001758	WP_013428324.1
LumA		BGC0000383	AXG47007.1
GlbF		BGC0000997	CAL80824.1
McyA1	C_{modAA}	BGC0001017	AAF00960.1
AmbE		BGC0000287	AAG05690.1
SrfAC1	$^{D}C_{L}$	BGC0000433	CAE02633.1
SrfAB1		BGC0000433	CAE02631.1
McyB1		BGC0001017	AAF00961.1
ApnB		BGC0000301	ABV79986.1
AptB		BGC0002512	QNL14921.1
NpuB		BGC0001479	ACC81022.1
AnaB		BGC0000302	ACZ55943.1
NocB5	β-lactam formation	BGC0000395	AAT09805.1
PppA2	Dual	BGC0002123	QUS58939.1
ChmB2		BGC0002503	QPB41097.1
ChmA2		BGC0002503	BGC0002503
ArfB2		BGC0000305	BAC67535.1
ArfC3		BGC0000305	BAC67536.1
ArfC1		BGC0000305	BAC67536.1
ArfB1		BGC0000305	BAC67535.1
ArfB3		BGC0000305	BAC67535.1
ArfB4		BGC0000305	BAC67535.1
ArfA2		BGC0000305	BAC67534.2
PppC	$^{L}C_{L}$	BGC0002123	QUS58937.1
ChmC		BGC0002503	QPB41098.1
ChmD		BGC0002503	QPB41099.1

PppB1		BGC0002123	QUS58938.1
PppB2		BGC0002123	QUS58938.1
ChmB1		BGC0002503	QPB41097.1
ChmB3		BGC0002503	QPB41097.1
ArfC4		BGC0000305	BAC67536.1
ArfC5		BGC0000305	BAC67536.1
ArfC2		BGC0000305	BAC67536.1
SylD1		BGC0001047	CAD70195.1
RspD1		BGC0000436	EJK79843.1
AmbB		BGC0000287	AAG05693.1
AntG		BGC0002051	QOE83925.1
AnpG		BGC0001570	OAL11476.1
RspD2		BGC0000436	EJK79843.1
SylD2		BGC0001047	CAD70195.1
McyC1		BGC0001017	AAF00962.1
McyE2		BGC0001017	AAF00958.1
McyB2		BGC0001017	AAF00961.1
McyE1		BGC0001017	AAF00958.1
SrfAA2		BGC0000433	CAE02630.1
SrfAA3		BGC0000433	CAE02630.1
SrfAB2		BGC0000433	CAE02631.1
SrfAB3		BGC0000433	CAE02631.1
ApnC1		BGC0000301	ABV79987.1
AptC1		BGC0002512	QNL14923.1
NpuC1		BGC0001479	ACC81023.1
AnaC1		BGC0000302	ACZ55944.1
AptD		BGC0002512	QNL14925.1
AnaD		BGC0000302	ACZ55946.1
NpuD		BGC0001479	ACC81024.1
ApnD		BGC0000301	ABV79988.1
ApnC2		BGC0000301	ABV79987.1
AnaC2		BGC0000302	ACZ55944.1
AptC2		BGC0002512	QNL14923.1
NpuC2		BGC0001479	ACC81023.1
Tcp12	X	BGC0000440	CAE53353.1
CepC		BGC0000322	CAA11796.1
BpsC		BGC0000311	CAC48362.1

Table S4. Comparison of the active site motif of different types of C domains.

Type	Active site	Example
Amide formation ($^{L}C_{L}$, $^{D}C_{L}$)	HHXXXDG	SrfAC[43]
Starter	HHXXXDG	SrfAA[44]
C_{modAA} (dehydroamino acid synthesis)	HHXXXDG(WS)	AmbE[45]
E (epimerization)	HHXXXDXXSW	TycA[46], GrsA[47]
Dual (epimerization+ $^{D}C_{L}$)	HHXXXD and a second HHI/L(X)$_4$GD in the N-terminal region	ArfA[48]

β-lactam formation	HHHXXXDG	NocB-C_5[49]
Cyc (condensation+ hetero-cyclization)	DXXXXD	BmdB[50]
X (P_{450} recruitment)	HRXXXDD	Tcp12[51]
C_{urea} (ureido formation)	EHHXXHDG	SylC,[29] PacN[20]

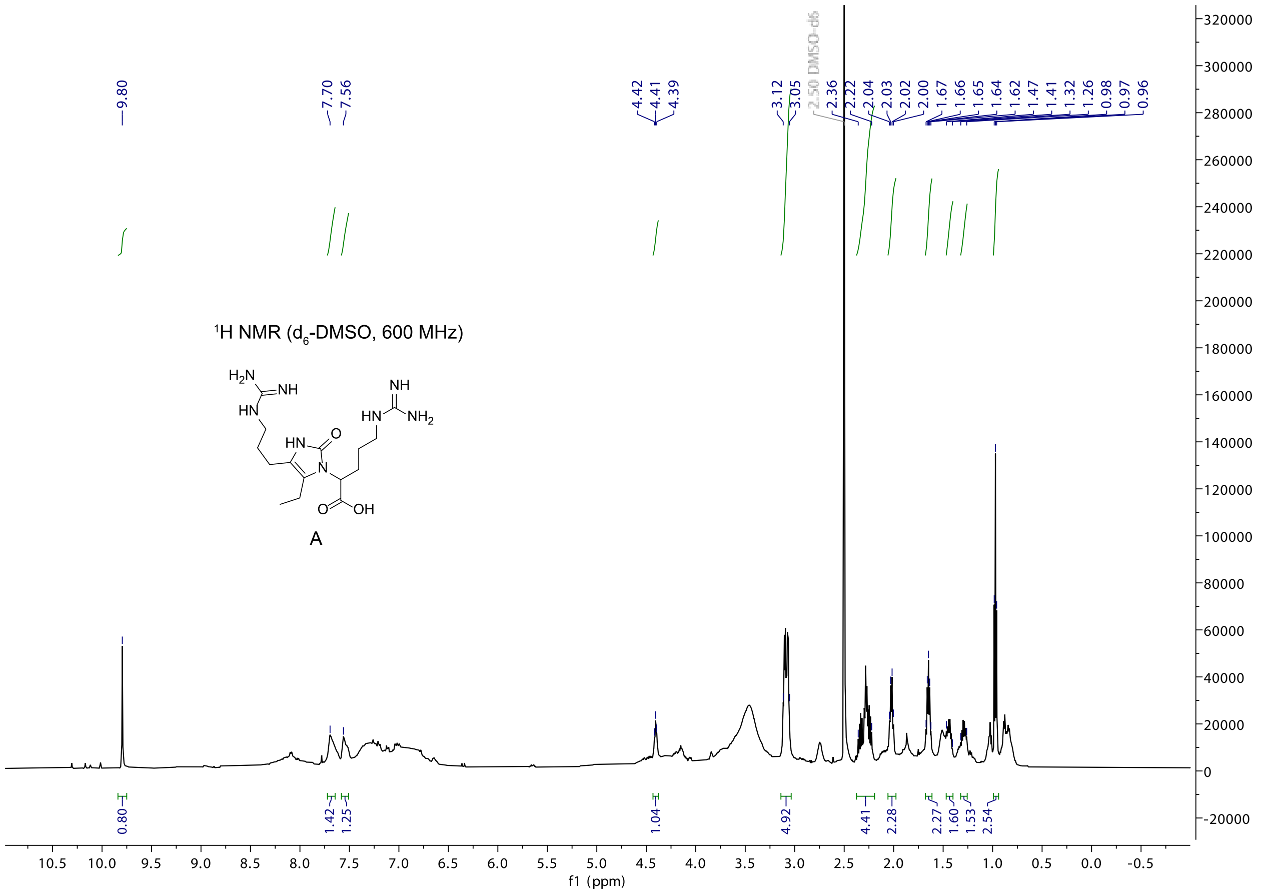

Figure S14. ^{1}H NMR spectrum of compound **1** collected in d_6-DMSO.

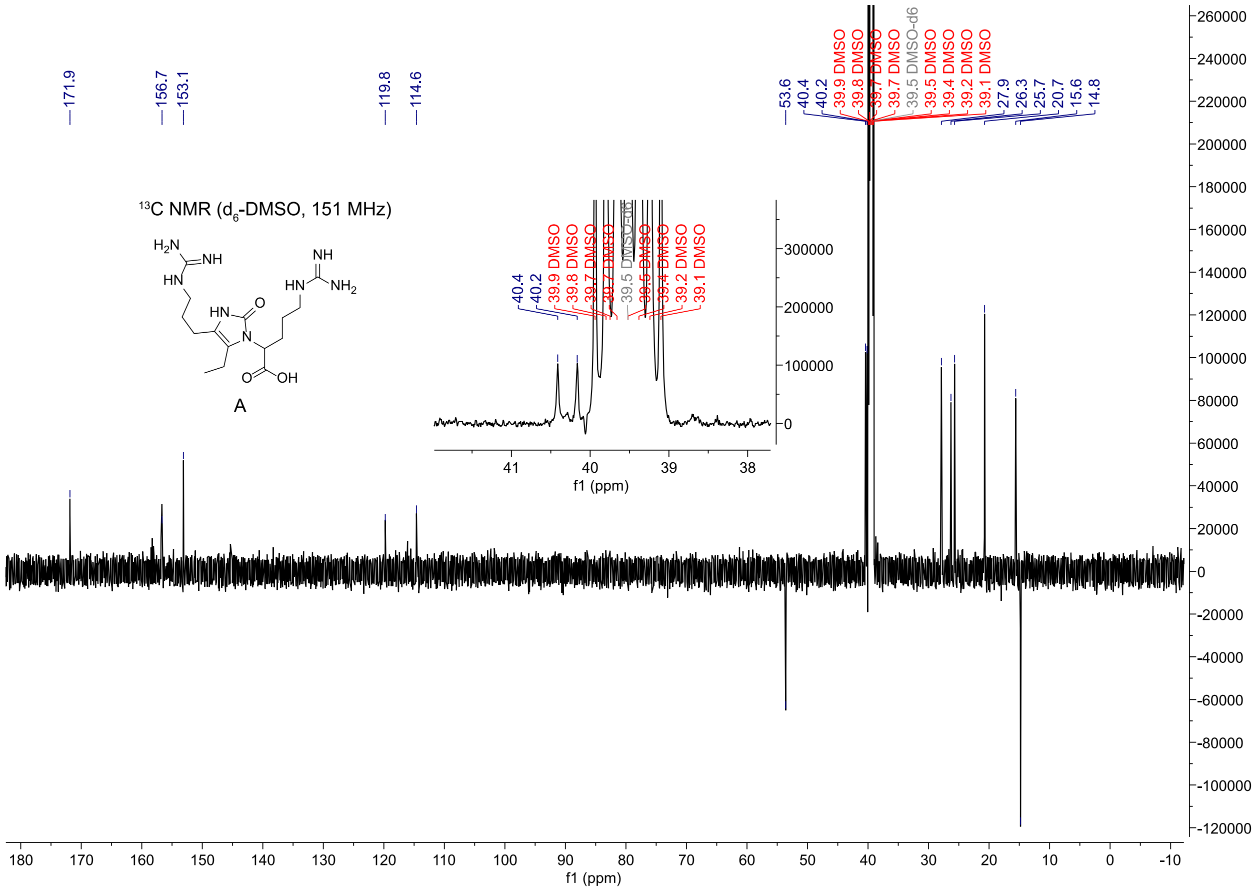

Figure S15. ^{13}C NMR spectrum of compound **1** collected in d_6-DMSO.

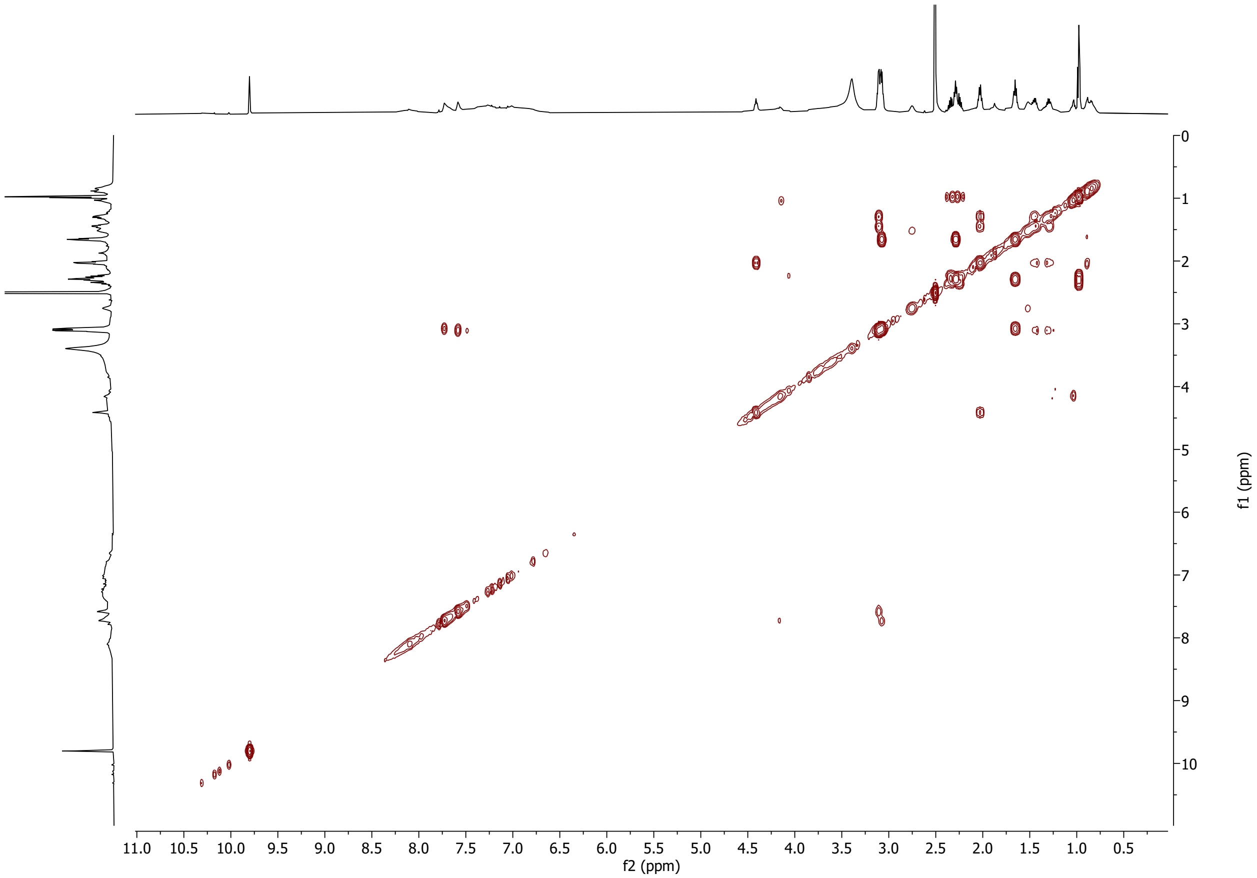

Figure S16. ^1H-^1H correlation spectroscopy (COSY) spectrum of compound **1** collected in d_6-DMSO.

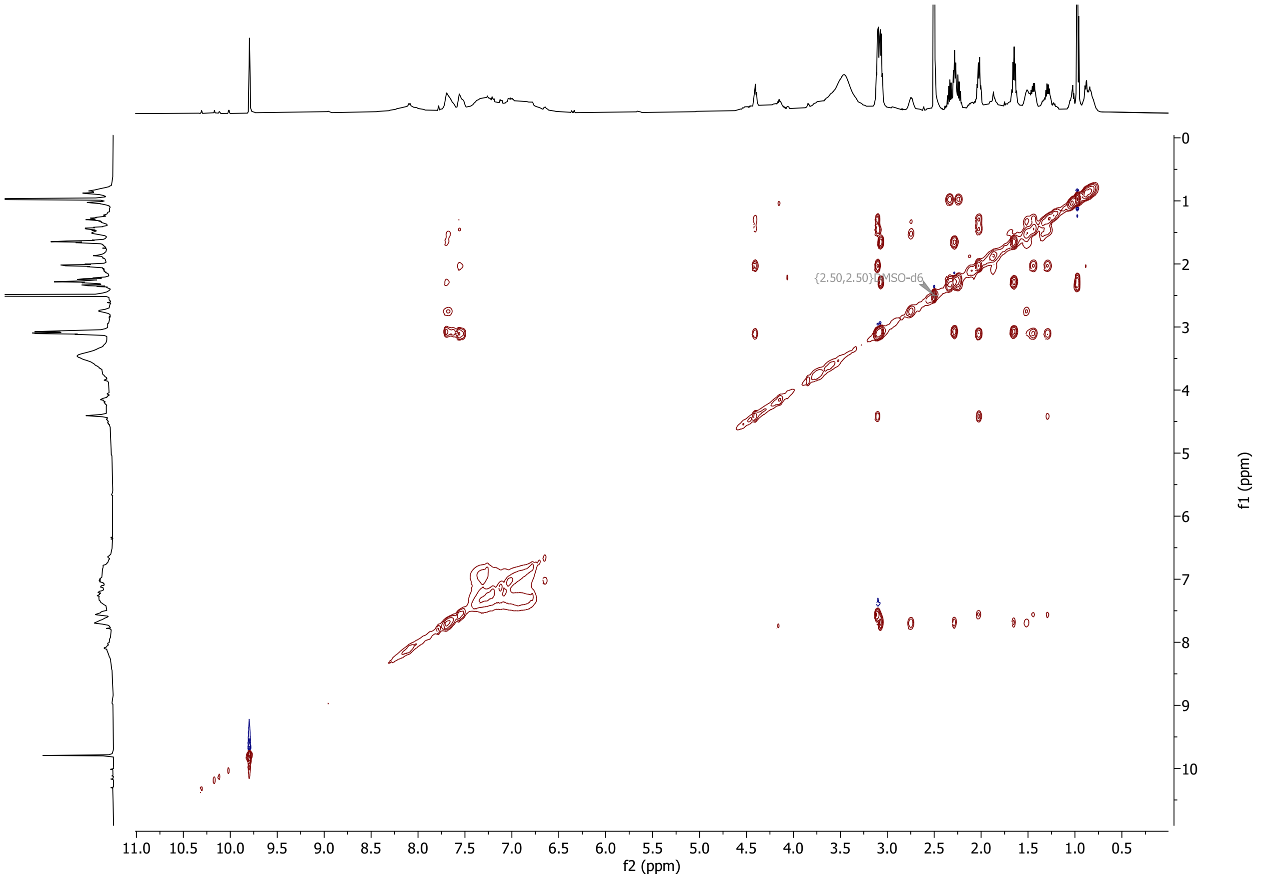

Figure S17. ^1H-^1H total correlation spectroscopy (TOCSY) spectrum of compound **1** collected in d_6-DMSO.

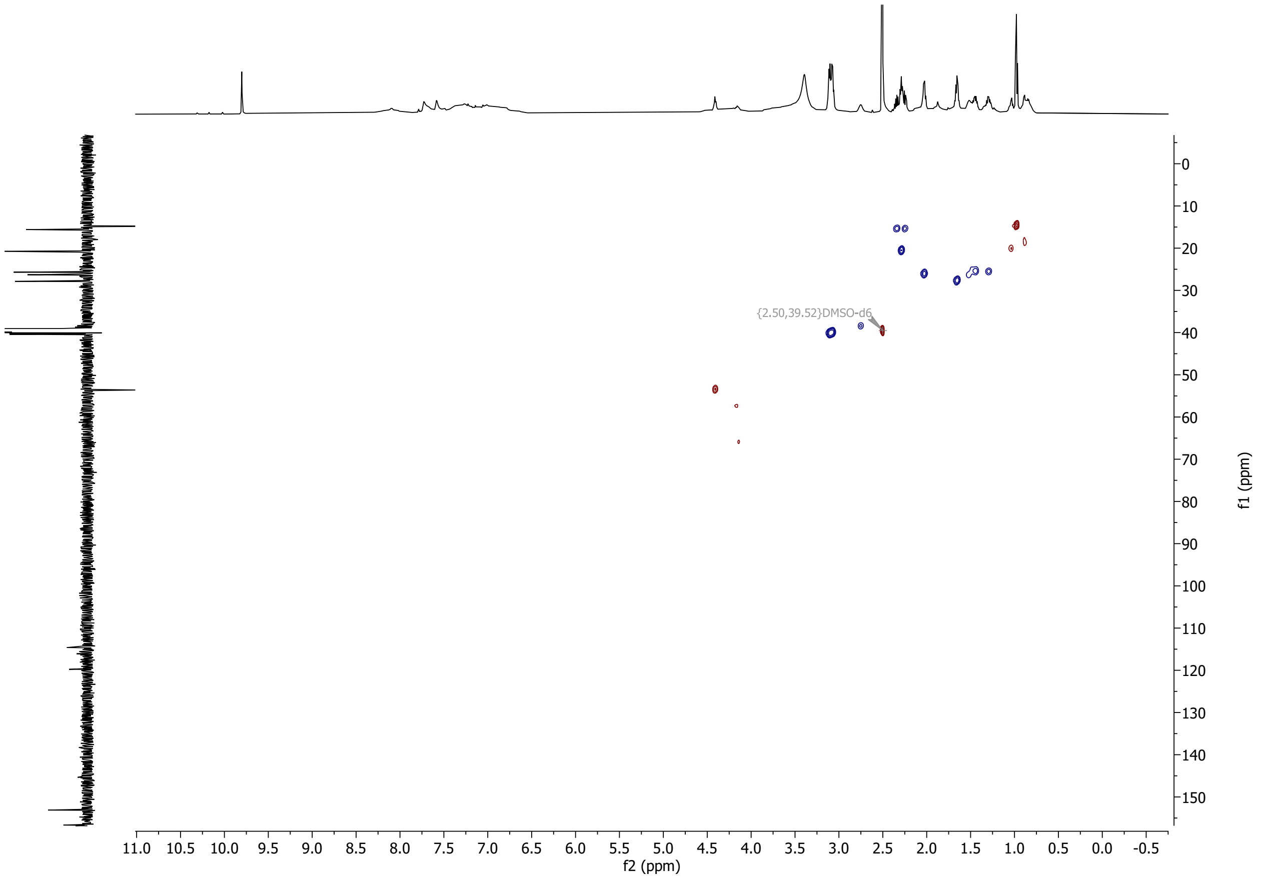

Figure S18. (^{1}H,^{13}C) heteronuclear single quantum coherence (HSQC) spectrum of compound **1** collected in d_6-DMSO.

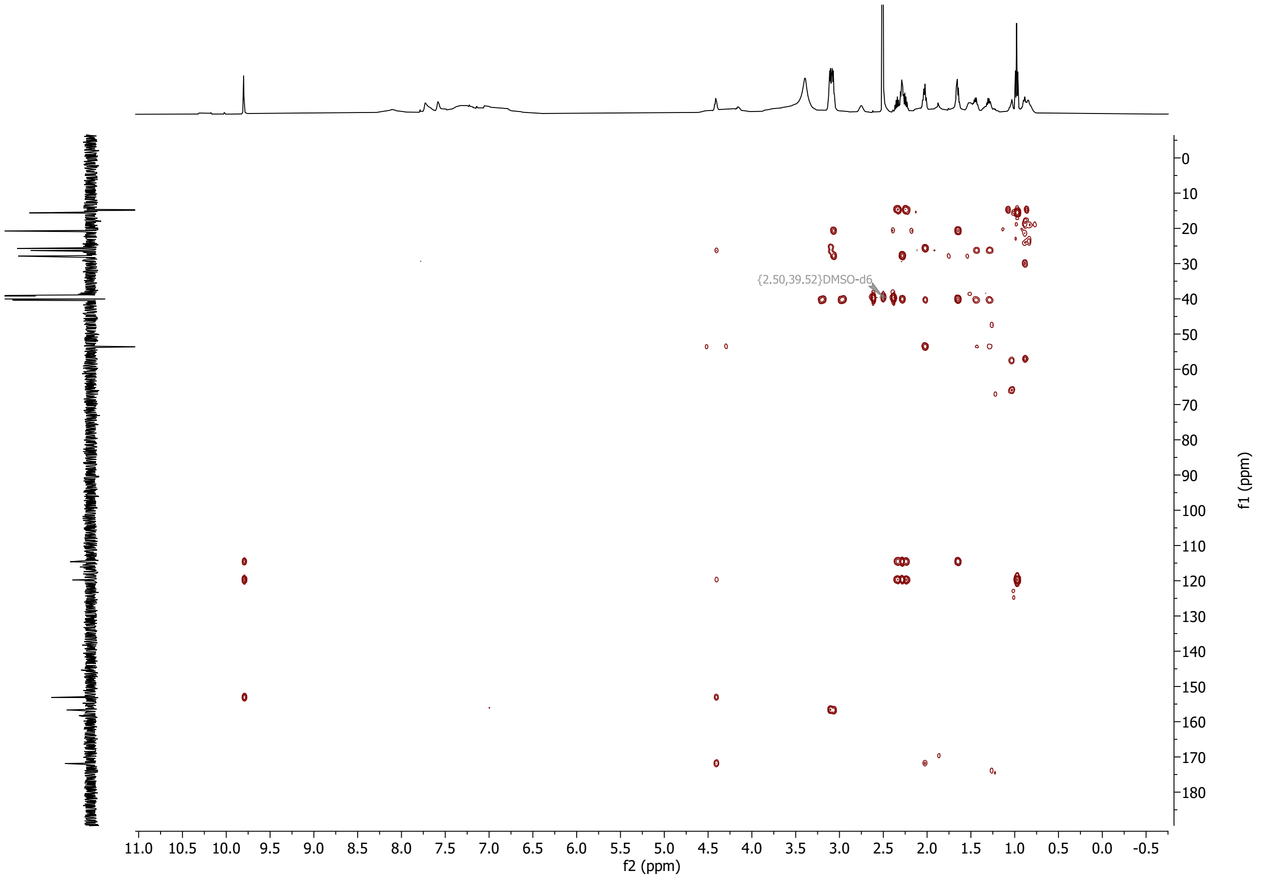

Figure S19. (^{1}H,^{13}C) heteronuclear multiple bond correlation (HMBC) spectrum of compound **1** collected in d_6-DMSO.

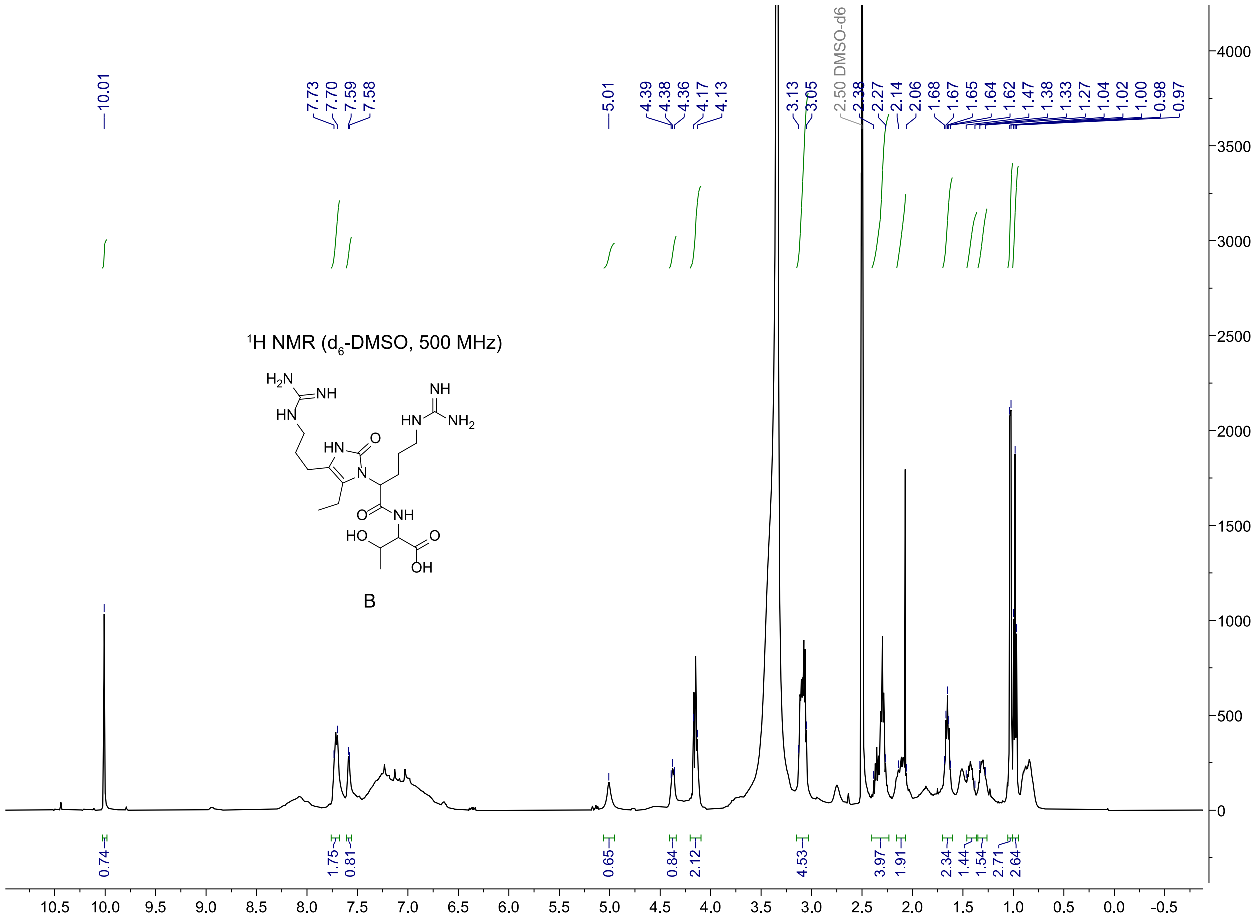

Figure S20. ^{1}H NMR spectrum of compound **2** collected in d_6-DMSO.

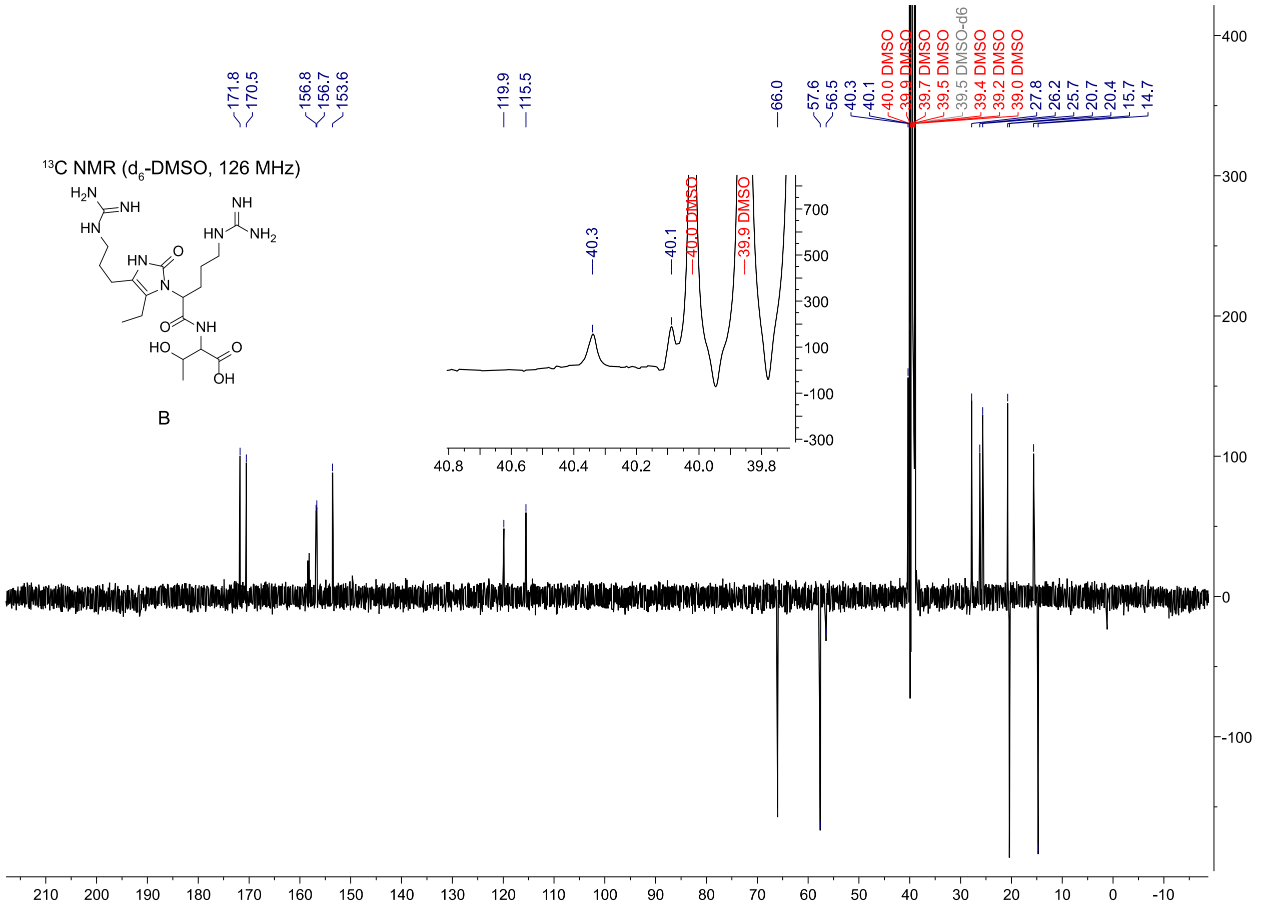

Figure S21. ^{13}C NMR spectrum of compound **2** collected in d_6-DMSO.

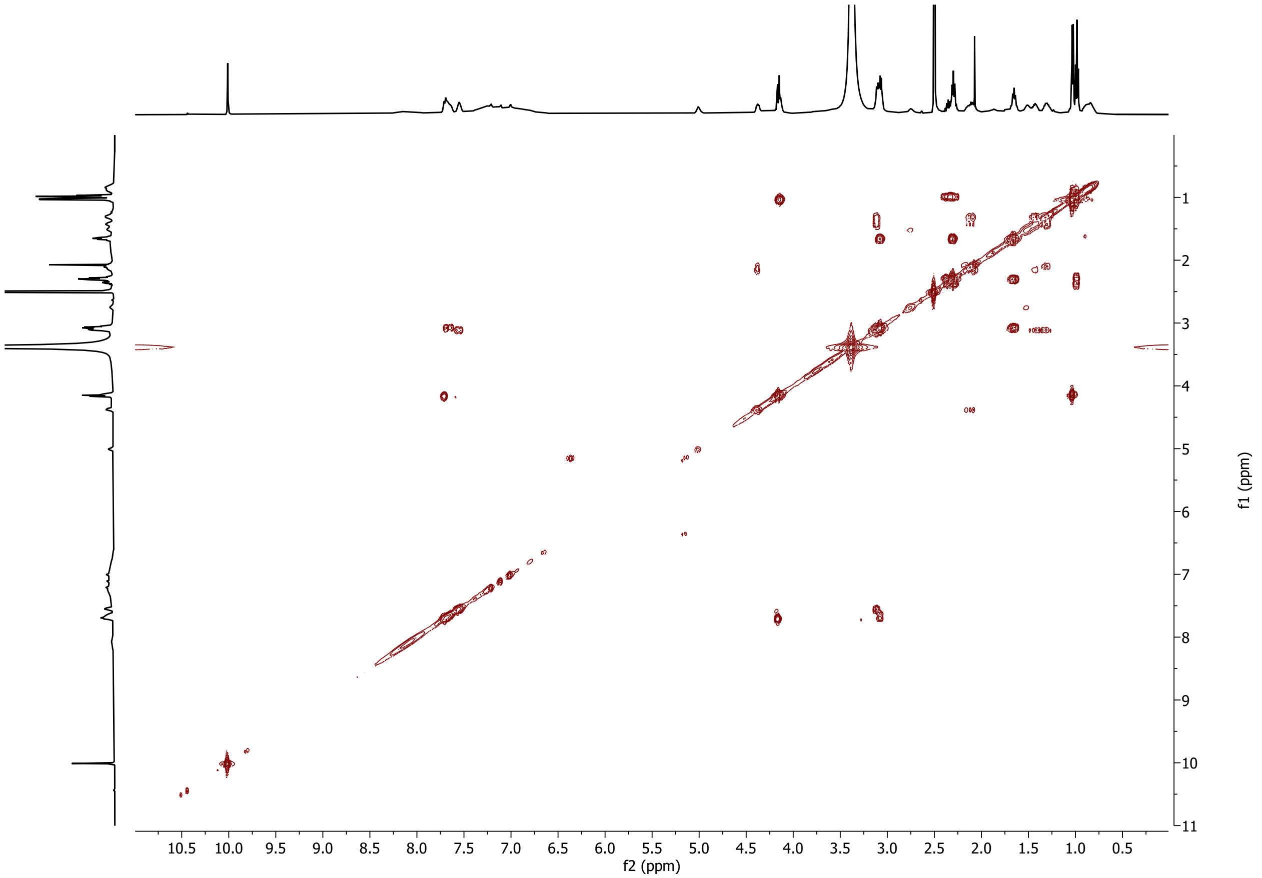

Figure S22. ^{1}H-^{1}H correlation spectroscopy (COSY) spectrum of compound **2** collected in d_6-DMSO.

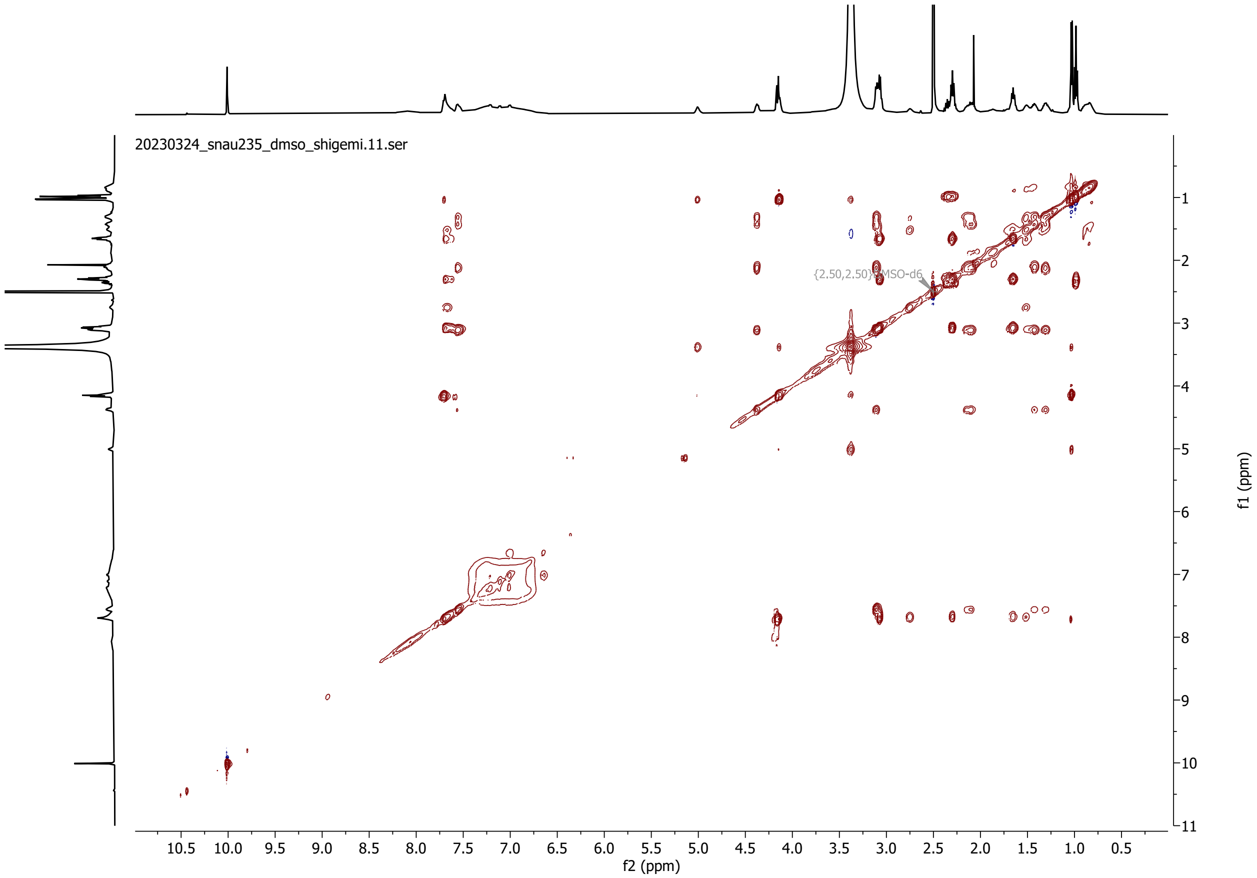

Figure S23. ^{1}H-^{1}H total correlation spectroscopy (TOCSY) spectrum of compound **2** collected in d_6-DMSO.

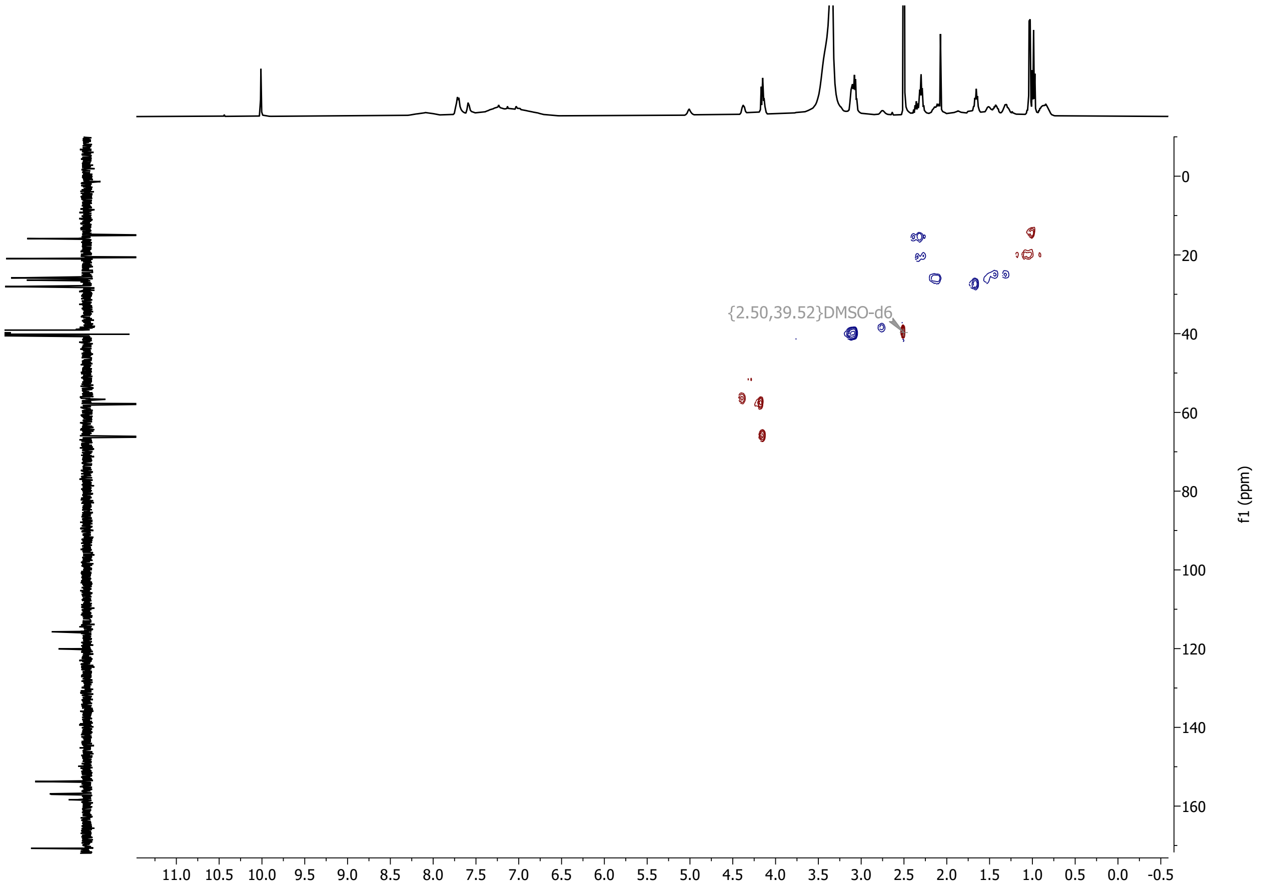

Figure S24. (^{1}H,^{13}C) heteronuclear single quantum coherence (HSQC) spectrum of compound **2** collected in d_6-DMSO.

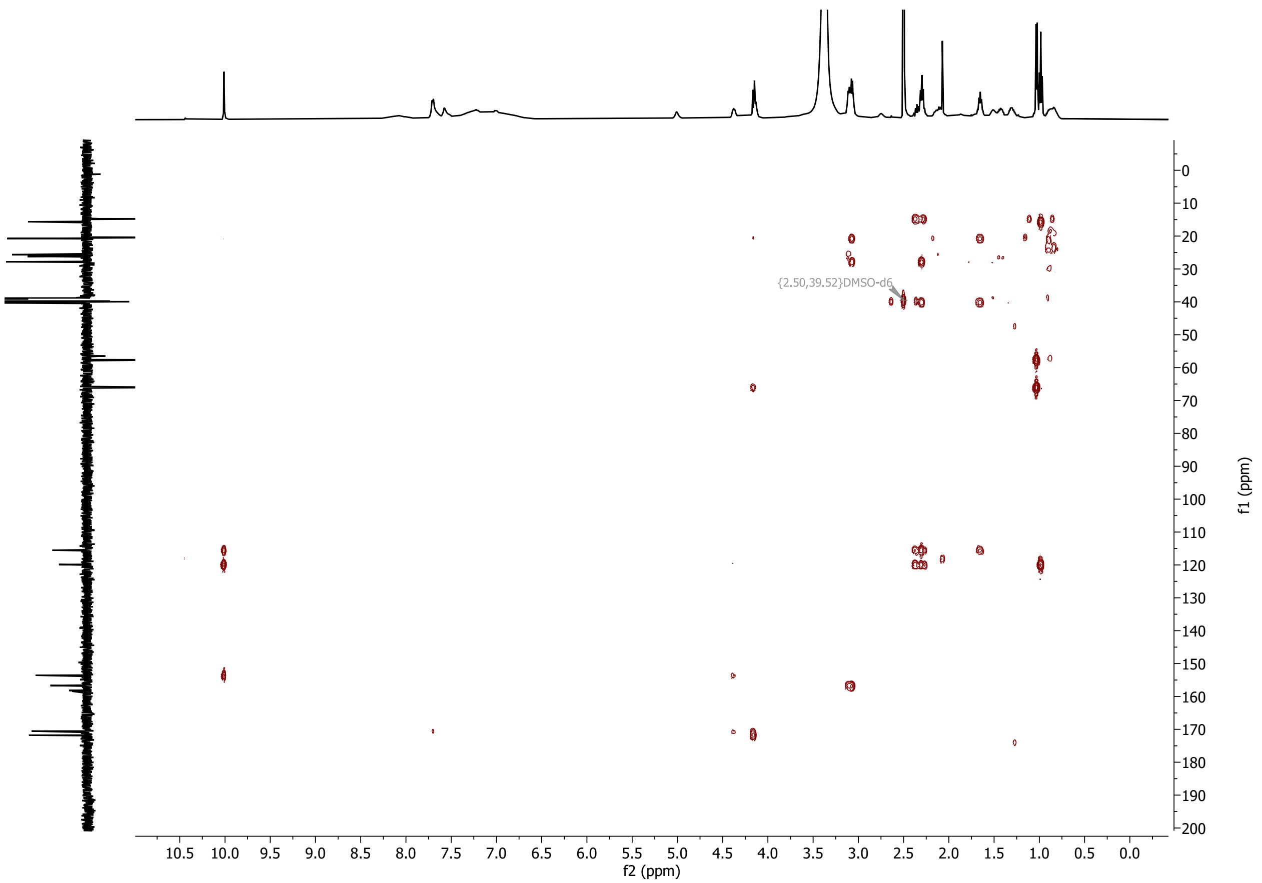

Figure S25. (^{1}H,^{13}C) heteronuclear multiple bond correlation (HMBC) spectrum of compound **2** collected in d_6-DMSO.

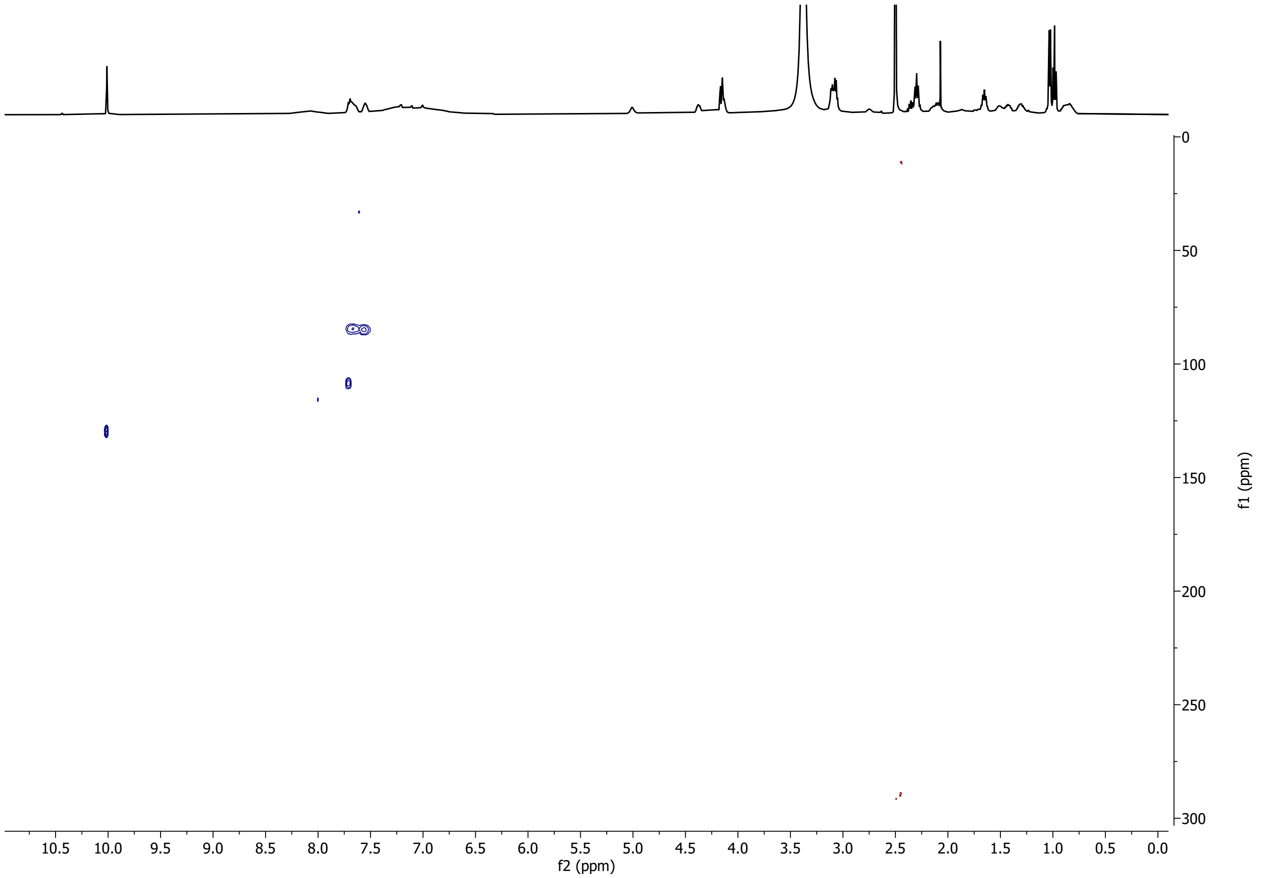

Figure S26. (^1H,^{15}N) heteronuclear single quantum coherence (HSQC) spectrum of compound **2** collected in d_6-DMSO.

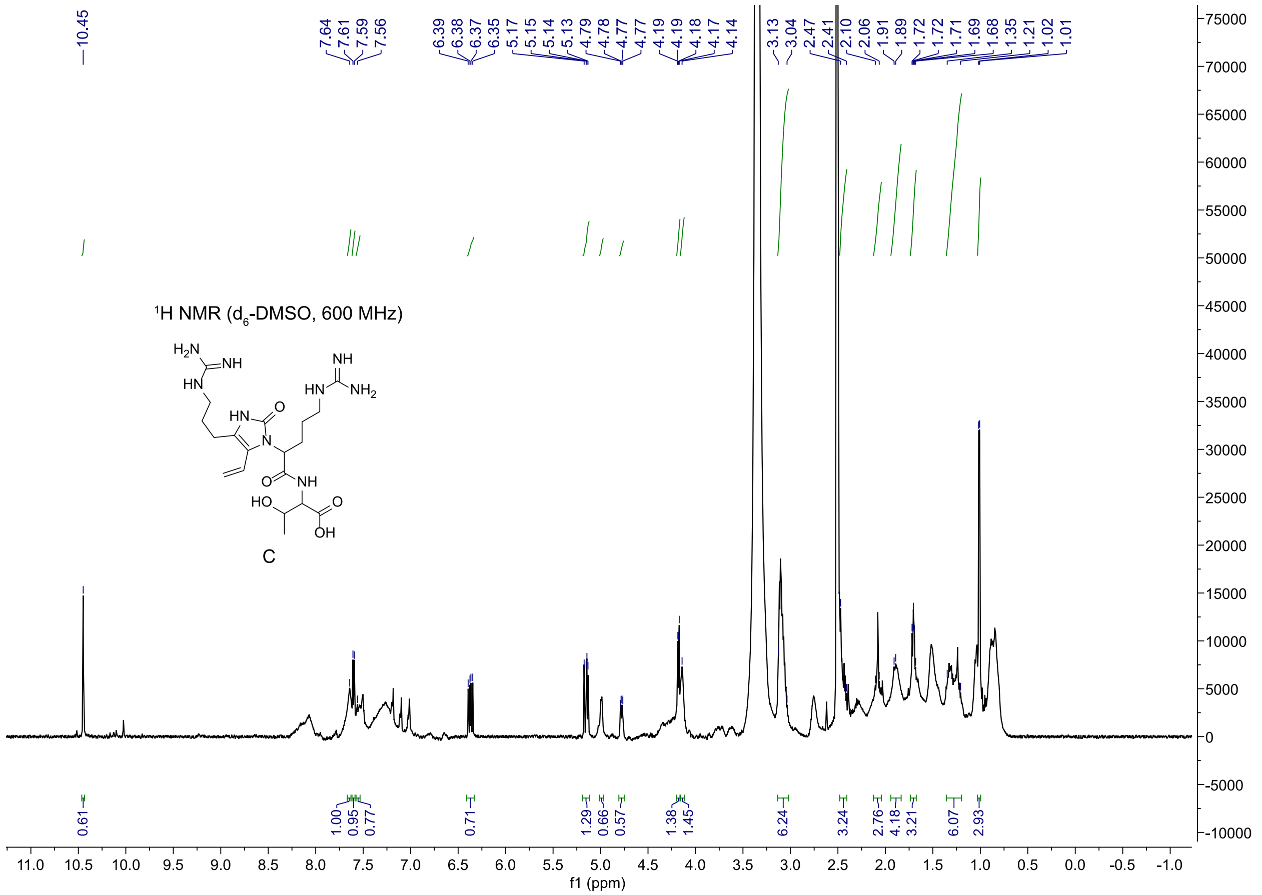

Figure S27. ^{1}H NMR spectrum of compound **3** collected in d$_6$-DMSO.

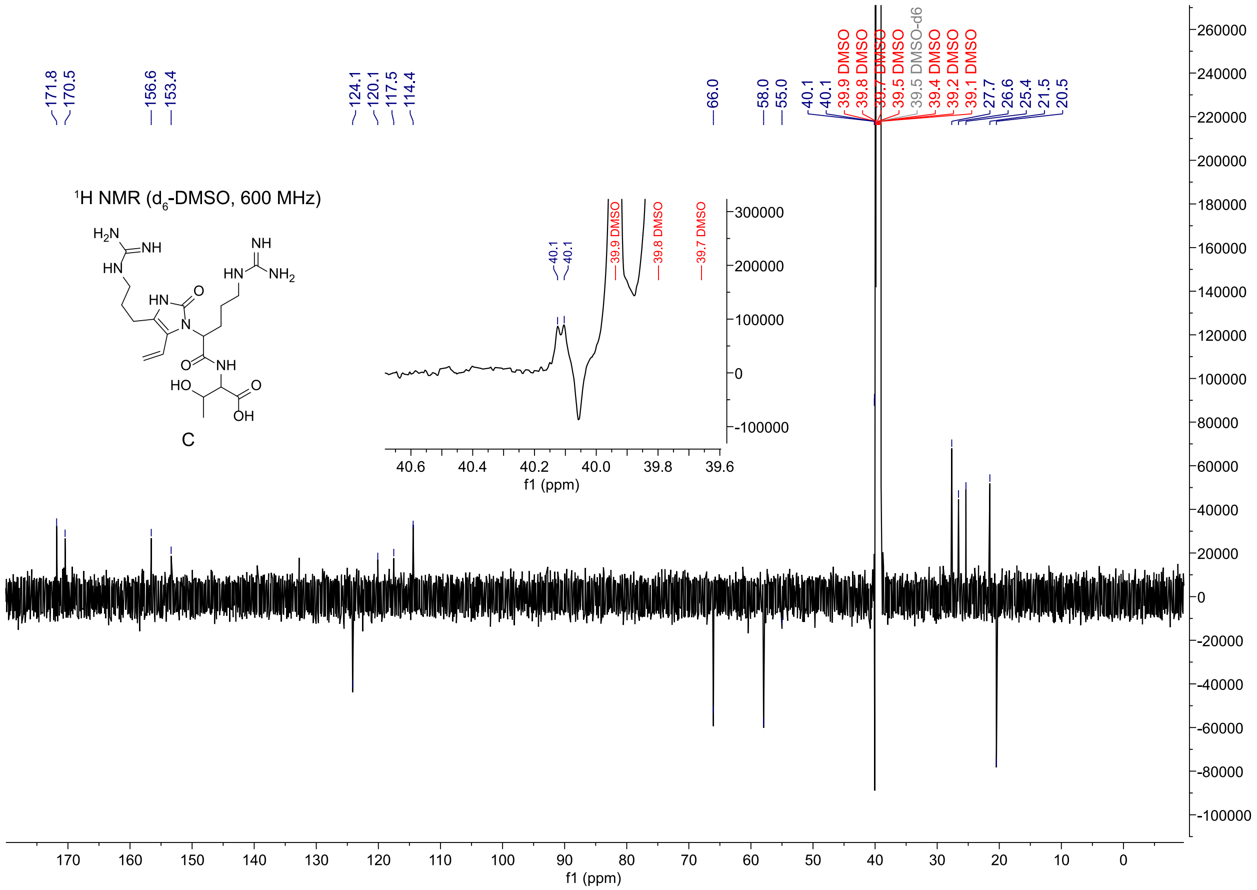

Figure S28. ^{13}C NMR spectrum of compound **3** collected in d_6-DMSO.

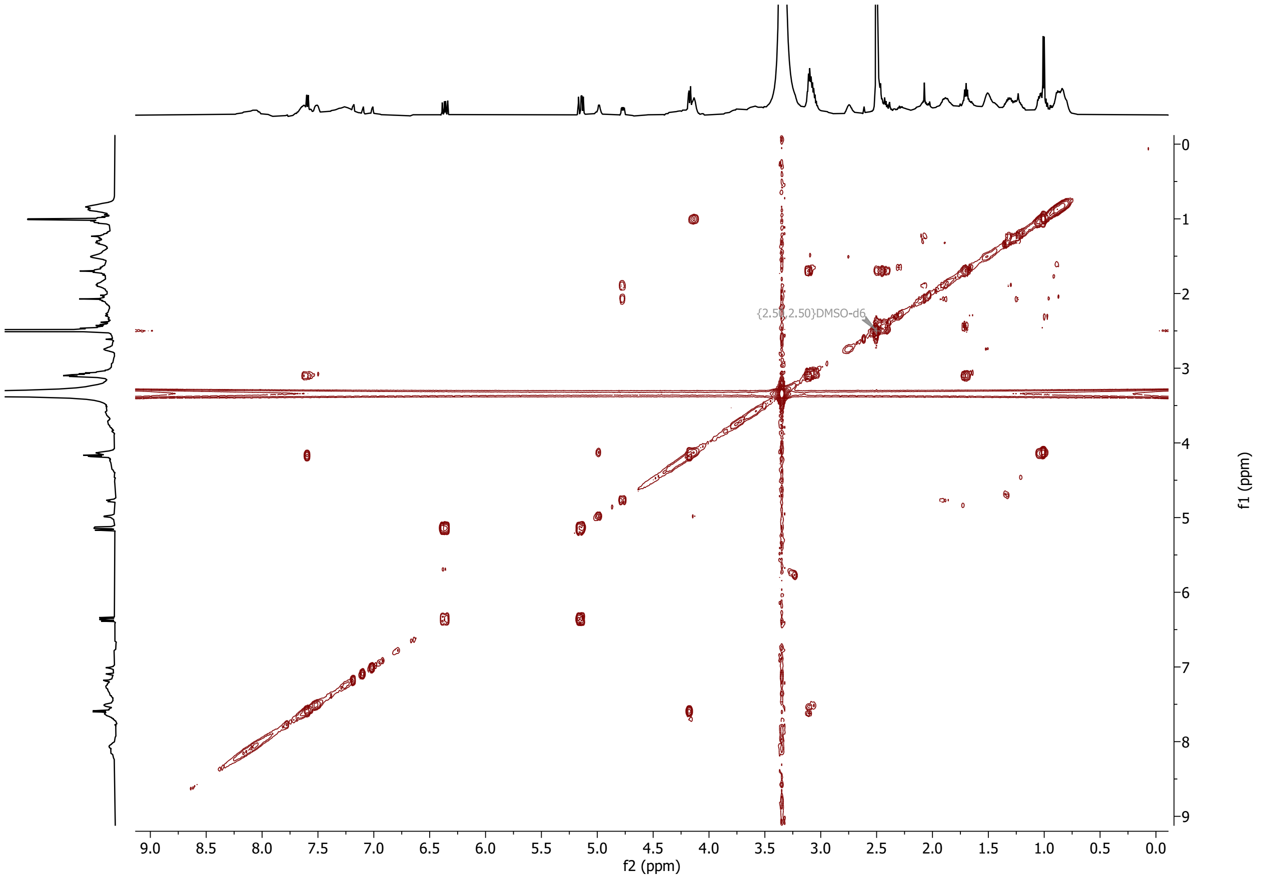

Figure S29. ^1H-^1H correlation spectroscopy (COSY) spectrum of compound **3** collected in d_6-DMSO.

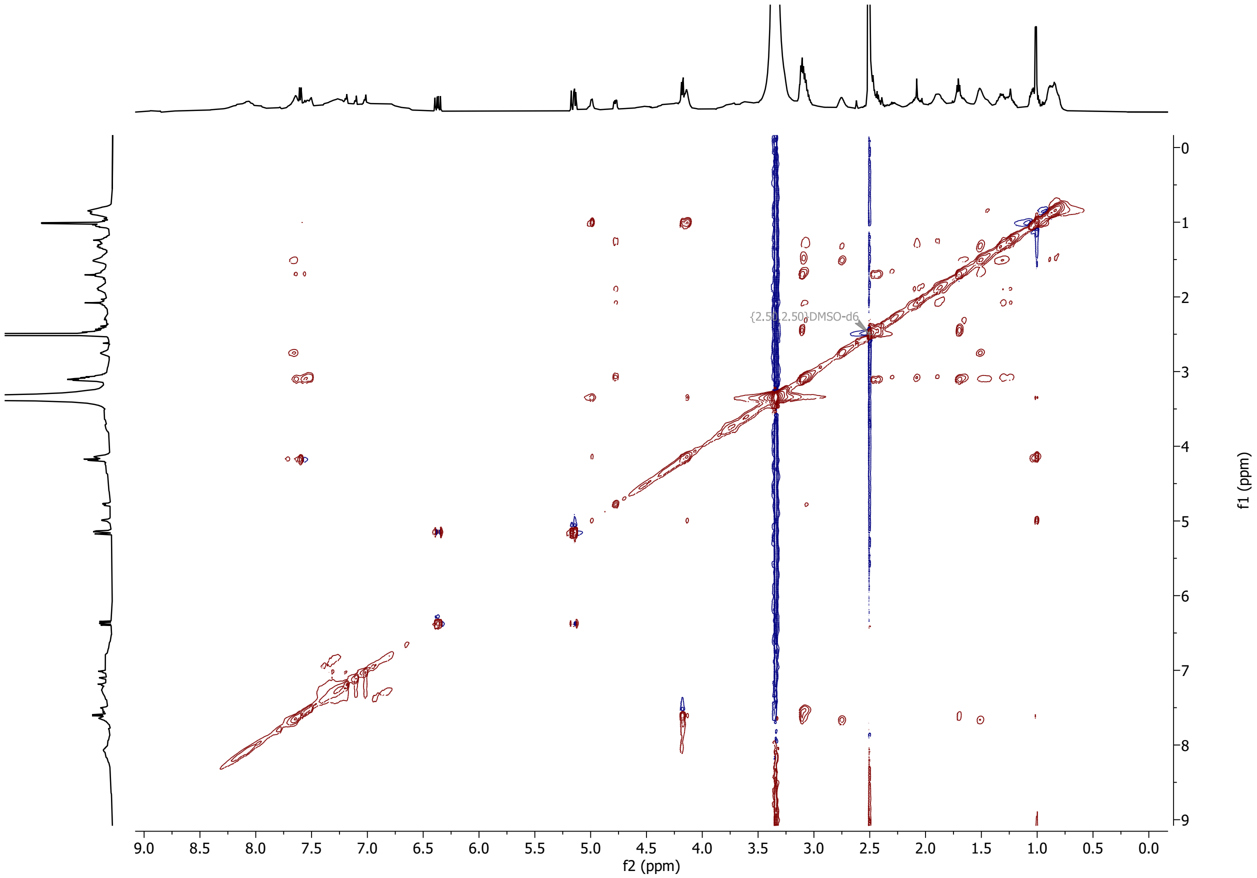

Figure S30. ^{1}H-^{1}H total correlation spectroscopy (TOCSY) spectrum of compound **3** collected in d_6-DMSO.

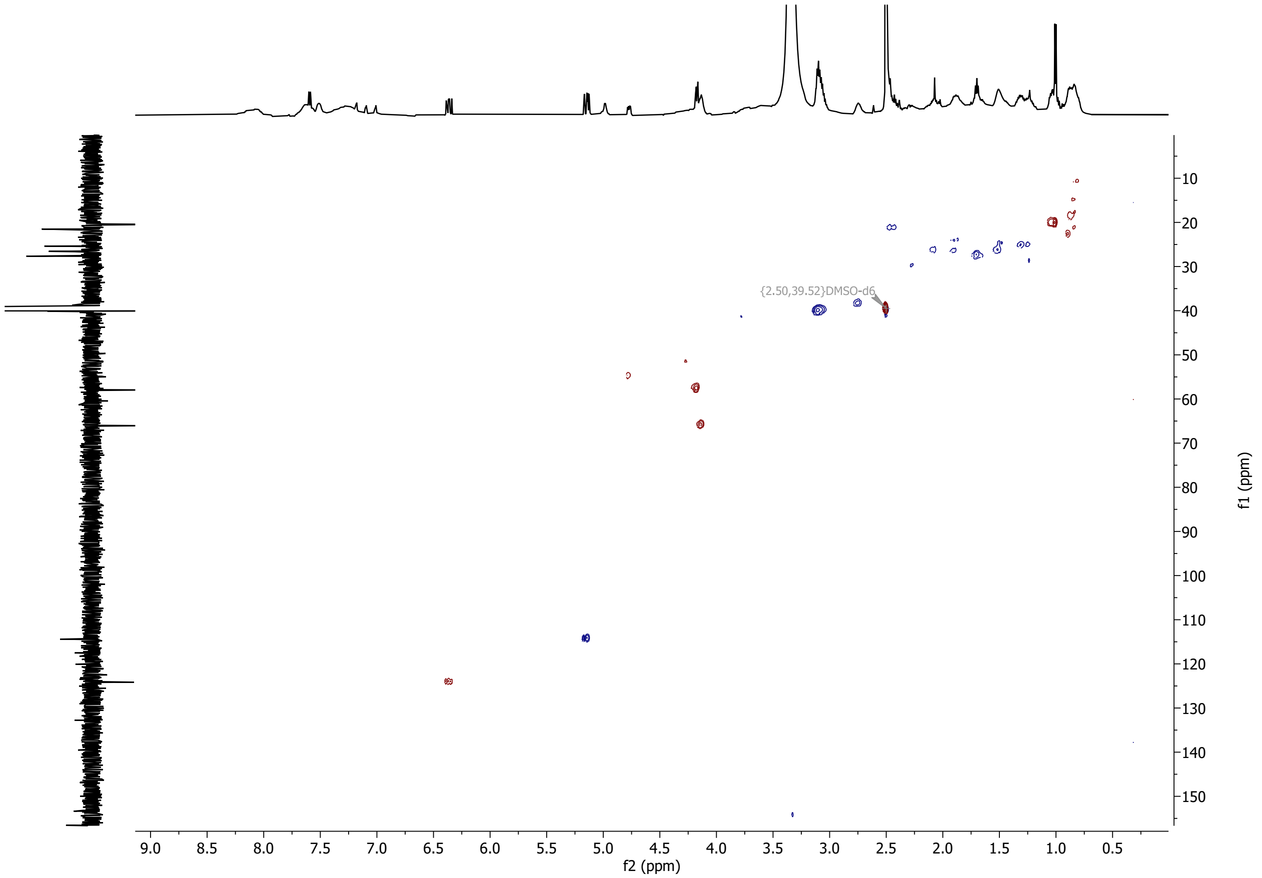

Figure S31. (^1H,^{13}C) heteronuclear single quantum coherence (HSQC) spectrum of compound **3** collected in d_6-DMSO.

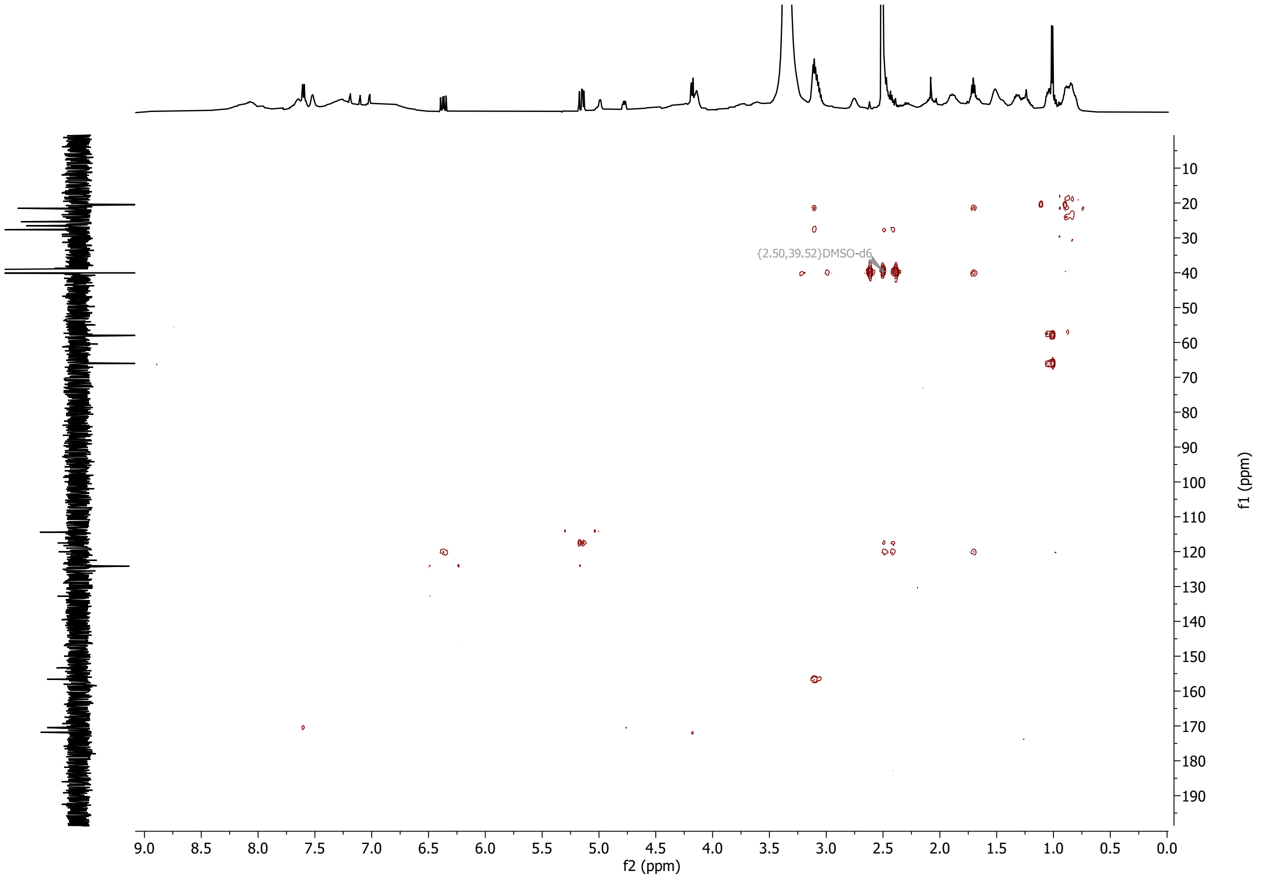

Figure S32. (^{1}H,^{13}C) heteronuclear multiple bond correlation (HMBC) spectrum of compound **3** collected in d$_6$-DMSO.

References

(1) Bai, C.; Zhang, Y.; Zhao, X.; Hu, Y.; Xiang, S.; Miao, J.; Lou, C.; Zhang, L. Exploiting a precise design of universal synthetic modular regulatory elements to unlock the microbial natural products in *Streptomyces*. *Proc. Natl. Acad. Sci. U. S. A.* **2015**, *112* (39), 12181-12186.

(2) Gibson, D. G.; Young, L.; Chuang, R. Y.; Venter, J. C.; Hutchison, C. A., 3rd; Smith, H. O. Enzymatic assembly of DNA molecules up to several hundred kilobases. *Nat. Methods* **2009**, *6* (5), 343-345.

(3) Kieser, T.; Bibb, M. J.; Buttner, M. J.; Chater, K. F.; Hopwood, D. A. Practical Streptomyces Genetics. **2000**.

(4) Kim, W.; Hwang, S.; Lee, N.; Lee, Y.; Cho, S.; Palsson, B.; Cho, B. K. Transcriptome and translatome profiles of Streptomyces species in different growth phases. *Sci. Data* **2020**, *7* (1), 138.

(5) Chambers, M. C.; Maclean, B.; Burke, R.; Amodei, D.; Ruderman, D. L.; Neumann, S.; Gatto, L.; Fischer, B.; Pratt, B.; Egertson, J.; et al. A cross-platform toolkit for mass spectrometry and proteomics. *Nat. Biotechnol.* **2012**, *30* (10), 918-920.

(6) Smith, C. A.; Want, E. J.; O'Maille, G.; Abagyan, R.; Siuzdak, G. XCMS: processing mass spectrometry data for metabolite profiling using nonlinear peak alignment, matching, and identification. *Anal. Chem.* **2006**, *78* (3), 779-787.

(7) Vijayasarathy, S.; Prasad, P.; Fremlin, L. J.; Ratnayake, R.; Salim, A. A.; Khalil, Z.; Capon, R. J. C3 and 2D C3 Marfey's methods for amino acid analysis in natural products. *J. Nat. Prod.* **2016**, *79* (2), 421-427.

(8) Bhushan, R.; Brückner, H. Marfey's reagent for chiral amino acid analysis: a review. *Amino Acids* **2004**, *27* (3-4), 231-247.

(9) Pérez-Victoria, I.; Crespo, G.; Reyes, F. Expanding the utility of Marfey's analysis by using HPLC-SPE-NMR to determine the C(β) configuration of threonine and isoleucine residues in natural peptides. *Analytical and bioanalytical chemistry* **2022**, *414* (28), 8063-8070.

(10) Maxson, T.; Tietz, J. I.; Hudson, G. A.; Guo, X. R.; Tai, H. C.; Mitchell, D. A. Targeting Reactive Carbonyls for Identifying Natural Products and Their Biosynthetic Origins. *J. Am. Chem. Soc.* **2016**, *138* (46), 15157-15166.

(11) Iorio, M.; Davatgarbenam, S.; Serina, S.; Criscenzo, P.; Zdouc, M. M.; Simone, M.; Maffioli, S. I.; Ebright, R. H.; Donadio, S.; Sosio, M. Blocks in the pseudouridimycin pathway unlock hidden metabolites in the *Streptomyces* producer strain. *Scientific reports* **2021**, *11* (1), 5827.

(12) Cui, Z.; Nguyen, H.; Bhardwaj, M.; Wang, X.; Büschleb, M.; Lemke, A.; Schütz, C.; Rohrbacher, C.; Junghanns, P.; Koppermann, S.; et al. Enzymatic C(β)-H functionalization of L-Arg and L-Leu in nonribosomally derived peptidyl natural products: a tale of two oxidoreductases. *J. Am. Chem. Soc.* **2021**, *143* (46), 19425-19437.

(13) Christiansen, G.; Philmus, B.; Hemscheidt, T.; Kurmayer, R. Genetic variation of adenylation domains of the anabaenopeptin synthesis operon and evolution of substrate promiscuity. *J. Bacteriol.* **2011**, *193* (15), 3822-3831.

(14) Rouhiainen, L.; Jokela, J.; Fewer, D. P.; Urmann, M.; Sivonen, K. Two alternative starter modules for the non-ribosomal biosynthesis of specific anabaenopeptin variants in *Anabaena* (Cyanobacteria). *Chem. Biol.* **2010**, *17* (3), 265-273.

(15) Greunke, C.; Duell, E. R.; D'Agostino, P. M.; Glöckle, A.; Lamm, K.; Gulder, T. A. M. Direct Pathway Cloning (DiPaC) to unlock natural product biosynthetic potential. *Metab. Eng.* **2018**, *47*, 334-345.

(16) Koketsu, K.; Mitsuhashi, S.; Tabata, K. Identification of homophenylalanine biosynthetic genes from the cyanobacterium *Nostoc punctiforme* PCC73102 and application to its microbial production by *Escherichia coli*. *Appl. Environ. Microbiol.* **2013**, *79* (7), 2201-2208.

(17) Saha, S.; Esposito, G.; Urajová, P.; Mareš, J.; Ewe, D.; Caso, A.; Macho, M.; Delawská, K.; Kust, A.; Hrouzek, P.; et al. Discovery of unusual cyanobacterial tryptophan-containing anabaenopeptins by MS/MS-based molecular networking. *Molecules* **2020**, *25* (17).

(18) Dudnik, A.; Bigler, L.; Dudler, R. Production of proteasome inhibitor syringolin A by the endophyte *Rhizobium* sp. strain AP16. *Appl. Environ. Microbiol.* **2014**, *80* (12), 3741-3748.
(19) Amrein, H.; Makart, S.; Granado, J.; Shakya, R.; Schneider-Pokorny, J.; Dudler, R. Functional analysis of genes involved in the synthesis of syringolin A by *Pseudomonas syringae* pv. *syringae* B301 D-R. *Mol. Plant. Microbe. Interact.* **2004**, *17* (1), 90-97.
(20) Zhang, W.; Ostash, B.; Walsh, C. T. Identification of the biosynthetic gene cluster for the pacidamycin group of peptidyl nucleoside antibiotics. *Proc. Natl. Acad. Sci. U. S. A.* **2010**, *107* (39), 16828-16833.
(21) Kaysser, L.; Tang, X.; Wemakor, E.; Sedding, K.; Hennig, S.; Siebenberg, S.; Gust, B. Identification of a napsamycin biosynthesis gene cluster by genome mining. *ChemBioChem* **2011**, *12* (3), 477-487.
(22) Tang, X.; Gross, M.; Xie, Y.; Kulik, A.; Gust, B. Identification of mureidomycin analogues and functional analysis of an N-acetyltransferase in napsamycin biosynthesis. *ChemBioChem* **2013**, *14* (17), 2248-2255.
(23) Cheng, L.; Chen, W.; Zhai, L.; Xu, D.; Huang, T.; Lin, S.; Zhou, X.; Deng, Z. Identification of the gene cluster involved in muraymycin biosynthesis from *Streptomyces* sp. NRRL 30471. *Mol. Biosyst.* **2011**, *7* (3), 920-927.
(24) Liu, J.; Zhou, H.; Yang, Z.; Wang, X.; Chen, H.; Zhong, L.; Zheng, W.; Niu, W.; Wang, S.; Ren, X.; et al. Rational construction of genome-reduced Burkholderiales chassis facilitates efficient heterologous production of natural products from proteobacteria. *Nat. Commun.* **2021**, *12* (1), 4347.
(25) Ióca, L. P.; Dai, Y.; Kunakom, S.; Diaz-Espinosa, J.; Krunic, A.; Crnkovic, C. M.; Orjala, J.; Sanchez, L. M.; Ferreira, A. G.; Berlinck, R. G. S.; Eustáquio, A. S. A family of nonribosomal peptides modulate collective behavior in *Pseudovibrio* bacteria isolated from marine sponges. *Angew. Chem. Int. Ed. Engl.* **2021**, *60* (29), 15891-15898.
(26) Zhong, W.; Deutsch, J. M.; Yi, D.; Abrahamse, N. H.; Mohanty, I.; Moore, S. G.; McShan, A. C.; Garg, N.; Agarwal, V. Discovery and biosynthesis of ureidopeptide natural products macrocyclized via indole N-acylation in marine *Microbulbifer spp.* Bacteria. *ChemBioChem* **2023**, *24* (12), e202300190.
(27) Zhong, W.; Aiosa, N.; Deutsch, J. M.; Garg, N.; Agarwal, V. Pseudobulbiferamides: plasmid-encoded ureidopeptide natural products with biosynthetic gene clusters shared among marine bacteria of different genera. *J. Nat. Prod.* **2023**, *86* (10), 2414-2420.
(28) Kautsar, S. A.; Blin, K.; Shaw, S.; Navarro-Muñoz, J. C.; Terlouw, B. R.; van der Hooft, J. J. J.; van Santen, J. A.; Tracanna, V.; Suarez Duran, H. G.; Pascal Andreu, V.; et al. MIBiG 2.0: a repository for biosynthetic gene clusters of known function. *Nucleic Acids Research* **2020**, *48* (D1), D454-D458.
(29) Imker, H. J.; Walsh, C. T.; Wuest, W. M. SylC catalyzes ureido-bond formation during biosynthesis of the proteasome inhibitor syringolin A. *J. Am. Chem. Soc.* **2009**, *131* (51), 18263-18265.
(30) Guindon, S.; Dufayard, J. F.; Lefort, V.; Anisimova, M.; Hordijk, W.; Gascuel, O. New algorithms and methods to estimate maximum-likelihood phylogenies: assessing the performance of PhyML 3.0. *Systematic biology* **2010**, *59* (3), 307-321.
(31) Guindon, S.; Delsuc, F.; Dufayard, J. F.; Gascuel, O. Estimating maximum likelihood phylogenies with PhyML. *Methods in molecular biology (Clifton, N.J.)* **2009**, *537*, 113-137.
(32) Lefort, V.; Longueville, J. E.; Gascuel, O. SMS: smart model selection in PhyML. *Molecular biology and evolution* **2017**, *34* (9), 2422-2424.
(33) Letunic, I.; Bork, P. Interactive Tree of Life (iTOL) v6: recent updates to the phylogenetic tree display and annotation tool. *Nucleic Acids Res.* **2024**.
(34) Garg, N.; Salazar-Ocampo, L. M.; van der Donk, W. A. In vitro activity of the nisin dehydratase NisB. *Proc. Natl. Acad. Sci. U. S. A.* **2013**, *110* (18), 7258-7263.
(35) Ortega, M. A.; Hao, Y.; Walker, M. C.; Donadio, S.; Sosio, M.; Nair, S. K.; van der Donk, W. A. Structure and tRNA specificity of MibB, a lantibiotic dehydratase from Actinobacteria involved in NAI-107 biosynthesis. *Cell chemical biology* **2016**, *23* (3), 370-380.

(36) Xie, L.; Chatterjee, C.; Balsara, R.; Okeley, N. M.; van der Donk, W. A. Heterologous expression and purification of SpaB involved in subtilin biosynthesis. *Biochem. Biophys. Res. Commun.* **2002**, *295* (4), 952-957.
(37) Peschel, A.; Ottenwälder, B.; Götz, F. Inducible production and cellular location of the epidermin biosynthetic enzyme EpiB using an improved staphylococcal expression system. *FEMS Microbiol. Lett.* **1996**, *137* (2-3), 279-284.
(38) Bothwell, I. R.; Cogan, D. P.; Kim, T.; Reinhardt, C. J.; van der Donk, W. A.; Nair, S. K. Characterization of glutamyl-tRNA-dependent dehydratases using nonreactive substrate mimics. *Proc. Natl. Acad. Sci. U. S. A.* **2019**, *116* (35), 17245-17250.
(39) Zhang, Z.; van der Donk, W. A. Nonribosomal peptide extension by a peptide amino-acyl tRNA ligase. *J. Am. Chem. Soc.* **2019**, *141* (50), 19625-19633.
(40) Daniels, P. N.; Lee, H.; Splain, R. A.; Ting, C. P.; Zhu, L.; Zhao, X.; Moore, B. S.; van der Donk, W. A. A biosynthetic pathway to aromatic amines that uses glycyl-tRNA as nitrogen donor. *Nat. Chem.* **2022**, *14* (1), 71-77.
(41) Ting, C. P.; Funk, M. A.; Halaby, S. L.; Zhang, Z.; Gonen, T.; van der Donk, W. A. Use of a scaffold peptide in the biosynthesis of amino acid-derived natural products. *Science* **2019**, *365* (6450), 280-284.
(42) Ramos-Figueroa, J.; Zhu, L.; van der Donk, W. Unexpected transformations during pyrroloiminoquinone biosynthesis. *bioRxiv* **2024**, doi 10.1101/2024.1103.1112.584671. Posted March 584614, 582024 (accessed April 584623, 582024).
(43) Tanovic, A.; Samel, S. A.; Essen, L. O.; Marahiel, M. A. Crystal structure of the termination module of a nonribosomal peptide synthetase. *Science* **2008**, *321* (5889), 659-663.
(44) Koumoutsi, A.; Chen, X. H.; Henne, A.; Liesegang, H.; Hitzeroth, G.; Franke, P.; Vater, J.; Borriss, R. Structural and functional characterization of gene clusters directing nonribosomal synthesis of bioactive cyclic lipopeptides in *Bacillus amyloliquefaciens* strain FZB42. *J. Bacteriol.* **2004**, *186* (4), 1084-1096.
(45) Patteson, J. B.; Fortinez, C. M.; Putz, A. T.; Rodriguez-Rivas, J.; Bryant, L. H., 3rd; Adhikari, K.; Weigt, M.; Schmeing, T. M.; Li, B. Structure and function of a dehydrating condensation domain in nonribosomal peptide biosynthesis. *J. Am. Chem. Soc.* **2022**, *144* (31), 14057-14070.
(46) Samel, S. A.; Czodrowski, P.; Essen, L. O. Structure of the epimerization domain of tyrocidine synthetase A. *Acta. Crystallogr. D Biol. Crystallogr.* **2014**, *70* (Pt 5), 1442-1452.
(47) Chen, W. H.; Li, K.; Guntaka, N. S.; Bruner, S. D. Interdomain and Intermodule Organization in Epimerization Domain Containing Nonribosomal Peptide Synthetases. *ACS. Chem. Biol.* **2016**, *11* (8), 2293-2303.
(48) Balibar, C. J.; Vaillancourt, F. H.; Walsh, C. T. Generation of D amino acid residues in assembly of arthrofactin by dual condensation/epimerization domains. *Chem. Biol.* **2005**, *12* (11), 1189-1200.
(49) Gaudelli, N. M.; Long, D. H.; Townsend, C. A. β-Lactam formation by a non-ribosomal peptide synthetase during antibiotic biosynthesis. *Nature* **2015**, *520* (7547), 383-387.
(50) Bloudoff, K.; Fage, C. D.; Marahiel, M. A.; Schmeing, T. M. Structural and mutational analysis of the nonribosomal peptide synthetase heterocyclization domain provides insight into catalysis. *Proc. Natl. Acad. Sci. U. S. A.* **2017**, *114* (1), 95-100.
(51) Haslinger, K.; Peschke, M.; Brieke, C.; Maximowitsch, E.; Cryle, M. J. X-domain of peptide synthetases recruits oxygenases crucial for glycopeptide biosynthesis. *Nature* **2015**, *521* (7550), 105-109.
