# Supplementary material for: PEARL-Catalyzed Peptide Bond Formation after Chain Reversal by Ureido-Forming Condensation Domains
Source: ACS Cent Sci. 2024 Jun 3;10(6):1242–50. doi: 10.1021/acscentsci.4c00044 (PMC11212132; doi:10.1021/acscentsci.4c00044)
Supplement: Supplementary file 4 — oc4c00044_si_004.pdf [file oc4c00044_si_004.pdf]

oc-2024-000445.R1

Name: Peer Review Information for "PEARL-catalyzed peptide bond formation after chain reversal by ureido-forming condensation domains"

First Round of Reviewer Comments

Reviewer: 1

Comments to the Author

This manuscript describes a fascinating example of biosynthetic ingenuity and reveals interesting enzyme function both in stand alone enzymes and NRPS domains. The paper is very succinctly written and the figure composition is such that I did find it hard to follow at times (in comparison the discussion is much longer and detailed). I have little complaint with the work as it is performed and it is well documented in the SI - I was somewhat disappointed that no further effort was made in investigating the mechanism of ureido formation as all the pieces are available to help distinguish this fascinating process. Given the ability to identify biosynthesis pathways to find examples of such modified C-domains I think this is an important point to address in more detail. I do not anticipate that this would be overly onerous, however given that this would require new mutants to be generated and tested I therefore chose the major revision option - however with this data included I would be very supportive of the publication of this work in ACS Central Science.

Specific further points:

There is no mention of C_domains that also for dehydro-residues, would seem something worth adding. The NRPS introduction in general is very brief for a broad audience.

Figure 1 is very light touch - the R groups are not differentiated for example, and this only really captures the C-domain mediated step (no mention of A/TE (or E) domains). As the discussion of ureido-forming C-domains is discussed below, this would also seem worthy of inclusion here for comparison. I don't see how panel C fits with this general figure unless there is a lot more information about the pathway (structures etc), as it is otherwise floating somewhat.

Figure 2 is again cut off from the BGC - plus the traces are tiny and colors very hard to differentiate. Also, I thought the mass of C should be 2Da less than B, rather than C (unless there is another

difference in the structures that I could not distinguish?) I think this should be integrated with figure 3 as this helps to also explain the predicted role of the gene products and clarifies the expectations of the deletion experiments.

The lack of sterochemistry is problematic. I appreciate that some intermediates are highly prone to cyclization, but why can't the remaining stereochemical centers be distinguished (e.g. by Marfey's analysis)? Given that the manuscript deals with atypical C-domain chemistry I would argue that the existence of dual functional condensation/ epimerase domains makes this a valid question to address.

Figure 4 panel A should be condensed/ show as a weblogo type image for clarity. How do such motifs compare to other examples of C-domain motifs (b-lactam formation etc)? What other intermediates are offloaded in these experiments (especially for the mutants) - this would help to clarify activation of substrates.

The discussion of the mechanism I found somewhat confusing - as there is access to the protein encoding SnaA it would be possible to distinguish the mechanisms in figure S6. The loading of multiple PCP domains by one A-domain has been reported, and thus I would think that the specificity of these two A-domains could be distinguished (ie is one activating bicarbonate and one loading both Arg residues?) Inspection of the substrate selection pockets of these A-domains would also yield valuable clues as to the likely substrates of these domains. Inactivation of PCP domains by Ser to Ala mutation would also allow intermediates to be isolated that would help to then show the timing of bicarbonate addition and to which Arg residue. I found it a little odd to see the discussion engaging with A-domain specificity from other pathways without explicitly addressing this for the system under investigation. Given the ability to assay C-domain mutants I would expect that these experiments should also prove feasible and relatively straightforward to accomplish and this would be a major contribution to this field.

Is there any MbtH protein in the producer? Are these A-domains happy without one for in vitro experiments?

My experience with chemistry journals leads me to have a preference for numbers for compounds rather than letters - especially when using many gene/protein names and many motif signatures that are all letters.

There are some legibility issues with figure S4.

Data in Figure S1 would be much easier to read when tabulated (as with S2/S3).

Reviewer: 2

Comments to the Author

The manuscript by Yu and van der Donk characterizes the role of putative lantibiotic dehydratases in nonribosomal peptide synthetases (NRPSs) and polyketide synthases (PKSs) biosynthetic gene clusters (BGCs). Through the characterization of an NRPS-PKS BGC from Stackebrandtia nassauensis, the authors demonstrate heterologous expression of several metabolites and elucidate the biosynthetic pathway. The final product is identified as a bisarginyl ureidopeptide with an enone electrophile. The study reveals that the putative lantibiotic dehydratase catalyzes peptide bond formation, extending the peptide scaffold in the opposite direction to NRPS and PKS biosynthesis. Furthermore, bioinformatics analysis identifies a distinct active site signature (EHHXXHDG) specific to ureido-generating condensation domains, providing new insights into the versatile biosynthetic mechanisms and enzymes of NRPS.

The manuscript is meticulously written and captivates considerable interest. The presented data are comprehensive, and the clarity of the exposition is commendable. The elucidation of this unusual biosynthetic pathway adds significant value to the research, rendering it well-suited for publication in ACS Central Science. While the manuscript stands out, a few minor concerns and the need for additional explanations have been identified. In particular, providing more information about SnaE and its homologs would enhance the reader's understanding. Additionally, incorporating bioinformatic analyses comparing similar enzymes and delineating differences between lantibiotic dehydratases or PEARLS could contribute valuable insights. Addressing whether there are specific characteristics that could prevent false annotations in the future would further strengthen the manuscript's robustness.

Minor points:

- P. 3 lines 41ff:" In this study, we investigated a hybrid NRPS-PKS BGC22-24 from Stackebrandtia nassauensis that contains a putative lantibiotic dehydratase" The cited references (22-24) are reviews/opinion papers that deal with PKS-NRPS hybrid clusters. There is no information available if the cluster (sna) has previously been described in the literature. If the cluster was previously described, please provide the respective publication. If the cluster is new, please add also information on how the cluster was identified (genome sequencing? antiSMASH?) and provide the respective NCBI accession number for the genome and the location of the cluster.

- P.5, l. 12-16: The authors describe that heterologous expression of the sna BGC led to the three major compounds A, B and C. Furthermore, they suggest that compound C is „likely a later intermediate or the final product "of the BGC (P.6, l. 20-25). Later they consider compound F the final product (P. 7, l. 33-40) that cannot be isolated and tested for bioactivity because of its

instability (P. 12, l. 56 to P. 13, l. 6). Did the authors consider testing the major product C for bioactivity?

- Typo: P. 2, l. 28: missing space in "dehydratases(protein family PF04738)"

-P 5 lines 5-8 and throughout the manuscript: Streptomyces albus J1074 has been reclassified as Streptomyces albidoflavus J1074.

- P 5- 6, Figure 2: I found hard to understand Figure 2 and the corresponding text. Maybe you can clarify the following points: Were compounds A, B, and C all produced, or were they only produced by specific mutants? Why is there an increase in the production of compounds A and B upon SnaO inactivation? Why is there a shift in the retention time for compound A due to SnaE inactivation? Where are compounds D to F in Figure 2? Maybe showing not only the EIC but also total ion chromatogram would help with a better understanding of the results.

- Figure 3 caption: biosynthetic pathway instead of sequence is a more common term. When reading "biosynthetic sequence" I think of actual nucleotide sequences. Maybe consider combining information from Figure 3 and figure S5 for clarity.

- P11: Please also refer to Figure S6A and S6B, not only S6C in the text.

- P12: lines 34ff. Is Methylmalonyl-CoA also predicted to be incorporated by the AT domain in the PKS?

Reviewer: 3

Comments to the Author

In "PEARL-catalyzed peptide bond formation after chain reversal during the biosynthesis of non-ribosomal peptides", Yu and van der Donk perform biochemical and metabolomics studies of biosynthetic gene cluster from Stackebrandtia nassauensis. The authors identify products of the gene cluster and show that the condensation domain of SnaA, the NRPS in the cluster, catalyzes ureido formation. An extended active site motif of EHHxxHDG is discovered to be correlated with ureido-making C domains. Also, they show that SnaE, a putative RIPP dehydratase or putative PEARL enzyme, acts to add Thr to side of the di-Arg ureido that is not extended by the NRPS/PKS assembly line. Overall, this is strong and interesting work that broadens our knowledge of non-canonical functions of biosynthetic enzymes.

These is a disconnect between the emphasis on SnaE in the title and abstract and the relative focus on SnaE / putative lantibiotic dehydratase vs SnaA. Perhaps this is because SnaE is more novel than SnaA's C domain, but SnaE is actually given short schrift in the experimental design, with only

SnaE inactivation investigating its function, and only a statement saying it likely acts like a PEARL. I suggest a change of title and editing of abstract.

The identification of the EHHxxHDG active site is interesting, but I request more information and suggest qualifiers in the discussion.

Are the C domains that have this motif closely evolutionarily related, or has any of them gotten the EHHxxHDG and ureido formation function by convergent evolution?

Is the authors' position that the extra conserved residues in this motif are directly involved in the catalysis? This motif has the residues of the canonical motif present, and adds two extra residues as conserved (EHHxxHDG vs xHHxxxDG). C domain structures show the side chain of the position of the two new additions to the motif (the E and the third H) to next to each other in space, and the H to be far from the catalytic histidine. Is it possible these two residues make a nice salt bridge with each other (and thus are co-evolved) that helps conserve the local structure of the active site, rather than acting in catalysis or substrate binding?

Minor

P2, line ~25: Change "A subset of NRPSs are" to "A small subset of NRPSs are": Two thousand clusters is a large number, but a very small subset of total clusters.

Figure S5: Please alter to make it easier for the reader to quickly understand the transitions between H and D, and especially D and A. Either maintaining the positions of atoms in the molecules, or drawing highlighting reactive groups would help.

Figure 4: It is unclear why the conserved serine is rearely included in the cocnical active site motif, or in this modified motif. Why isn't the ureido C domain's motif "EHHxxHDGxS?

P11, line ~15-18: Modify the sentence "However, the terminal amino acid is left with an unactivated carboxylate and can no longer be extended by the assembly line chemistry" to make more clear which amino acid you mean. Perhaps: "However, the amino acid originally selected the first A domain".

P11, lines 24-28 & Figure S6: Is mechanism c reasonable and worth including? It evokes an adenylation with no precedent that I know, and ends with a symmetrical intermediate that must be selectively hydrolyzed at the T1 (and not equivalent T2) thioester.

Author's Response to Peer Review Comments:

Dear Editor,

Thank you for the reviewer comments. We are pleased by the uniform enthusiasm of the reviewers. We also thank the reviewers for their comments that have allowed us to improve the manuscript. As shown in the response the reviewer file, we have addressed all the comments, either by changes to the text or figures, or by adding new data.

We uploaded a version of the main text and SI in which all our changes are highlighted in yellow.

For reasons that we do not understand, the paragon site has problems converting our Word file to pdf. We have tried to upload from different computers and different versions of the file but there seems to be something that the system does not like but we do not know what. The file in the HTML version looks fine so in the end, we uploaded a pdf version ourselves and are happy to work with someone if our Word version indeed has a problem. For now we wanted to first submit our revised version since we do not know what else to do.

We hope that with our revisions, this manuscript is now suitable for acceptance and publication in ACS Cent Sci.

Thank you for your consideration,

Wilfred

Our responses are in red italics font.

Formatting Needs:

SI File Figures are not labeled (Figure S1, S2, ...).

We assume this comment is in regards to the NMR spectra, which we did not label as figures. All other figures were labeled S1, S2, etc. Assuming that this was the indicated formatting needs, we now labeled those NMR spectra as Figures S14-S30

MS Files: Synopsis is missing.

We have added a synopsis at the end of the manuscript.

Reviewer: 1

Comments:

This manuscript describes a fascinating example of biosynthetic ingenuity and reveals interesting enzyme function both in stand alone enzymes and NRPS domains. The paper is very succinctly written and the figure composition is such that I did find it hard to follow at times (in comparison the discussion is much longer and detailed). I have little complaint with the work as it is performed and it is well documented in the SI - I was somewhat disappointed that no further effort was made in investigating the mechanism of ureido formation as all the pieces are available to help distinguish this fascinating process. Given the ability to identify biosynthesis pathways to find examples of such modified C-domains I think this is an important point to address in more detail. I do not anticipate that this would be overly onerous, however given that this would require new mutants to be generated and tested I therefore chose the major revision option - however with this data included I would be very supportive of the publication of this work in ACS Central Science.

We changed the figures according to the reviewers' suggestions for clarity and flow. We also generated individual PCP mutants as suggested and investigated their in vitro cysteamine-mediated off-loaded intermediates to study the ureido-formation process in more detail (see below).

Specific further points:

There is no mention of C_domains that also for dehydro-residues, would seem something worth adding. The NRPS introduction in general is very brief for a broad audience.

We added a more detailed introduction for the NRPS enzymes and other atypical C domains, including epimerization domains, dual epimerization-condensation domains, C domains that generate dehydroamino acids, and β-lactam formation.

Figure 1 is very light touch - the R groups are not differentiated for example, and this only really captures the C-domain mediated step (no mention of A/TE (or E) domains). As the discussion of ureido-forming C-domains is discussed below, this would also seem worthy of inclusion here for comparison. I don't see how panel C fits with this general figure unless there is a lot more information about the pathway (structures etc), as it is otherwise floating somewhat.

To illustrate the NRPS biosynthetic steps better, we now show 1) adenylation, 2) thiolation, and 3) condensation reactions to capture A, T, and C domain-mediated steps in Figure 1A. We added the schematic description of other C domain-mediated reactions in Figure 1B so that readers can compare the ureido-forming activity with these reactions. As suggested, the previous panel C of Figure 1 (BGC diagram) has been moved to Figure 2A to make the content easier to understand.

Figure 2 is again cut off from the BGC - plus the traces are tiny and colors very hard to differentiate. Also, I thought the mass of C should be 2Da less than B, rather than C (unless there is another difference in the

structures that I could not distinguish?) I think this should be integrated with figure 3 as this helps to also explain the predicted role of the gene products and clarifies the expectations of the deletion experiments.

*We thank the reviewer for the suggestion. We now show the BGC diagram in Figure 2 and combined Figures 2 and 3 to make the experimental data easier to understand. We also remade the EIC traces and offset each trace for clarity. In addition, we added in the figure that the EIC shown is the $[M+2H]^{2+}$. Therefore, the m/z of compound **3** (formerly compound B) is 1 Da less than that of compound **2** (formerly compound C).*

The lack of sterochemistry is problematic. I appreciate that some intermediates are highly prone to cyclization, but why can't the remaining stereochemical centers be distinguished (e.g. by Marfey's analysis)? Given that the manuscript deals with atypical C-domain chemistry I would argue that the existence of dual functional condensation/ epimerase domains makes this a valid question to address.

*We had previously tried to use Marfey's analysis to determine the stereochemistry of Arg and Thr but encountered difficulties. Consistent with previous literature (now mentioned in the SI), D-/L-arginine and L-/L-allo-threonine could not be separated by standard Marfey analysis. After the reviewer comments, we extensively screened columns and mobile phases, and we identified HPLC conditions to separate the two arginine and four threonine isomers. In the main text, we added a description that the Arg and Thr both have L configuration. The supporting data is presented in new Figures S4-S5. The unsaturated derivative of arginine in compounds **4-6** (formerly compounds D-F) loses the stereocenter upon conversion to compounds **1-3** (formerly compounds A-C). Therefore, the stereochemistry of one of the initially incorporated Arg cannot be determined at present.*

Figure 4 panel A should be condensed/ show as a weblogo type image for clarity. How do such motifs compare to other examples of C-domain motifs (b-lactam formation etc)? What other intermediates are offloaded in these experiments (especially for the mutants) – this would help to clarify activation of substrates.

We condensed the sequence alignment to a weblogo as suggested and have added Table S4 to compare the C_{urea} domain active site motif with other C domains, such as ${}^{L/D}C_L$, E, C_{modAA}, β-lactam formation, etc. We searched for potential intermediates, such as N-carboxy arginine, that are offloaded by cysteamine but did not observe them in LC-MS experiments. We believe that N-carboxy arginine and other intermediates only exist transiently in the active site of SnaA and cannot be intercepted. It they are intercepted then once off-loaded from the PCP, the carboxy group likely spontaneously reverts to CO_2/bicarbonate.

The discussion of the mechanism I found somewhat confusing - as there is access to the protein encoding SnaA it would be possible to distinguish the mechanisms in figure S6. The loading of multiple PCP domains by one A-domain has been reported, and thus I would think that the specificity of these two A-domains could be distinguished (ie is one activating bicarbonate and one loading both Arg residues?) Inspection of the substrate selection pockets of these A-domains would also yield valuable clues as to the likely substrates of these domains. Inactivation of PCP domains by Ser to Ala mutation would also allow intermediates to be isolated that would help to then show the timing of bicarbonate addition and to which Arg residue. I found it a little odd to see the discussion engaging with A-domain specificity from other pathways without explicitly addressing this for the system under investigation. Given the ability to assay C-domain mutants I would expect that these experiments should also prove feasible and relatively straightforward to accomplish and this would be a major contribution to this field.

We thank the reviewer's suggestions for improving the study. In the current version, we added a discussion of the A domain specificity in the "Proposed Biosynthetic Pathway" section. Various bioinformatics tools, such as antiSMASH, PRISM, and AdenylPred, gave different predictions for both of the A domains of SnaA. AntiSMASH and PRISM predict that both A domains will activate hydrophobic amino acids. AdenylPred predicts that both A domains will activate hydrophilic and charged amino acids, which is consistent with the observed product from the sna BGC. These different predictions suggest that the A domain substrate selection pockets are unlike characterized examples, and an accurate prediction is not made. However, all these software packages predict that both A domains will activate the same type of amino acids. Therefore, we believe that both A domains of SnaA are likely to activate arginine and that it is unlikely for one of them to activate bicarbonate. As we mentioned in the revised manuscript, for other natural products the substrate prediction of the A domains that sandwich the C_{urea} domains in modular NRPSs is consistent with the two amino acids observed in the ureido group of the final product. The activation of CO_2/bicarbonate by one of the A

domains is less likely but is not completely ruled out currently.

We also generated and tested the T domain mutants in vitro, as the reviewer suggested. Inactivation of either T domain by mutation of the Ser that is loaded with phosphopantetheine abolished product formation, showing that both T domains are required for the ureido formation activity of SnaA. Cysteamine-intercepted arginine was observed for both mutants, which strongly suggests that arginine is loaded onto both T domains. We searched for a bicarbonate adduct of arginine but did not observe it in the LC-MS experiments. We believe that the N-carboxy arginine is an unstable intermediate that will convert to CO_2 and arginine spontaneously outside the enzyme's active site.

Is there any MbtH protein in the producer? Are these A-domains happy without one for in vitro experiments?

We apologize for not making this more clear in the original submission. The SnaC gene encodes an MbtH-like protein (MLP), and we had performed all in vitro assays of SnaA in the presence of SnaC. In the revised version, we have annotated SnaC as an MLP in Figure 2A and added a description of each gene encoded in the sna BGC that will help clarification. We tested the in vitro activity of SnaA without SnaC and observed little to no adenylation activity demonstrated by the detection of cysteamineintercepted arginine. Consequently, there was also no formation of the bisarginyl ureido dipeptide in the absence of SnaC. Therefore, we believe that the A domains in SnaA need the presence of SnaC for robust activity in vitro. We now made it clear in the "Bioinformatic and Biochemical study on ureido group formation" section that the MLP SnaC is required for the in vitro activity of SnaA.

My experience with chemistry journals leads me to have a preference for numbers for compounds rather than letters - especially when using many gene/protein names and many motif signatures that are all letters.

We have changed the nomenclature of compounds A-F to compounds 1-6 according to the reviewer's suggestion.

There are some legibility issues with figure S4.

We initially were not sure what the reviewer referred to, but after looking at the pdf version of the SI, we see that indeed the figure had changed significantly upon pdf generation. We have made sure that for this revised submission the pdf was correct and appreciate the reviewer pointing iout the issue.

Data in Figure S1 would be much easier to read when tabulated (as with S2/S3).

We have arranged the expected m/z and the ppm-error of each fragment ion into a table to make it easier to read in Figures S1-3.

We thank the reviewer for their constructive criticism that has improved the manuscript.

Reviewer: 2

Recommendation: Publish in ACS Central Science after minor revisions noted.

Comments:

The manuscript by Yu and van der Donk characterizes the role of putative lantibiotic dehydratases in nonribosomal peptide synthetases (NRPSs) and polyketide synthases (PKSs) biosynthetic gene clusters (BGCs). Through the characterization of an NRPS-PKS BGC from Stackebrandtia nassauensis, the authors demonstrate heterologous expression of several metabolites and elucidate the biosynthetic pathway. The final product is identified as a bisarginyl ureidopeptide with an enone electrophile. The study reveals that the putative lantibiotic dehydratase catalyzes peptide bond formation, extending the peptide scaffold in the opposite direction to NRPS and PKS biosynthesis. Furthermore, bioinformatics analysis identifies a distinct active site signature (EHHXXHDG) specific to ureido-generating condensation domains, providing new insights into the versatile biosynthetic mechanisms and enzymes of NRPS.

The manuscript is meticulously written and captivates considerable interest. The presented data are comprehensive, and the clarity of the exposition is commendable. The elucidation of this unusual biosynthetic pathway adds significant value to the research, rendering it well-suited for publication in ACS Central Science. While the manuscript stands out, a few minor concerns and the need for additional explanations have been identified. In particular, providing more information about SnaE and its homologs would enhance the reader's understanding. Additionally, incorporating bioinformatic analyses comparing similar enzymes and delineating differences between lantibiotic dehydratases or PEARLS could contribute valuable insights. Addressing whether there are specific characteristics that could prevent false annotations in the future would further strengthen the manuscript's robustness.

In the discussion section, we added a bioinformatic comparison of SnaE, PEARLs, and lantibiotic dehydratases to illustrate their similarities and differences. The supporting data is presented in Fig S13. PEARLs have additional conserved residues for their phosphorylation activity (as identified in other studies now cited) compared to the dehydratases. Therefore, sequence analysis can distinguish PEARLs from lantibiotic/thiopeptide dehydratases. At present, the homologs of SnaE, that work with assembly-line enzymes are not distinguishable from PEARLs in their enzyme sequences. Genome neighborhood analysis

is needed to prevent false annotation of PEARLs and SnaE homologs. We have added text to make this clear.

Minor points:

- P. 3 lines 41ff:" In this study, we investigated a hybrid NRPS-PKS BGC22-24 from Stackebrandtia nassauensis that contains a putative lantibiotic dehydratase" The cited references (22-24) are reviews/opinion papers that deal with PKS-NRPS hybrid clusters. There is no information available if the cluster (sna) has previously been described in the literature. If the cluster was previously described, please provide the respective publication. If the cluster is new, please add also information on how the cluster was identified (genome sequencing? antiSMASH?) and provide the respective NCBI accession number for the genome and the location of the cluster.

The cited references included reference 23 (Singh et al) that was not a review but a research article that bioinformatically analyzed the BGC. We have made it more clear that the gene cluster was first identified in this paper by Singh et al. (now main text reference 33) in the "Products generated by the sna BGC" section.

- P.5, l. 12-16: The authors describe that heterologous expression of the sna BGC led to the three major compounds A, B and C. Furthermore, they suggest that compound C is „likely a later intermediate or the final product "of the BGC (P.6, l. 20-25). Later they consider compound F the final product (P. 7, l. 33-40) that cannot be isolated and tested for bioactivity because of its instability (P. 12, l. 56 to P. 13, l. 6). Did the authors consider testing the major product C for bioactivity?

We tested compound 3 (formerly compound C) for bioactivity against Lactococcus lactis subsp. cremoris, Bacillus subtilis, Micrococcus luteus, and E. coli MG1655. No growth inhibition was observed up to 1 mM concentration of compound 3. We have added this information to the main text discussion section.

-Typo: P. 2, l. 28: missing space in "dehydratases(protein family PF04738)" *We have*

fixed this in the revised manuscript.

-P 5 lines 5-8 and throughout the manuscript: Streptomyces albus J1074 has been reclassified as Streptomyces albidoflavus J1074.

Thank you for informing us of this reclassification. We have updated the species name throughout the manuscript and SI.

- P 5- 6, Figure 2: I found hard to understand Figure 2 and the corresponding text. Maybe you can clarify the following points: Were compounds A, B, and C all produced, or were they only produced by specific mutants? Why is there an increase in the production of compounds A and B upon SnaO inactivation? Why is there a shift in the retention time for compound A due to SnaE inactivation? Where are compounds D to F in Figure 2? Maybe showing not only the EIC but also total ion chromatogram would help with a better understanding of the results.

Compounds 1, 2, and 3 (formerly A, B, and C) were all produced when the BGC was heterologously expressed. Compounds 1 and 2 were produced by the SnaO inactivation mutant. For the SnaE inactivation mutant, only compound 1 is produced. We believe that SnaO acts on compounds 7 and 8 (Figure 3).

Therefore, upon SnaO inactivation, compounds 1, 2, 4 and 5 will accumulate due to spontaneous decarboxylation or further cyclization from the accumulated compounds 7 and 8.

Hydrophilic interaction chromatography (HILIC) is known to exhibit less reproducible retention times compared to reversed-phase HPLC. The observed retention time difference in the previous version of Figure 2 is due to the retention time drift of samples analyzed on different days. To reduce confusion for readers, we reran the samples at the same time to align their retention times. Compounds 4-6 (formerly D-F) were originally shown in Figure 3 but are now moved to Figure 2 to help readers better understand all the metabolites produced. We decided not to show the TIC since the ions of interest are obscured by many MS features from the medium.

- Figure 3 caption: biosynthetic pathway instead of sequence is a more common term. When reading "biosynthetic sequence" I think of actual nucleotide sequences. Maybe consider combining information from Figure 3 and figure S5 for clarity.

We have changed "biosynthetic sequence" throughout the manuscript to "biosynthetic pathway." We have also combined Figure 3 and Figure S5.

- P11: Please also refer to Figure S6A and S6B, not only S6C in the text.

We have added a brief description of the mechanisms in Fig S11A and Fig. S11B (formerly Fig. S6AB) in the discussion section.

- P12: lines 34ff. Is Methylmalonyl-CoA also predicted to be incorporated by the AT domain in the PKS?

AntiSMASH predicts malonyl-CoA as the substrate of the AT domain in SnaB. We added the discussion of SnaB's predicted substrate specificity.

We thank the reviewer for their constructive criticism that has improved the manuscript.

Reviewer: 3

Recommendation: Publish in ACS Central Science after minor revisions noted.

Comments:

In "PEARL-catalyzed peptide bond formation after chain reversal during the biosynthesis of nonribosomal peptides", Yu and van der Donk perform biochemical and metabolomics studies of biosynthetic gene cluster from Stackebrandtia nassauensis. The authors identify products of the gene cluster and show that the condensation domain of SnaA, the NRPS in the cluster, catalyzes ureido formation. An extended active

site motif of EHHxxHDG is discovered to be correlated with ureido-making C domains. Also, they show that SnaE, a putative RIPP dehydratase or putative PEARL enzyme, acts to add Thr to side of the di-Arg ureido that is not extended by the NRPS/PKS assembly line. Overall, this is strong and interesting work that broadens our knowledge of non-canonical functions of biosynthetic enzymes.

These is a disconnect between the emphasis on SnaE in the title and abstract and the relative focus on SnaE / putative lantibiotic dehydratase vs SnaA. Perhaps this is because SnaE is more novel than SnaA's C domain, but SnaE is actually given short schrift in the experimental design, with only SnaE inactivation investigating its function, and only a statement saying it likely acts like a PEARL. I suggest a change of title and editing of abstract.

We agree and changed the title to "PEARL-catalyzed peptide bond formation after chain reversal by ureido-forming condensation domains" to emphasize more the ureido structure forming domains which we analyzed bioinformatically and biochemically. The annotation of function of SnaE was our initial goal and it could be argued that it is the most novel part of the study, so we also kept it in the title. We have also added a new paragraph in the discussion section to better illustrate the similarities and differences between SnaE, PEARLs, and lantibiotic dehydratases to help readers better understand commonalities and differences between these enzymes.

The identification of the EHHxxHDGl active site is interesting, but I request more information and suggest qualifiers in the discussion.

We added more information on how the C_{urea} group of C domains can be identified by adding a phylogenetic tree of C domains associated with ureido-containing NRPs. The C_{urea} sequences we used for the tree construction and multiple sequence alignment are available in a supplemental file so that others in the field can make future predictions of C_{urea} domains by inspecting the active site signature or the grouping within the phylogenetic tree.

Are the C domains that have this motif closely evolutionarily related, or has any of them gotten the EHHxxHDG and ureido formation function by convergent evolution?

We have constructed a phylogenetic tree of C domains associated with BGCs that are known to produce ureido-containing NRPs. In addition to C_{urea} domains, these BGCs contain $^{L}C_{L}$, $^{D}C_{L}$, Cy, E, and Dual C domains. The phylogenetic tree (Fig. S7) shows that different C domains group according to their catalytic functions. This observation based on currently characterized BGCs suggests that C_{urea} domains are evolutionarily related. We did not make a conclusion whether these domains evolved convergently as we feel that a more detailed bioinformatic analysis is needed to make this conclusion definitively. We do show the tree so readers can decide whether they think the evolution is convergent or not.

Is the authors' position that the extra conserved residues in this motif are directly involved in the catalysis? This motif has the residues of the canonical motif present, and adds two extra residues as conserved (EHHxxHDG vs xHHxxxDG). C domain structures show the side chain of the position of the two new additions to the motif (the E and the third H) to next to each other in space, and the H to be far from the catalytic histidine. Is it possible these two residues make a nice salt bridge with each other (and thus are co-evolved) that helps conserve the local structure of the active site, rather than acting in catalysis or substrate binding?

We do not know the exact function of these two residues. They might be involved in catalysis but as the reviewer notes, there are also other alternative possible explanations. During the revisions, we made an AlphaFold model of the C_{urea} domain of SnaA. The extra conserved E and H are indeed predicted to form a salt bridge. We added this alternative explanation for their conservation in the main text and present the AlphaFold model in Fig. S8.

Minor

P2, line ~25: Change "A subset of NRPSs are" to "A small subset of NRPSs are": Two thousand clusters is a large number, but a very small subset of total clusters. *We agree and have changed the corresponding sentence in the main text.*

Figure S5: Please alter to make it easier for the reader to quickly understand the transitions between H and D, and especially D and A. Either maintaining the positions of atoms in the molecules, or drawing highlighting reactive groups would help.

*We thank the reviewer's suggestions for clarity. We have combined the previous Fig. S5 with Fig. 3 in the main text, as other reviewers have suggested, for the readers to better understand the relationships between the molecules. We maintained the positions of functional groups that did not undergo transformation for compound **7** (formerly H), **4** (formerly D), and **1** (formerly A).*

Figure 4: It is unclear why the conserved serine is rearely included in the cocnical active site motif, or in this modified motif. Why isn't the ureido C domain's motif "EHHxxHDGxS?

In the first identification of C domains in NRPSs by de Crécy-Lagard et al., the HHXXXDG motif was identified by the authors as the signature of domains that catalyze peptide elongation. Since C domains structurally resemble and share the same HHXXXDG motif with chloramphenicol acetyltransferases (CAT), the HHXXXDG motif signature is widely adopted by the field to indicate a potentially common mechanism shared with CAT. The reviewer is correct that the additional Ser is also always conserved but that it is not usually included in prior descriptions of conserved motifs. We considered the comment of the reviewer and in the end decided to omit the "xS" in the conserved EHHXXXDGXS motif for two reasons. First to maintain consistency with the extensive literature prior to our study. Second, this serine is conserved in both C_{urea} domains and amide-forming C domains in ureido-containing nonribosomal peptide BGCs. Therefore, the conserved "xS" motif is not specific to C_{urea} domains, and we did not want to inadvertently imply to some readers that by including it when it is usually not provided this Ser is a specific feature of the ureido-forming C domains. Importantly, we do show this Ser in the logo in Fig. 4A so the information is there. We also added a comment to the legend of Fig 4.

P11, line ~15-18: Modify the sentence "However, the terminal amino acid is left with an unactivated carboxylate and can no longer be extended by the assembly line chemistry" to make more clear which amino acid you mean. Perhaps: "However, the amino acid originally selected the first A domain".

We thank the reviewer for the suggestion and have changed the corresponding sentence in the main text.

P11, lines 24-28 & Figure S6: Is mechanism c reasonable and worth including? It evokes an adenylation with no precedent that I know, and ends with a symmetrical intermediate that must be selectively hydrolyzed at the T1 (and not equivalent T2) thioester.

Mechanism C is indeed less likely than mechanisms A and B, because there is no evidence that supports the adenylation of the N-carboxy group and the selective hydrolysis of one thioester after product formation. However, although it is a less likely mechanism, mechanism C is still a possibility that the current data cannot rule out.

Furthermore, we initially added mechanism C because it shows an alternative means to recruitment of a PEARL to make a ureido product with chain extension of the amino acid on both sides of the urea. We added some text to the legend to better explain this (note that it was this aspect of mechanism C that we highlighted in the original submission, albeit apparently not very effectively).

We therefore prefer to keep mechanism C in the SI.

We thank the reviewer for their constructive criticism that has improved the manuscript.

oc-2024-000445.R2

Name: Peer Review Information for "PEARL-catalyzed peptide bond formation after chain reversal by ureido-forming condensation domains"

Second Round of Reviewer Comments

Reviewer: 3

Comments to the Author

It's a yes from me.

Reviewer: 1

Comments to the Author

The authors have made a very comprehensive effort to address the constructive suggestions from the reviewers - given this has added to the quality of what was already an impressive piece of work, I have no hesitation in supporting the acceptance of this current version for publication in ACS Central Science.

Author's Response to Peer Review Comments:

We made the requested changes to the title page.

Thanks for handling this manuscript.

Wilfred
